# NrCAM is a marker for substrate‐selective activation of ADAM10 in Alzheimer's disease

**DOI:** 10.15252/emmm.201809695

**Published:** 2019-03-04

**Authors:** Tobias Brummer, Stephan A Müller, Francisco Pan‐Montojo, Fumiaki Yoshida, Andreas Fellgiebel, Taisuke Tomita, Kristina Endres, Stefan F Lichtenthaler

**Affiliations:** ^1^ Deutsches Zentrum für Neurodegenerative Erkrankungen (DZNE) Munich Germany; ^2^ Neuroproteomics School of Medicine Klinikum rechts der Isar Technische Universität München Munich Germany; ^3^ Munich Cluster for Systems Neurology (SyNergy) Munich Germany; ^4^ Department of Neurology Ludwig‐Maximilians‐University Munich Munich Germany; ^5^ Laboratory of Neuropathology and Neuroscience Graduate School of Pharmaceutical Sciences The University of Tokyo Tokyo Japan; ^6^ Department of Psychiatry and Psychotherapy University Medical Center JGU Mainz Germany; ^7^ Institute for Advanced Study Technische Universität München Garching Germany

**Keywords:** acitretin, ADAM10, Alzheimer's disease, cerebrospinal fluid proteomics, NrCAM, Biomarkers & Diagnostic Imaging, Neuroscience

## Abstract

The metalloprotease ADAM10 is a drug target in Alzheimer's disease, where it cleaves the amyloid precursor protein (APP) and lowers amyloid‐beta. Yet, ADAM10 has additional substrates, which may cause mechanism‐based side effects upon therapeutic ADAM10 activation. However, they may also serve—in addition to APP—as biomarkers to monitor ADAM10 activity in patients and to develop APP‐selective ADAM10 activators. Our study demonstrates that one such substrate is the neuronal cell adhesion protein NrCAM. ADAM10 controlled NrCAM surface levels and regulated neurite outgrowth *in vitro* in an NrCAM‐dependent manner. However, ADAM10 cleavage of NrCAM, in contrast to APP, was not stimulated by the ADAM10 activator acitretin, suggesting that substrate‐selective ADAM10 activation may be feasible. Indeed, a whole proteome analysis of human CSF from a phase II clinical trial showed that acitretin, which enhanced APP cleavage by ADAM10, spared most other ADAM10 substrates in brain, including NrCAM. Taken together, this study demonstrates an NrCAM‐dependent function for ADAM10 in neurite outgrowth and reveals that a substrate‐selective, therapeutic ADAM10 activation is possible and may be monitored with NrCAM.

## Introduction

Alzheimer's disease (AD) is the most common neurodegenerative disorder and affects over 40 million patients worldwide (Scheltens *et al*, 2016; Selkoe & Hardy, 2016), but no causative or preventive treatment is currently available. One drug target in AD is the metalloprotease “a disintegrin and metalloprotease 10” (ADAM10), which acts as α‐secretase and cleaves the amyloid precursor protein (APP) within the amyloid β (Aβ) domain (Lammich *et al*, 1999; Jorissen *et al*, 2010; Kuhn *et al*, 2010). Thus, ADAM10 cleavage of APP has the ability to prevent the generation of the pathogenic Aβ peptide. In fact, neuronal overexpression of wild‐type ADAM10 in mouse brain prevents amyloid pathology whereas catalytically inactive ADAM10 suppresses α‐secretase activity and consequently enhances amyloid pathology (Postina *et al*, 2004). Likewise, reduction of ADAM10 activity by interfering with ADAM10 trafficking enhanced amyloid pathology in mice (Epis *et al*, 2010). Importantly, rare, partial loss‐of‐function mutations of ADAM10 are genetically linked to late‐onset AD in patients (Kim *et al*, 2009). This reinforces the notion that an activation of ADAM10 may be beneficial to treat or even prevent AD and has led to a first, phase 2 clinical trial with AD patients treated over 4 weeks with the ADAM10 activator acitretin (Endres *et al*, 2014). Acitretin is a second generation retinoid, is in clinical use to treat the skin disease psoriasis, and activates ADAM10 expression *in vitro* and in mice (Tippmann *et al*, 2009; Reinhardt *et al*, 2016). Importantly, in the small, proof‐of‐principle phase 2 study, acitretin indeed increased the ADAM10‐mediated cleavage of APP (Endres *et al*, 2014). An in‐depth neuropsychiatric examination was not performed in the patients and awaits a future study of longer duration and with a larger patient cohort. No major unwanted side effects were reported. Despite the positive outcome of the acitretin study, a key question is the safety profile of prolonged ADAM10 activation. This is of particular concern, because ADAM10 cleaves numerous other substrates besides APP—generally referred to as ectodomain shedding (Lichtenthaler *et al*, 2018)—, both during embryonic development and in different adult tissues, including the adult brain (Saftig & Lichtenthaler, 2015; Kuhn *et al*, 2016). One of its major substrates *in vivo* is the Notch receptor, which requires ADAM10 cleavage for its ligand‐induced signal transduction (Pan & Rubin, 1997; Bozkulak & Weinmaster, 2009; van Tetering *et al*, 2009). Other substrates include cell adhesion proteins, e.g., NCAM and N‐cadherin (Reiss *et al*, 2005; Hinkle *et al*, 2006), and growth factors and signaling proteins, such as neuregulin‐1 (Freese *et al*, 2009), epidermal growth factor (Sahin *et al*, 2004), death receptor 6 (Colombo *et al*, 2018), and APLP2 (Endres *et al*, 2005; Hogl *et al*, 2011). Thus, the intended activation of ADAM10 for a prevention or treatment of AD may induce mechanism‐based side effects by interfering with the cleavage and physiological function of other ADAM10 substrates. While this has not yet been tested systematically, a precedent is seen for γ‐secretase inhibitors, which were discontinued in clinical trials for AD as they led to mechanism‐based toxicity upon prolonged dosing (Golde *et al*, 2013). These inhibitors did not only block Aβ generation but also cleavage of additional γ‐secretase substrates, including Notch.

Additional ADAM10 substrates are not only a concern, but also offer chances for drug development. Their cleavage products may be detected in body fluids, such as plasma and cerebrospinal fluid (CSF), and may potentially be used as companion diagnostics, i.e., as surrogate markers to monitor ADAM10 activity *in vivo*, similar to what has been suggested for the β‐secretase BACE1 (Pigoni *et al*, 2016). Likewise, the additional substrates may be used for the development of substrate‐selective ADAM10 activators that preferentially stimulate APP processing over the cleavage of other ADAM10 substrates.

Here, using CSF from a phase II clinical trial, we demonstrate that a substrate‐selective activation of ADAM10 is feasible in patients and may be safer than expected. Moreover, we show that the ADAM10 substrate “neural glial‐related cell adhesion molecule” (NrCAM) is an excellent marker for selective ADAM10 activation *in vivo*. NrCAM belongs to the L1 family of IgCAMs and is a cell adhesion molecule (Grumet *et al*, 1991), which controls dendritic spine densities, axonal guidance, and targeting as well as neurite outgrowth, by acting as a co‐receptor molecule at the neuronal cell surface (Falk *et al*, 2004; Zelina *et al*, 2005; Nawabi *et al*, 2010; Torre *et al*, 2010; Demyanenko *et al*, 2011, 2014; Kuwajima *et al*, 2012; Dai *et al*, 2013). Soluble NrCAM (sNrCAM) is reduced in the CSF of AD patients compared to healthy controls (Hu *et al*, 2010; Wildsmith *et al*, 2014). In a previous proteomic study, NrCAM was discovered as an ADAM10 substrate *in vitro* (Kuhn *et al*, 2016). Now, we demonstrate that ADAM10 controls neuronal surface levels of NrCAM and neurite outgrowth in an NrCAM‐dependent manner. Importantly, our study shows that activation of NrCAM cleavage by ADAM10 occurs through different mechanisms compared to APP, making soluble NrCAM an excellent marker for developing therapeutic, APP‐selective, or APP‐preferring ADAM10 activators.

## Results

### Proteolytic processing of NrCAM by furin, ADAM10, and γ‐secretase

#### ADAM10 but not ADAM17 cleaves NrCAM in primary neurons

**Figure 1:**
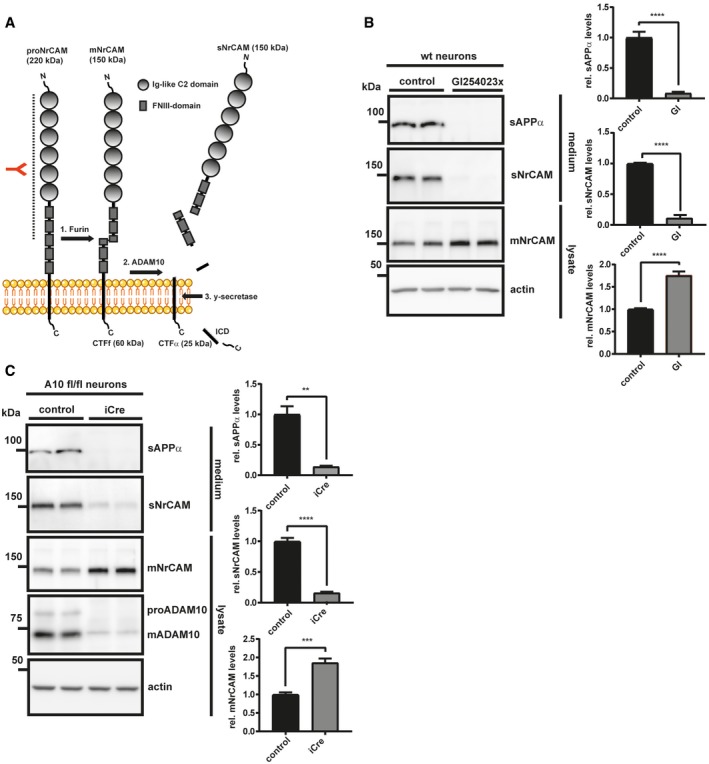
ADAM10 is required for NrCAM shedding in primary neurons ASchematic diagram of NrCAM's domain structure and sequential proteolytic processing. NrCAM is firstly cleaved by furin, then by ADAM10, and finally by the γ‐secretase. The red antibody indicates the binding region of the N‐terminal NrCAM antibody. ICD (intracellular domain), CTFf (C‐terminal fragment created by furin cleavage), CTFα (C‐terminal fragment created by ADAM10 cleavage).BDetection of soluble, cleaved NrCAM (sNrCAM) and full‐length, mature NrCAM (mNrCAM) and soluble APPα (sAPPα) in neuronal supernatants and lysates (prepared from E16 neurons), treated with GI254023x (5 μM), or solvent for 48 h.CDetection of sAPPα, sNrCAM, and mNrCAM in ADAM10 cKO neuronal supernatants and lysates at 7 days *in vitro* (DIV7). The neurons prepared from floxed ADAM10 (ADAM10fl/fl) mice were infected with a lentivirus encoding improved Cre recombinase (iCre) or a control GFP lentivirus at DIV2. Conditioned media were collected for 48 h.Data information: In (B and C), densitometric quantifications of the Western blots are shown on the right (**P < 0.01; ***P < 0.001; ****P < 0.0001, two‐sided Student's *t*‐test, *n* = 6–8). Given are mean ± the standard error of the mean. The mean levels of solvent‐treated cells were set to 1. Representative Western blots are shown.Source data are available online for this figure.

**Figure EV1:**
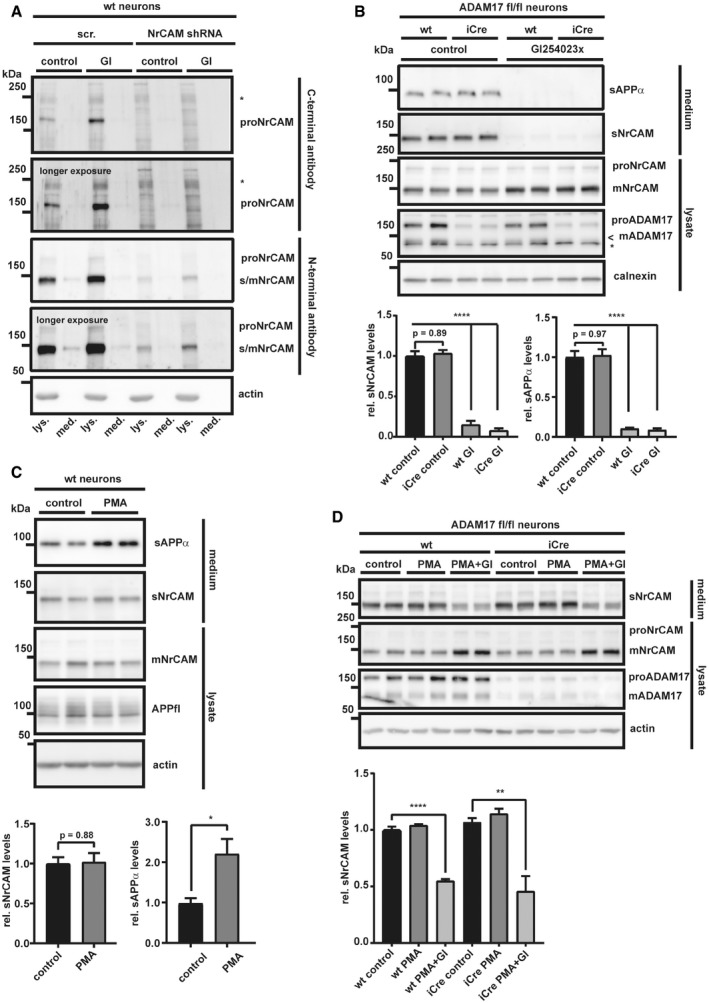
NrCAM is neither a constitutive nor a stimulated ADAM17 substrate ALysates (lys.) and conditioned media (med.) from wt and NrCAM knock‐down neurons, both treated with either GI254023X (5 μM) or solvent, were analyzed next to each other. The C‐terminal antibody only recognized proNrCAM, while the N‐terminal NrCAM antibody detected both mNrCAM, proNrCAM, and sNrCAM in the conditioned media. * indicates an unspecific band.BADAM17fl/fl neurons were treated with an iCre virus, inducing the ADAM17 knock‐out, or a control lentivirus at DIV2. The cells were kept in culture until DIV5; then, the neurons were treated with the ADAM10‐preferring inhibitor GI254023x (5 μM) or solvent (control). After 24 h, the cells were lysed and the conditioned media were collected. * indicates an unspecific band; < indicates the band of mADAM17.CWt neurons were kept in culture until DIV5; then, the cells were treated for 3 h with PMA (1 μM) alone, PMA (1 μM) and GI254023x (5 μM), or solvent.DADAM17fl/fl neurons were prepared like in (B). At DIV5, the neurons were treated with PMA (1 μM) alone, PMA (1 μM) and GI254023x (5 μM), or solvent for 3 h. In contrast to other ADAM10 substrates (e.g., L1CAM, APP), the phorbol ester PMA did not stimulate NrCAM cleavage.Data information: In (B–D), densitometric quantifications of the Western blots are shown below. One‐way ANOVA with *post hoc* Dunnett's test for (B and D), or two‐sided Student's *t*‐test for (C) (*P < 0.05; **P < 0.01; ****P < 0.0001, *n* = 4–6). Shown are mean and SEM. The mean levels of solvent‐treated cells were set to 1. Representative Western blots are shown.Source data are available online for this figure.

To determine whether the cleaved, soluble ectodomain of NrCAM (for a schematic, see Fig 1A) may be a suitable biomarker for ADAM10 activity, we first analyzed in detail the proteolytic processing of NrCAM in cultured neurons. The ADAM10‐preferring metalloprotease inhibitor GI254023X (Hundhausen *et al*, 2003; Ludwig *et al*, 2005) (Fig 1B) as well as the conditional knock‐out of ADAM10 (Fig 1C) abolished secretion of the shed NrCAM ectodomain (sNrCAM), as well as of sAPPα into the conditioned medium of primary, murine neurons and increased full‐length, mature NrCAM (mNrCAM) levels in the neuronal lysate. The protein bands were specific for sNrCAM and mNrCAM as demonstrated with shRNA knock‐down experiments of NrCAM and using different antibodies (Fig EV1A). This demonstrates that NrCAM is a substrate for ADAM10 in neurons and is consistent with previous studies using cultured neurons *in vitro* (Kuhn *et al*, 2016; Brummer *et al*, 2018). In contrast, the conditional knock‐out of ADAM17, a close homolog of ADAM10, did not alter NrCAM proteolysis in primary neurons (Fig EV1B–D), demonstrating the specific requirement of ADAM10 for NrCAM shedding. Taken together, we conclude that NrCAM is a substrate for ADAM10, which, unlike other substrates (e.g., APP, L1CAM), is not additionally cleaved by ADAM17.

#### NrCAM is processed to a heterodimer and then cleaved by ADAM10

**Figure EV2:**
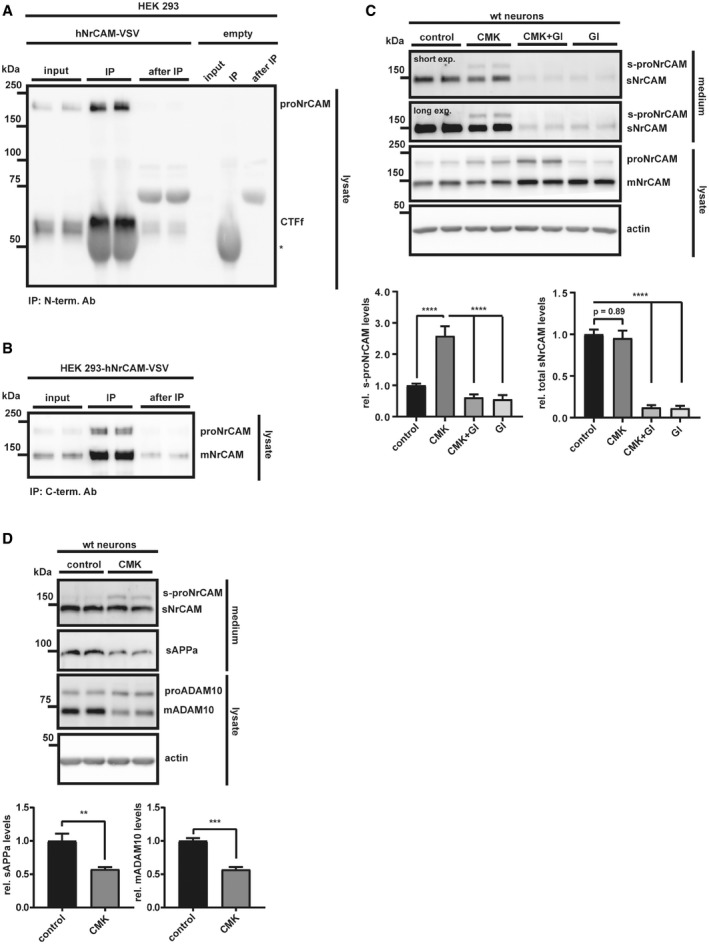
C‐ and N‐terminal NrCAM fragments stay attached after initial furin cleavage ACo‐immunoprecipitation of C‐ and N‐terminal NrCAM fragments in lysates of HEK 293 cells transfected with a C‐terminally VSV‐tagged NrCAM construct, or empty vector. Cells were lysed in CoIP buffer. IP was performed with an N‐terminal NrCAM antibody, detection with a C‐terminal VSV antibody. The CTFf (CTFfurin) band was running a little bit higher than in the input control, presumably because of the abundant IgG heavy chain, running right beneath it. Because of the lack of a suitable antibody, we were not able to detect the small fragment between the furin and the ADAM10 cleavage site. * indicates the IgG heavy chain.BCo‐immunoprecipitation like in A, but the IP was done with a C‐terminal VSV antibody and the detection with an N‐terminal NrCAM antibody. NrCAM was detected as non‐cleaved proNrCAM and the furin‐cleaved mature mNrCAM.CTo test whether NrCAM was sequentially cleaved by furin and then ADAM10, we treated primary neurons with the furin inhibitor dec‐CMK (50 μM), GI254023x (5 μM), or solvent for 48 h. Furin inhibition did not block NrCAM shedding, but caused the appearance of a 170 kDa band in the conditioned media, which was inhibited by GI254023x. In addition, dec‐CMK increased the 220 kDa proNrCAM (but not mNrCAM), which was even further increased by GI254023x. The levels of total sNrCAM remained unaffected by the furin inhibition. Densitometric quantifications of the Western blots are shown. One‐way ANOVA with *post hoc* Dunnett's test (****P < 0.0001, *n* = 6). Given are mean ± the standard error of the mean. The mean levels of solvent‐treated cells were set to 1.DNeurons were treated with dec‐CMK or solvent like in (C). The small decrease in mADAM10 levels significantly reduced sAPPα, but not total sNrCAM release. Densitometric quantifications of the Western blots are shown. Two‐sided Student's *t*‐test (**P < 0.01; ***P < 0.001, *n* = 4). Given are mean ± the standard error of the mean. The mean levels of solvent‐treated cells were set to 1. Together, these results show that ADAM10 is not only able to cleave both mNrCAM and proNrCAM, but is also the protease to release sNrCAM into the conditioned media. Thus, NrCAM is firstly cleaved by furin and then by ADAM10, releasing sNrCAM. Representative Western blots are shown. Source data are available online for this figure.

The ADAM10‐cleaved sNrCAM lacks the transmembrane and cytoplasmic domains of full‐length, mature mNrCAM (Fig 1A) and thus should have a lower apparent molecular weight on immunoblots than mNrCAM in the lysate. Yet, both had the same apparent molecular weight of 150 kDa (Fig 1A). This likely results from a previously described furin protease cleavage site located within NrCAM's third FNIII domain (scheme in Fig 1A) (Kayyem *et al*, 1992; Davis & Bennett, 1994; Susuki *et al*, 2013) and, thus, further away from the membrane than the membrane‐proximal ADAM10 cleavage site. The furin cleavage is expected to occur in the secretory pathway, converting the full‐length pro‐form of NrCAM (proNrCAM) to the cleaved mature mNrCAM. As a result, mNrCAM may be present as a heterodimer consisting of the large N‐terminal ectodomain fragment that ends at the furin site, and a C‐terminal fragment (CTFf) that spans from the furin cleavage site to the cytoplasmic C‐terminus of NrCAM (Fig 1A). In fact, immunoprecipitation of NrCAM with an N‐terminally binding antibody co‐precipitated the CTFf fragment (Fig EV2A). Conversely, immunoprecipitation of NrCAM with a C‐terminally binding antibody co‐precipitated the N‐terminal furin‐cleaved NrCAM ectodomain (Fig EV2B). Together, these results demonstrate that mNrCAM is a heterodimer of a 150 kDa N‐terminal ectodomain fragment and the 60 kDa CTFf fragment. A similar heterodimer formation is known for Notch1, another ADAM10 substrate (Sanchez‐Irizarry *et al*, 2004).

The furin inhibitor dec‐RVKR‐CMK reduced maturation of NrCAM in primary murine neurons, but did not affect shedding (Fig EV2C), ruling out an involvement of furin in NrCAM shedding. Moreover, only the ectodomain of the mature, furin‐cleaved NrCAM was detected in the conditioned medium of untreated controls. In agreement with previous publications (Anders *et al*, 2001; Lopez‐Perez *et al*, 2001), dec‐RVKR‐CMK decreased both mADAM10 and sAPPα levels (Fig EV2D). Additionally, it decreased sNrCAM, which results from furin cleavage, and increased instead of the cleavage of the non‐furin‐cleaved soluble form of NrCAM (s‐proNrCAM). Thus, total sNrCAM (sNrCAM + s‐proNrCAM) levels remained unaltered (Fig EV2C), in agreement with a previous study (Suzuki *et al*, 2012). This suggests that small changes in mADAM10 levels affect APP shedding more strongly than NrCAM shedding. Together with prior results, using NrCAM mutants carrying the mutated furin cleavage site (Suzuki *et al*, 2012), we conclude that under non‐inhibited conditions, NrCAM first undergoes cleavage by furin and subsequently by ADAM10.

#### NrCAM is a γ‐secretase substrate

**Figure 2:**
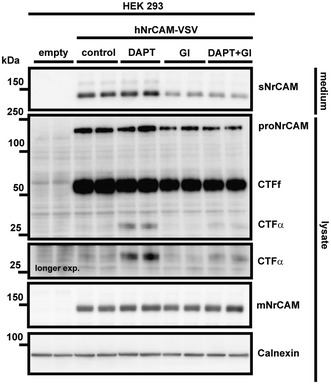
NrCAM is a γ‐secretase substrate HEK 293 cells were transfected with a C‐terminally VSV‐tagged NrCAM construct or empty vector. Cells were then treated with DAPT (1 μM), GI254023x (5 μM), either substances or solvent for 24 h. The C‐terminal NrCAM fragment was detected with an antibody against the VSV‐tag. Representative Western blots are shown.Source data are available online for this figure.

After ADAM10 cleavage, the remaining membrane‐bound C‐terminal fragments of several type I membrane proteins, such as APP and L1CAM, are further processed by the γ‐secretase complex, an intramembrane protease (Lichtenthaler *et al*, 2011). As a result, a short N‐terminal peptide—comprising the short remaining ectodomain and half of the transmembrane domain—is typically secreted from cells, whereas the intracellular domain (ICD) is released into the cytosol, where it is mostly rapidly degraded. Inhibition of γ‐secretase, for example, with the established inhibitor DAPT (Dovey *et al*, 2001), blocks ICD generation and leads to increased CTF levels (for a schematic, see Fig 1A), which is used as a read‐out to monitor whether a membrane protein is a substrate for γ‐secretase. In order to examine whether the type I membrane protein NrCAM is also cleaved by γ‐secretase, we analyzed HEK293 cells that had been transiently transfected with a C‐terminally VSV‐tagged human NrCAM construct. As expected, NrCAM maturation and sNrCAM shedding by ADAM10 were not affected by inhibition of γ‐secretase with DAPT (Fig 2). In contrast, DAPT led to the appearance of a C‐terminal NrCAM fragment (termed CTFα) with a molecular weight of around 27 kDa and was not seen upon additional inhibition of ADAM10 with GI254023X (Fig 2) which is in agreement with the fragment ranging from the ADAM10 cleavage site to the C‐terminus of NrCAM. As a control, the fragment was barely seen in control‐treated cells and not seen in control‐transfected cells not expressing NrCAM. Taken together, these findings demonstrate that NrCAM is not only a substrate for ADAM10, but also for γ‐secretase.

### ADAM10 controls surface levels of NrCAM and neurite outgrowth

**Figure 3:**
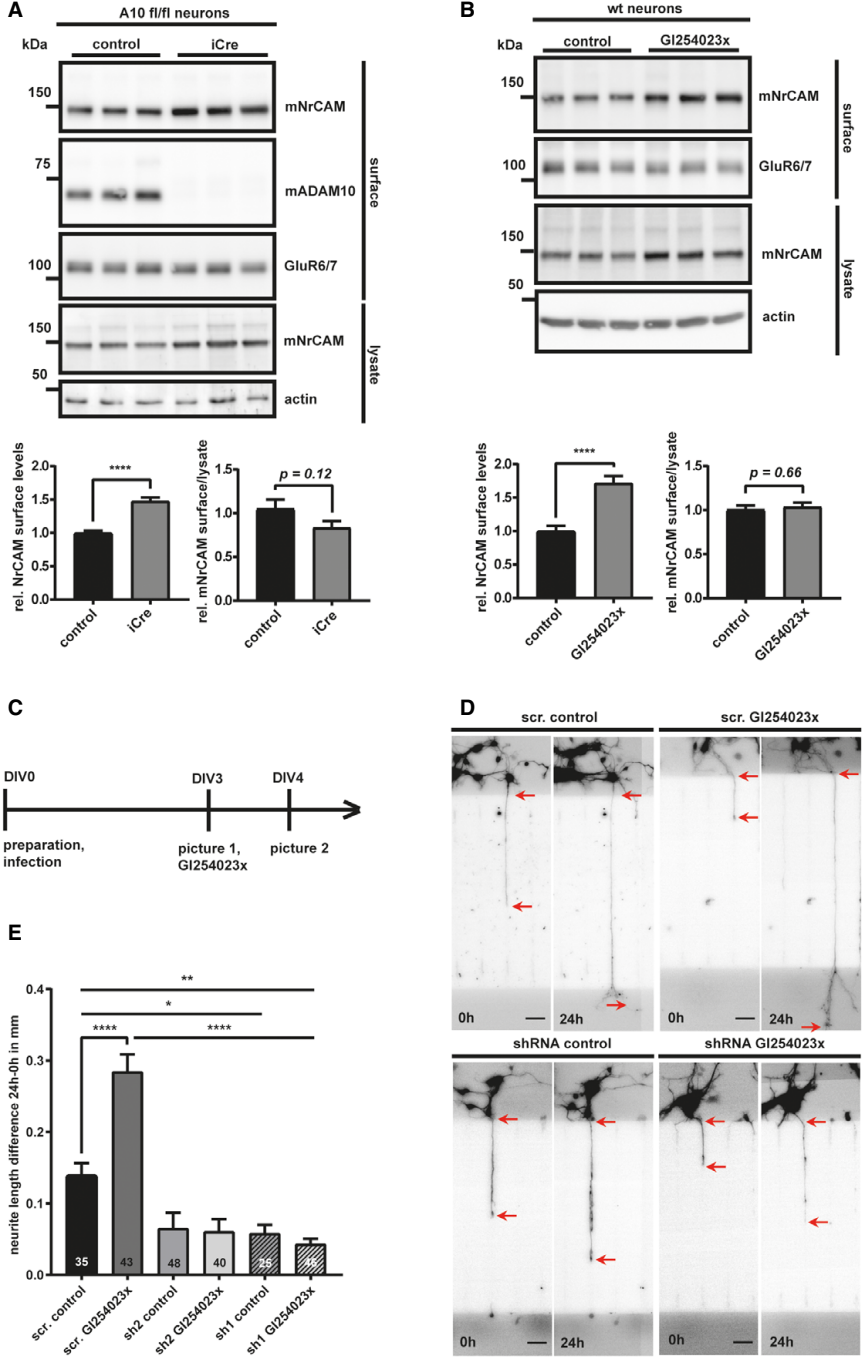
ADAM10 is controlling surface expression of NrCAM and NrCAM‐mediated neurite outgrowth AADAM10fl/fl neurons were treated with an iCre (to induce an ADAM10 KO), or a control lentivirus at DIV2. At DIV7, cell surface proteins were labeled with biotin and enriched by streptavidin pull‐down. The biotinylated proteins were detected by immunoblotting. Total lysates were analyzed to compare mNrCAM's surface/lysate levels. Densitometric quantifications of the Western blots are shown. Two‐sided Student's *t*‐test (****P < 0.0001, *n* = 7). Given are mean ± the standard error of the mean. The mean levels of solvent‐treated cells were set to 1.BPrimary murine neurons were treated with GI254023x (5 μM) or solvent, for 48 h. The cell surface proteins were enriched like in (A). Densitometric quantifications of the Western blots are shown. Two‐sided Student's *t*‐test (****P < 0.0001, *n* = 11). Given are mean ± the standard error of the mean. The mean levels of solvent‐treated cells were set to 1. Representative Western blots are shown.CWorkflow of the neurite outgrowth assay.DRepresentative pictures of single neurites at timepoint 0 h (DIV3) and 24 h (DIV4). Neurons were infected 4 h after plating with lentiviruses encoding GFP (to visualize the neurons) together with shRNA expression cassettes (sh1 and sh2) targeting either NrCAM or carrying a scrambled (scr) control construct. Images of neurites were taken at 3 days *in vitro* (DIV3) and 24 h later at DIV4. In order to study the effect of ADAM10 on neurite outgrowth, neurons were treated with the ADAM10 inhibitor GI254023x, or vehicle (control), at DIV3, after taking the first pictures with an epifluorescent microscope. The differences in neurite length were calculated as absolute values (neurite length at 24 h minus neurite length of 0 h) for individual neurites passing through the middle channels of the chambers. Only neurites that had already entered the main channel at 0 h and had not yet left those channels at 0 h were considered. The red arrows indicate the start and the end of the respective length measurements. The scale bar indicates 40 μm.EQuantification and statistical analysis of the neurite outgrowth assay shown in (D). Scr. = scrambled; sh 1 and 2 = shRNA1 and 2. One‐way ANOVA with *post hoc* Dunnett's test. Given are mean ± the standard error of the mean (*P < 0.05; **P < 0.01; ****P < 0.0001, *n*‐numbers for each condition are shown in the graph). Source data are available online for this figure.

**Figure EV3:**
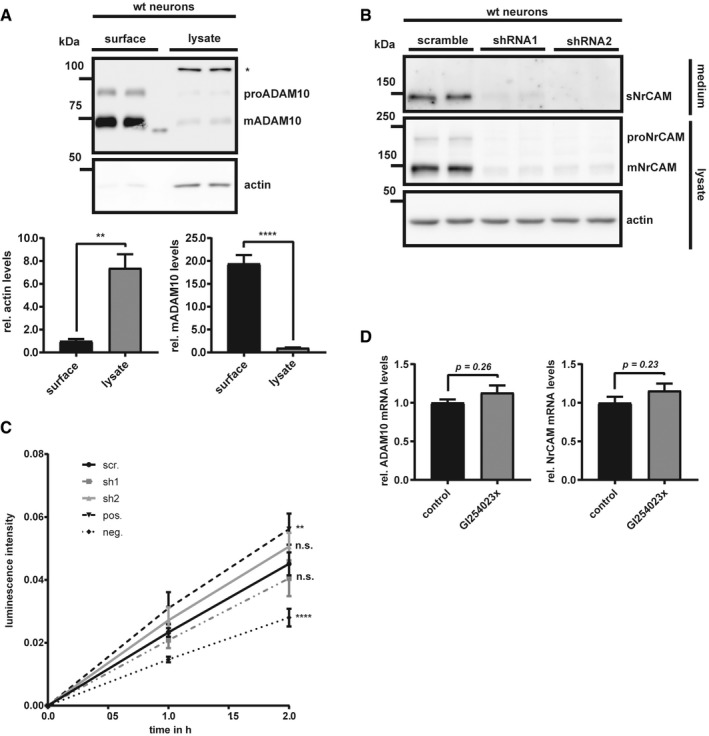
Surface biotinylation efficiency and knock‐down of NrCAM using shRNA‐containing lentiviruses ATo test the efficacy of our biotinylation assay, 100 μg of total protein was used for streptavidin pull‐down, run next to 10 μg of total lysate, and were compared for the respective abundance of β‐actin and ADAM10. Densitometric quantifications of the Western blots are shown. Two‐sided Student's *t*‐test (**P < 0.01; ****P < 0.0001, *n* = 4). Given are mean ± the standard error of the mean. * indicates an unspecific band. Surface and lysate samples are separated by a marker lane.BWt neurons were infected with NrCAM shRNA‐containing lentiviruses (shRNA1 or shRNA2; 1:1,000), or a scrambled control construct (1:1,000). At DIV4, the cells were lysed and the conditioned media were collected.CCell viabilities after infection with the respective viruses (scr., sh1 and sh2) were compared to a positive (no toxic effect) and negative control (high toxicity) with Cell Counting Kit 8 (Sigma). The luminescence signals were measured with a microplate reader. Both shRNAs did not show higher toxicity than the scrambled control virus. Pos. = positive survival control (no treatment); neg. = negative survival control (cells treated with NaOH). One‐way ANOVA with *post hoc* Dunnett's test (^n.s^ *P* > 0.05; **P < 0.01; ****P < 0.0001, *n* = 8). *P*‐values were calculated by comparing the respective treatments to the treatment with the scr. shRNA. Shown are mean and SEM. Representative Western blots are shown.DqPCRs for the ADAM10 and NrCAM mRNAs from wt neurons treated with GI254023x or solvent for 48 h (preparation and treatment of the cells were performed like in Fig 3B). Two‐sided Student's *t*‐test (*n* = 5–6). Given are mean ± the standard error of the mean. Source data are available online for this figure.

Next, we tested whether loss of ADAM10 cleavage increased surface NrCAM levels and altered neurite outgrowth *in vitro*, which is one of the established functions of NrCAM as a neuronal surface co/receptor (Morales *et al*, 1993; Sakurai *et al*, 1997; Falk *et al*, 2004; Zelina *et al*, 2005; Nawabi *et al*, 2010; Torre *et al*, 2010; Demyanenko *et al*, 2011, 2014; Kuwajima *et al*, 2012; Dai *et al*, 2013). Indeed, surface mNrCAM levels were increased by about 50% in conditional ADAM10 knock‐out neurons compared to control‐transduced neurons, as established by cell surface biotinylation (Figs 3A and EV3A). The same result was obtained in wild‐type neurons treated with the ADAM10‐preferring inhibitor GI254023X (Fig 3B). Importantly, ADAM10 inhibition did not alter mRNA levels of either ADAM10 or NrCAM (Fig EV3D). As a control, levels of the unrelated kainate receptor GluR6/7, which is not a substrate of ADAM10 (Kuhn *et al*, 2016), were used as negative control in the surface biotinylation assay and did not show altered surface levels upon inhibition or knock‐out of ADAM10 (Fig 3B). Taken together, these results reveal that ADAM10 controls cell surface abundance of NrCAM in primary cortical neurons.

To test whether this affects neurite outgrowth in an NrCAM‐dependent manner, neurite growth of single cortical neurons was traced in microfluidic chambers, where neurite and soma compartment were separated (for an overview of the time line, see Fig 3C). Neurons were infected 4 h after plating with a lentivirus encoding GFP (to visualize the neurons) together with two different shRNA expression cassettes targeting either NrCAM (Fig EV3B and C) or carrying a scrambled control construct. ADAM10 inhibition by GI254023X doubled the length of the neurite outgrowth within 24 h. Knock‐down of NrCAM (with shRNAs 1 and 2) decreased neurite outgrowth compared to the control‐treated cells by about 50% (Fig 3D and E). Addition of the ADAM10‐preferring inhibitor GI254023X was not able to rescue the reduced neurite outgrowth. Taken together, the results reveal that ADAM10 controls neuronal surface levels of NrCAM and neurite outgrowth in an NrCAM‐dependent manner.

### Acitretin increases APP but not NrCAM shedding in primary neurons and AD patients

**Figure 4:**
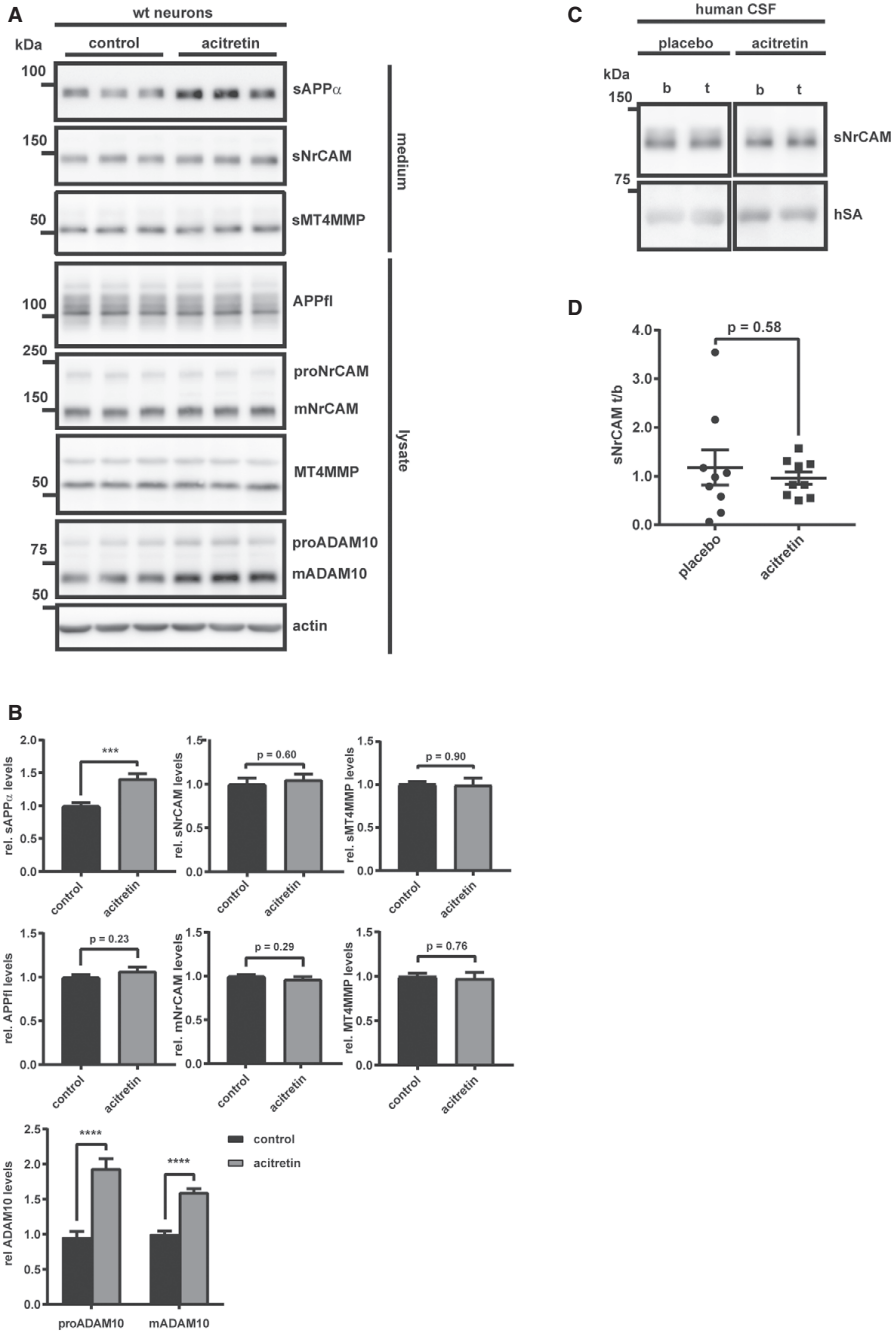
Acitretin increases APP but not NrCAM shedding in primary neurons and AD patients AWt neurons were kept in culture until DIV5; then, the cells were treated with acitretin (4 μM) or vehicle control for 48 h, as described earlier (Tippmann *et al*, 2009).BDensitometric quantifications of the Western blots are shown (***P < 0.001; ****P < 0. 0001, two‐sided Student's *t*‐test, *n* = 9–20). The mean levels of solvent‐treated cells were set to 1.CDetection of sNrCAM and human serum albumin (hSA) in AD patients CSF that had been treated with acitretin (30 mg/day) or placebo for 30 days (*n* = 9) (Endres *et al*, 2014). b = baseline; t = treatment.DDensitometric quantification of the Western blots in (C). sNrCAM/hSA ratios were calculated, and then, the treatment‐to‐baseline (t/b) ratios for every patient were calculated. Two‐sided Student's *t*‐test (*n* = 9).Data information: Given are mean ± the standard error of the mean. Representative Western blots are shown.Source data are available online for this figure.

**Figure EV4:**
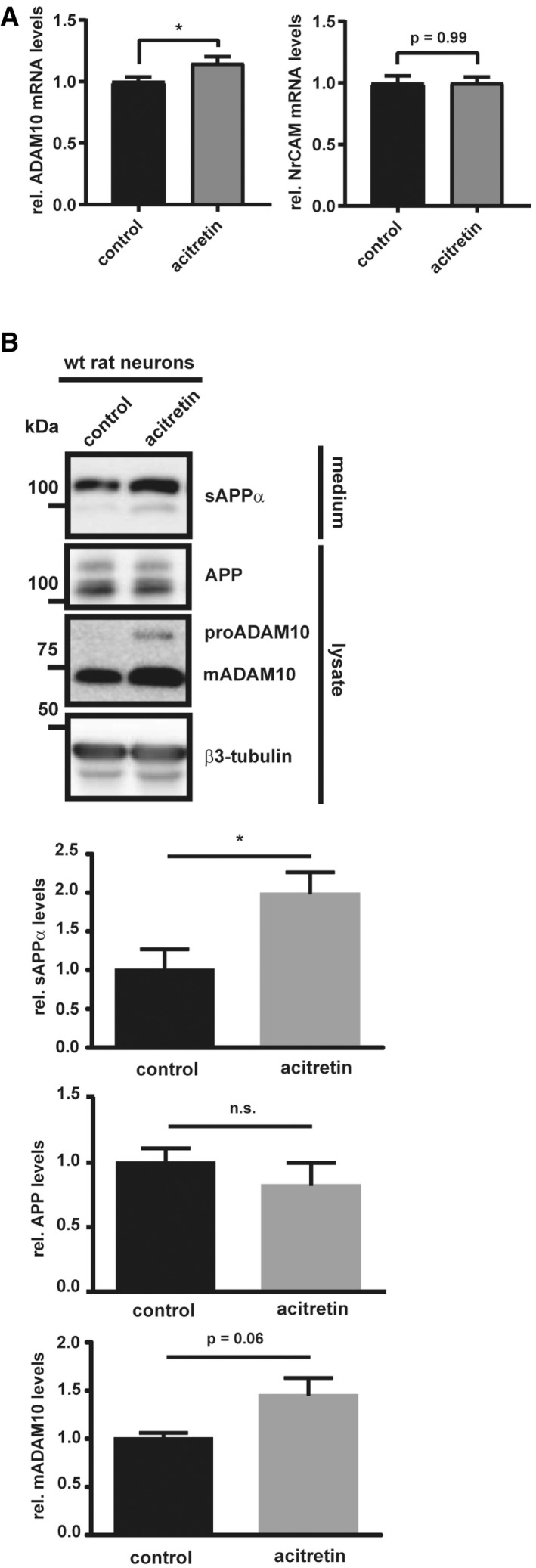
Acitretin increases ADAM10 transcription in murine and ADAM10 and sAPPa levels in aged rat neurons AqPCRs for the ADAM10 and NrCAM mRNAs from wt neurons (DIV7) treated with acitretin (4 μM) or solvent for 48 h (preparation and treatment of the cells were performed like in Fig 4A). Two‐sided Student's *t*‐test (*P < 0.05, *n* = 15). Given are mean ± the standard error of the mean.BCortical neurons were prepared from Wistar rat (embryonic day E18) and cultured for 21 days. Acitretin (2 μM) or solvent was added at DIV19. Two‐sided Student's *t*‐test (^n.s^ *P* > 0.05; *P < 0.05, *n* = 4). Given are mean ± the standard error of the mean. Representative Western blots are shown. Source data are available online for this figure.

A mild stimulation of APP shedding by ADAM10 is considered as a possible treatment strategy for AD and has been successfully tested in a recent phase II clinical trial, where acitretin indeed mildly increased sAPPα levels by 25% (Endres *et al*, 2014). Effects on other ADAM10 substrates or on cognitive measures of the treated patients were not observed. Major mechanism‐based side effects were not seen in that study. However, they remain a concern for future, larger clinical studies given the broad spectrum of neuronal ADAM10 substrates (Kuhn *et al*, 2016), including NrCAM and the role of its cleavage by ADAM10 in neurite outgrowth as demonstrated above. Thus, sNrCAM may potentially serve as a biomarker for drug responses in acitretin trials. While our experiments so far demonstrated that sNrCAM is a suitable substrate marker to monitor decreases in ADAM10 activity, we now tested whether NrCAM may also be used to monitor increases in ADAM10 activity and, thus, may potentially serve as a biomarker for drug responses in acitretin trials. To this aim, we treated primary neurons with or without acitretin. As a control, we first monitored levels of sAPPα. As expected and in agreement with previous studies (Tippmann *et al*, 2009; Endres *et al*, 2014), acitretin increased sAPPα levels in the conditioned medium of murine and rat neurons and ADAM10 protein levels in the neuronal lysate (Figs 4A and B, and EV4A and B). ADAM10 was also increased at the mRNA level (Fig EV4A), as determined in the murine neurons and in line with previous studies (Tippmann *et al*, 2009; Endres *et al*, 2014). Surprisingly, however, acitretin did not increase sNrCAM levels (Fig 4A and B). Moreover, NrCAM mRNA levels, as well as full‐length NrCAM protein (proNrCAM and mNrCAM), were also not affected by acitretin treatment (Figs 4A and B, and EV4A). Moreover, shedding of sMT4‐MMP, another ADAM10 substrate (Kuhn *et al*, 2016), was also not altered upon acitretin treatment, suggesting a relatively specific enhancing effect on APP shedding. To corroborate these findings *in vivo*, we analyzed human CSF samples from the recent acitretin phase II clinical study. sNrCAM levels were compared by immunoblots in individual patients before and after treatment (Fig 4C and D). Albumin, which is endogenously present in human CSF, was used as a loading control. Similar to the primary neuron experiment *in vitro*, acitretin treatment did not increase sNrCAM levels in the patients’ CSF (Fig 4D), while sAPPα was increased by 1.25‐fold in the same samples, as reported previously (Endres *et al*, 2014). This unexpected finding demonstrates that it is possible to activate the ADAM10‐mediated cleavage of APP without interfering with the shedding of NrCAM, another ADAM10 substrate.

### NrCAM shedding can be stimulated with other ADAM10 cleavage activators

**Figure 5:**
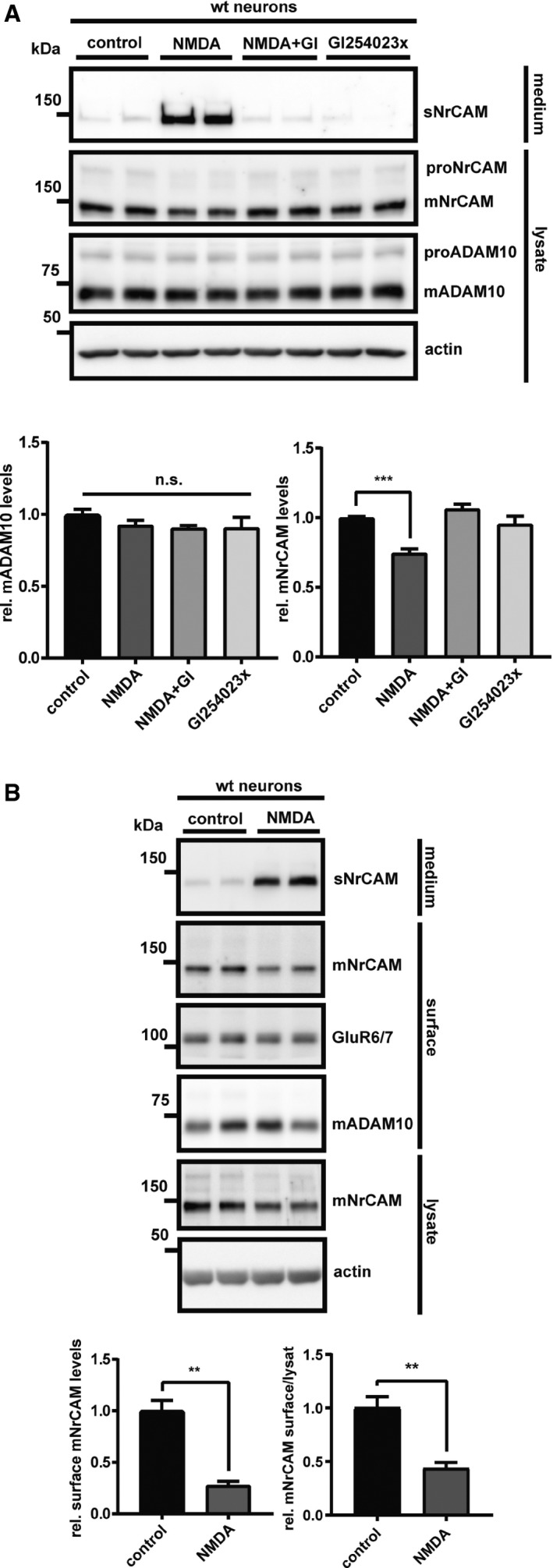
Neuronal stimulation by NMDA increases ADAM10‐mediated NrCAM shedding ATo ensure proper synapse formation, primary neurons were kept in culture for 10 days prior to the treatment (Wan *et al*, 2012). Then, cells were treated with NMDA (50 μM), NMDA (50 μM) and GI254023x (5 μM), GI254023x (5 μM) alone or vehicle for 30 min. Total cellular mADAM10 levels remained unchanged by the treatment, which is in line with NMDA altering mainly the intracellular localization of ADAM10 by driving the protein to the synaptic membranes (Marcello *et al*, 2007). Densitometric quantifications of the Western blots are shown. One‐way ANOVA with *post hoc* Dunnett's test (***P < 0.001, *n* = 7). Given are mean ± the standard error of the mean. The mean levels of solvent‐treated cells were set to 1. The membrane was reprobed with the different indicated antibodies.BWt neurons were kept in culture until DIV10; then, the cells were treated with NMDA (50 μM), or vehicle for 30 min. Cell surface proteins were labeled with biotin and enriched by streptavidin pull‐down. The biotinylated proteins were detected by immunoblotting. Total lysates were analyzed to compare mNrCAMs surface/lysate levels. Densitometric quantifications of the Western blots are shown. Two‐sided Student's *t*‐test (**P < 0.01, *n* = 6). Given are mean ± the standard error of the mean. The mean levels of solvent‐treated cells were set to 1. Representative Western blots are shown. Source data are available online for this figure.

**Figure EV5:**
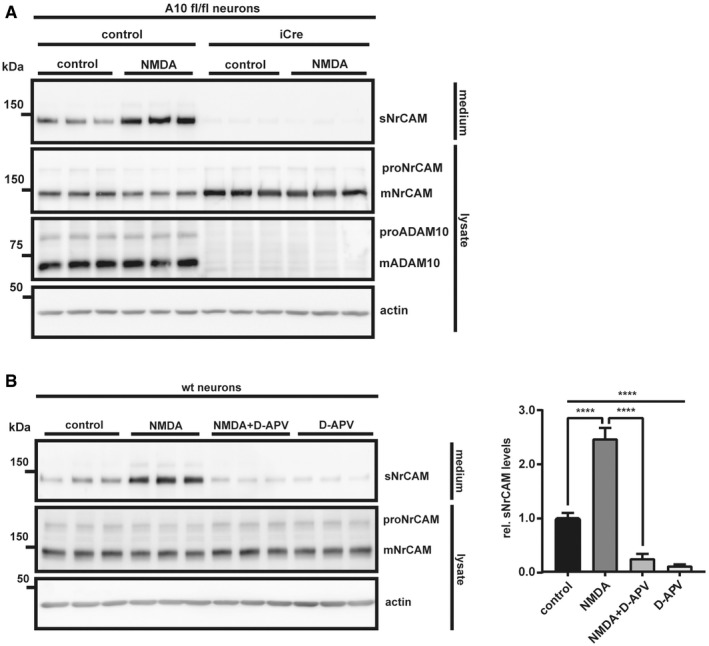
Neuronal stimulation by NMDA increases ADAM10‐mediated NrCAM shedding ATo genetically validate our findings, ADAM10fl/fl neurons were treated with an iCre, or a control lentivirus at DIV2, to knock out ADAM10. The cells were kept in culture until DIV10; then, the neurons were treated with NMDA (50 μM) or vehicle for 30 min (Wan *et al*, 2012).BWt neurons were cultured like in (Fig 5A). At DIV10, the cells were pretreated with the NMDA receptor antagonist D‐APV (100 μM) or vehicle for 30 min; then, the neurons were treated with NMDA (50 μM) or vehicle for 30 min. Densitometric quantifications of the Western blots are shown. One‐way ANOVA with *post hoc* Dunnett's test (****P < 0. 0001, *n* = 6). Shown are the mean and SEM. Representative Western blots are shown. Source data are available online for this figure.

To rule out the possibility that acitretin did not increase NrCAM shedding, simply because NrCAM shedding is already at the maximum and cannot be further increased, we did a further control experiment to demonstrate that NrCAM shedding can be activated with a suitable stimulus. We chose NMDA, which is known to increase ADAM10's synaptic localization and activity (Maretzky *et al*, 2005; Marcello *et al*, 2007; Wan *et al*, 2012) and consequently ADAM10 activity toward synaptic substrates, such as APP, Nectin‐1, or Neuroligin‐1 (Marcello *et al*, 2007; Kim *et al*, 2010; Suzuki *et al*, 2012). Primary cortical neurons were treated with NMDA, GI254023X, or both for 30 min, which resulted in a strong increase in sNrCAM levels and a mild concomitant decrease of mNrCAM in the lysate (Fig 5A). Importantly, this effect was prevented when the neurons were additionally treated with GI254023X, demonstrating that NMDA activated the cleavage of NrCAM through ADAM10 (Fig 5A). The same result was obtained using ADAM10 knock‐out neurons (Fig EV5A). As a control, the NMDA‐induced increase in NrCAM cleavage was blocked with the specific NMDA receptor antagonist D‐APV (Fig EV5B).

Finally, we asked whether the NMDA‐activated ADAM10 cleavage of NrCAM would control surface levels of NrCAM in a manner similar to Fig 3, where we found that also constitutive ADAM10 cleavage is a mechanism to regulate NrCAM surface levels. To this aim, we performed cell surface biotinylation of cortical neurons, stimulated with NMDA for 30 min. Treatment with NMDA significantly decreased the surface expression of mNrCAM by nearly 75% (Fig 5B), while only mildly reducing mNrCAM levels in the total lysate. This suggests that the short‐term treatment with NMDA predominantly induces sNrCAM shedding at the cell surface.

We conclude that ADAM10 is not only the constitutive, but also a stimulated NrCAM sheddase and that ADAM10 controls cell surface levels of NrCAM both under basal, non‐stimulated conditions and upon neuronal stimulation with NMDA.

### Acitretin selectively releases substrates and non‐substrates into the CSF of Alzheimer patients

**Figure 6:**
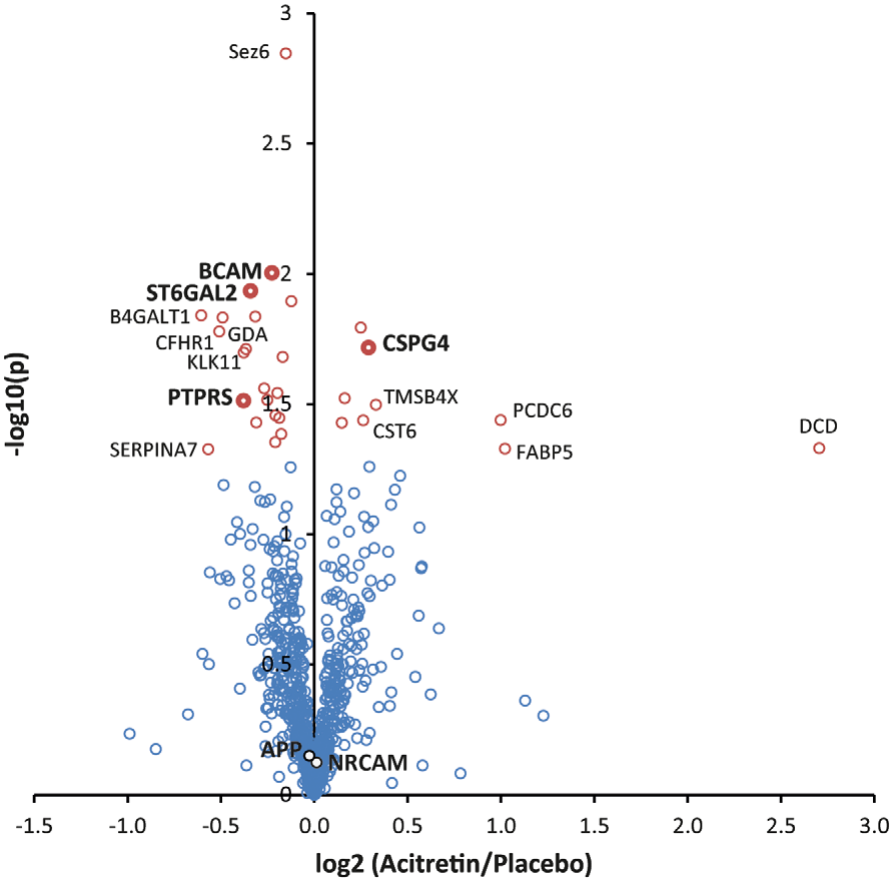
Mass spectrometric analysis of acitretin CSF samples Volcano plot of the proteomic analysis of CSF from AD patients treated with acitretin or placebo for 30 days. Every circle represents one protein. The log10‐transformed *t*‐test *P*‐values (again treatment/baseline for every patient) are plotted against the log2‐transformed label‐free quantification intensity ratios of acitretin and placebo CSF. Proteins with a *P*‐value of less than 0.05 are labeled in red. Proteins that are additionally changed by more than 20% compared to placebo are labeled with their names. ADAM10 substrates are indicated in bold writing. In total, more than 50 ADAM10 substrates were identified, but only the significantly altered substrates as well as NrCAM and APP are indicated.

The experiments above revealed that acitretin selectively activated ADAM10 cleavage of APP, but not of NrCAM. If acitretin would also spare other ADAM10 substrates, this would be a major advantage for AD treatment as it may reduce potential side effects resulting from increased cleavage of other known ADAM10 substrates. Thus, we systematically tested the effect of acitretin on the CSF proteome of AD patients. We obtained 18 CSF samples from the recent clinical phase II trial (Endres *et al*, 2014) that were analyzed by immunoblot for NrCAM shedding in Fig 4C. Protein intensities after treatment (t) with either acitretin or placebo were divided by the corresponding baseline (b) protein intensities before treatment (t/b ratio) for every patient. To determine acitretin‐dependent effects, the baseline normalized protein intensities (t/b ratios) of the acitretin‐treated group were compared to the placebo group. The results are displayed in a volcano plot in which the minus log10‐transformed *P*‐value is plotted against the log2‐transformed average ratio of the acitretin and placebo group (Fig 6). Given the overall mild changes, we did not apply multiple hypothesis corrections, for example, using a Bonferroni test. Instead, proteins were considered as potential hits if their significance *P*‐value was < 0.05 using a Student's *t*‐test and had levels that were up‐ or down‐regulated in the acitretin group by more than 20% compared to the controls and thus, in a similar range as sAPPα, which was reported to be increased in the same samples by 25% (Endres *et al*, 2014). This yielded 8 proteins (SERPINA7, PTPRS, CFHR1, B4GALT1, ST6GAL2, BCAM, GDA, and KLK11) being down‐regulated and 6 proteins (DCD, FABP5, PDCD6, TMSB4X, CSPG4, and CST6) being up‐regulated (Fig 6, Table 1). Surprisingly, only one previously known ADAM10 substrate, CSPG4/NG2, which is expressed in oligodendrocyte precursor cells (Sakry *et al*, 2014), was found among the proteins with increased levels (Fig 6), while over 50 known ADAM10 substrates (Dataset EV1) did not show significant changes, including NrCAM, thus confirming the Western blot results from Fig 4C and D. Three additional ADAM10 substrates, ST6GAL2 (Kuhn *et al*, 2016), BCAM (Cai *et al*, 2016), and PTPRS (Kuhn *et al*, 2016), even showed a lower abundance than in the placebo group (see also Table 1), clearly showing that acitretin did not increase the shedding of all ADAM10 substrates. Additionally, it is important to note that the extent of the changes was mild for the one substrate with increased (22%) and the three substrates with decreased (up to 40%) ADAM10 cleavage products. While acitretin increased sAPPα in the CSF (Endres *et al*, 2014) and in our neuronal experiments (Fig 4A), total levels of sAPP were not altered in the CSF, so that APP was not detected as a hit in the proteomic analysis (Fig 6). This result was expected because total sAPP does not only comprise sAPPα, but also all other sAPP forms generated by other proteases, including BACE1 and δ‐secretase. These individual sAPP species can well be detected by immunoblots using cleavage site‐specific antibodies, but not by mass spectrometry, where all tryptic peptides of APP are used for quantification. We consider the possibility that some of the additional ADAM10 substrates may have—similar to APP—mildly increased ADAM10 cleavage and a compensating reduction in cleavage by other proteases, such that total cleavage was not altered with acitretin in the proteomic study.

**Table 1:** Significantly changed proteins in AD patients treated with acitretin

Protein name	Gene name	(Aci./Pla.)	*P*‐value	Reference ADAM10 substrate
Decrease				
**Basal cell adhesion molecule**	**BCAM**	0.79	**0.0116**	**Cai ** ***et al*** ** (** 2016)
Beta‐1,4‐galactosyltransferase 1	B4GALT1	0.66	0.0144	
**Beta‐galactoside alpha‐2,6‐sialyltransferase 2**	**ST6GAL2**	0.71	**0.0147**	**Kuhn ** ***et al*** ** (** 2016)
Complement factor H‐related protein 1	CFHR1	0.70	0.0166	
Guanine deaminase	GDA	0.78	0.0194	
Kallikrein‐11	KLK11	0.77	0.0200	
**Receptor‐type tyrosine‐protein phosphatase S**	**PTPRS**	0.77	**0.0306**	**Kuhn ** ***et al*** ** (** 2016)
Thyroxine‐binding globulin	SERPINA7	0.67	0.0470	
Increase				
**Chondroitin sulfate proteoglycan 4**	**CSPG4**	1.22	**0.0191**	**Sakry ** ***et al*** ** (** 2014)
Thymosin beta‐4	TMSB4X	1.26	0.0318	
Programmed cell death protein 6	PDCD6	2.00	0.0364	
Cystatin‐M	CST6	1.20	0.0365	
Dermcidin; Survival‐promoting peptide	DCD	6.52	0.0467	
Fatty acid‐binding protein, epidermal	FABP5	2.03	0.0469	

This table contains all proteins with a *t*‐test significance *P*‐value of < 0.05 (not corrected for multiple testing) and a change of the protein level of more than 20% compared to placebo‐treated controls. ADAM10 substrates are marked in bold.

Besides the few ADAM10 substrates, 10 proteins had increased (DCD, FABP5, PDCD6, TMSB4X, and CST6) or reduced (SERPINA7, CFHR1, B4GALT1, GDA, and KLK11) CSF levels. Because they are not membrane proteins with the exception of B4GALT1, they are unlikely to be direct ADAM10 substrates. Instead, they may be changed as an indirect effect of ADAM10 activation or of the drug acitretin itself. Interestingly, B4GALT1 is a known substrate for the protease SPPL3 (Kuhn *et al*, 2015), so that the reduced CSF levels also appear as an indirect effect of the acitretin treatment. Taken together, the proteomic analysis of the clinical CSF samples reveals that acitretin only mildly affected the CSF protein composition of AD patients and demonstrates that the acitretin‐induced ADAM10 activation is presumably moderate *in vivo*, as only few ADAM10 substrates showed altered protein levels in CSF.

### TSPAN15 has opposite effects on ADAM10‐mediated shedding of NrCAM and APP

**Figure 7:**
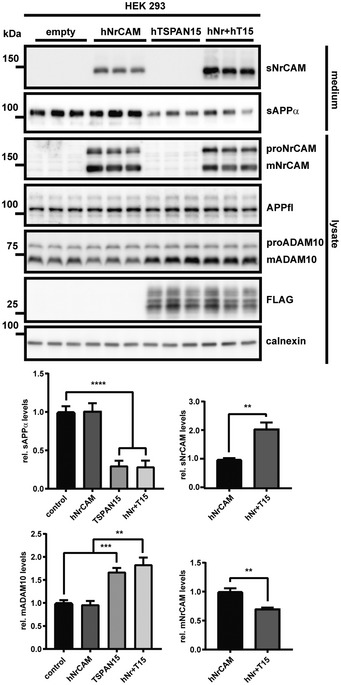
TSPAN15 has opposite effects on ADAM10‐mediated NrCAM and APP shedding HEK293 cells were transiently transfected with hNrCAM, FLAG‐tagged hTSPAN15, either plasmids or the empty vector. Detection of sAPPα and sNrCAM in the conditioned media and ADAM10 in the cell lysate. Densitometric quantifications of the Western blots are shown (**P < 0.01; ***P < 0.001; ****P < 0.0001; two‐sided Student's *t*‐test was used for sNrCAM and mNrCAM and one‐way ANOVA with *post hoc* Dunnett's test for sAPPα and mADAM10, *n* = 6). Given are mean ± the standard error of the mean. The mean levels of solvent‐treated cells were set to 1. Representative Western blots are shown.Source data are available online for this figure.

We next wished to provide a better understanding of the unexpected finding that the general ADAM10 activator acitretin only stimulates shedding of APP and CSPG4/NG2 in the patient CSF, but not of NrCAM and many other ADAM10 substrates. ADAM10 binds to six different membrane proteins of the tetraspanin (TSPAN) family and it has been suggested that they control the substrate specificity of ADAM10. To test whether a tetraspanin may indeed have differential effects on APP and NrCAM shedding, we used TSPAN15 (Matthews *et al*, 2017), which is the best studied family member among the ADAM10 interactors (Haining *et al*, 2012; Jouannet *et al*, 2016; Noy *et al*, 2016; Seipold *et al*, 2018). TSPAN15 or empty control vector was co‐transfected with NrCAM into HEK293 cells, which only express low levels of endogenous TSPAN15. Similar to acitretin, TSPAN15 increased mADAM10 levels by about 50% (Fig 7), which is in line with previous reports (Haining *et al*, 2012; Noy *et al*, 2016). Interestingly, TSPAN15 significantly increased the shedding of sNrCAM (twofold), while it decreased the release of sAPPα (threefold), showing that TSPAN15 does indeed differentially regulate NrCAM and APP cleavage. Furthermore, these results show that an increase in mADAM10 does not necessarily increase the shedding of all of its substrates.

## Discussion

Our study establishes the cell adhesion protein NrCAM as a physiological substrate for the α‐secretase ADAM10 and reveals a role for ADAM10 in neurite outgrowth through NrCAM cleavage. Furthermore, our study demonstrates that soluble, ADAM10‐cleaved sNrCAM is an excellent marker in AD patients for developing and testing drugs that selectively activate cleavage of APP over other ADAM10 substrates. Finally, whole proteome analysis of patient samples revealed that acitretin in a clinical trial surprisingly activates ADAM10 in a substrate‐selective manner and, thus, may be safer in patients than expected.

ADAM10 is a drug target in AD, where an activation of the ADAM10 cleavage of APP is therapeutically desired in order to reduce generation of the neurotoxic Aβ peptide (Postina, 2012; Marcello *et al*, 2017). Importantly, a mild increase of only 30% of mature ADAM10 levels in mouse brains was sufficient to lower amyloid β levels and prevented amyloid pathology in an AD mouse model (Postina *et al*, 2004). Yet, ADAM10 has numerous additional substrates in the brain and in other organs (Crawford *et al*, 2009; Saftig & Lichtenthaler, 2015; Kuhn *et al*, 2016), which may cause mechanism‐based side effects upon therapeutic ADAM10 activation. The second generation retinoid acitretin, which is in clinical use to treat psoriasis, mildly increases ADAM10 levels *in vitro* by 1.3‐fold (Tippmann *et al*, 2009) and sAPPα levels in human CSF by 25% (Endres *et al*, 2014) and, thus, was expected to also increase CSF levels of the cleaved ectodomain of other ADAM10 substrates in the brain. Surprisingly, however, this was not the case. Only one previously known ADAM10 substrate (CSPG4/NG2) (Sakry *et al*, 2014) had mildly increased CSF levels (22%)—similar to sAPPα—, while more than 50 previously identified ADAM10 substrates or substrate candidates, including sNrCAM, did not show significantly altered levels in CSF. Interestingly, three known ADAM10 substrates (BCAM, ST6Gal2, and PTPRS) even had slightly reduced CSF levels. CSPG4/NG2 (Sakry *et al*, 2014), which was the only up‐regulated substrate apart from sAPPα (Endres *et al*, 2014), is mainly expressed in oligodendrocyte precursor cells. CSPG4 is an important regulator for adaptive processes following brain damage (Schafer & Tegeder, 2018) and is able to promote neurite regrowth into glial scar tissue (Vadivelu *et al*, 2015).

ADAM10 cleaves membrane proteins, but several of the affected proteins were not membrane proteins and, thus, their levels are unlikely to be changed because of an increased, direct cleavage by ADAM10. Yet, their changes may be secondary consequences of ADAM10 activation, as ADAM10 activity is required for different signaling pathways, including Notch signaling (Pan & Rubin, 1997). This assumption is in agreement with a previous study demonstrating that mild (30%) neuronal overexpression of ADAM10 in mouse brain altered expression of up to 355 genes, although their RNA levels were mostly only moderately up‐ or down‐regulated by up to 40% (Prinzen *et al*, 2009). Another mechanism for changes in protein expression may be ADAM10‐independent effects of acitretin. For example, FABP5, which had increased CSF levels upon acitretin treatment, is an all‐trans retinoic acid (atRA) transport protein (Hohoff *et al*, 1999; Smathers & Petersen, 2011). Its increased levels are likely a direct consequence of acitretin's function in indirectly elevating cellular atRA levels (Tippmann *et al*, 2009), because retinoic acid is known to increase FABP5 expression (Yu *et al*, 2012). The strongest changes were obtained for PDCD6 and DCD. PDCD6 participates in T‐cell receptor‐, Fas‐, and glucocorticoid‐induced programmed cell death, while DCD is an antimicrobial protein that is mainly expressed in sweat glands, whereas its N‐terminal fragment promotes neural cell survival under conditions of oxidative stress (Jung *et al*, 2001; Tarabykina *et al*, 2004; Dash‐Wagh *et al*, 2011; Burian & Schittek, 2015). Whether the function of both proteins is altered in acitretin‐treated patients remains to be investigated. Potentially, DCD could be used to monitor increased ADAM10 activity in patients by simply testing their sweat.

The selective CSF increase of the cleavage products of only two ADAM10 substrates (sAPPα, sCSPG4/sNG2) upon acitretin treatment in patients is unexpected, but our study offers insights into the possibly underlying molecular mechanisms. While NrCAM (this study) and APP (Jorissen *et al*, 2010; Kuhn *et al*, 2010) both require ADAM10 for their cleavage under constitutive, non‐stimulated conditions, our study demonstrates that activation of NrCAM and APP shedding beyond the constitutive level can occur through different molecular mechanisms. For example, PMA stimulated shedding of APP, but not NrCAM in primary murine neurons (Fig EV1C and D). Conversely, overexpression of the tetraspanin TSPAN15 increased NrCAM shedding, but even reduced APP shedding in HEK293 cells. This finding is particularly remarkable, as expression of TSPAN15, a known ADAM10 binding partner (Haining *et al*, 2012; Prox *et al*, 2012; Noy *et al*, 2016), increased the levels of mature ADAM10, similar to what is known for acitretin treatment (Tippmann *et al*, 2009). Thus, an increase in mature ADAM10 cannot be taken as evidence for increased ADAM10 activity toward all of its substrates. In fact, it has been suggested that the six members of the C8 subgroup of the tetraspanin family of four‐pass transmembrane proteins, including TSPAN15, do not only bind to ADAM10, but also to its substrates and thus control the substrate specificity of ADAM10 (Matthews *et al*, 2017). As a result, ADAM10 may be present in six different complexes, with potentially each of them enabling cleavage of different sets of ADAM10 substrates (Jouannet *et al*, 2016; Matthews *et al*, 2017). This assumption is consistent with our finding that TSPAN15 expression had opposite effects on the shedding of NrCAM and APP. Likewise, a recent study demonstrated that TSPAN15 deficiency in mice reduced ADAM10 maturation and selectively reduced ADAM10 cleavage of N‐cadherin and the prion protein, whereas APP cleavage by ADAM10 was not affected (Seipold *et al*, 2018). Whether TSPAN15 shows an altered expression in the AD patients that were treated with acitretin is not known, as the brains of these individuals are not available. Yet, while our TSPAN15 experiment served to demonstrate that certain TSPANs can indeed alter ADAM10 cleavage of some substrates versus others, it is unlikely that the acitretin‐stimulated, selective increase in sAPPα in the AD patients is directly related to altered TSPAN15 expression, because acitretin (increased sAPPα, no effect on sNrCAM) and TSPAN15 expression (reduced sAPPα and increased sNrCAM) had opposite effects on cleavage of APP and NrCAM. However, it is well possible that acitretin mediates its relatively specific effect through other members of the TSPAN C8 family, which comprises besides TSPAN15 also TSPANs 5, 10, 14, 17, and 33. Among them, especially TSPAN5 and TSPAN14 are highly expressed in particular cell types within the central nervous system (Matthews *et al*, 2017) and may therefore contribute to the differential acitretin effects on various ADAM10 substrates. In fact, by increasing intracellular atRA levels, acitretin can have an impact on the expression of many genes, not only ADAM10 (Lane & Bailey, 2005). However, generation of mice deficient in or transgenically expressing those TSPANs will be needed to better understand the role of the TSPANs in controlling the substrate selectivity of ADAM10. At this point, it also appears possible that acitretin mediates its effects through proteins other than TSPANs. One precedent comes from the ADAM10‐homolog ADAM17, which does not bind TSPANs, but another multi‐pass transmembrane protein, iRhom1 or iRhom2. Recent studies revealed that this binary interaction is in fact a ternary interaction with the soluble protein FRMD8/iTAP (Kunzel *et al*, 2018; Oikonomidi *et al*, 2018), which controls stability and activity of the ADAM17/iRhom complex. Thus, it appears possible that additional proteins may also affect ADAM10 binding to TSPANs and thus contribute to substrate selectivity. Such proteins may be alternative targets for acitretin.

Taken together, our current study and the previous studies demonstrate that a substrate‐selective activation of ADAM10 is in principle feasible and suggests that additional ADAM10 activators beyond acitretin may be identified in drug development for a future substrate‐selective ADAM10 activation in AD. Furthermore, our study provides a possible explanation for the lack of severe side effects upon mild ADAM10 activation.

Another major outcome of our study is the identification of sNrCAM as an excellent marker for measuring substrate‐selective ADAM10 activation. For clinical trials in AD, the activation of ADAM10 is measured through increased sAPPα levels and reduced Aβ levels. However, so far it has remained unclear whether other ADAM10 substrates would also be affected. Ideally, the effect of an ADAM10 activator would be measured on all known neuronal ADAM10 substrates, which may be difficult in a clinical routine setting. Thus, sNrCAM may serve as a proxy for other ADAM10 substrates besides APP, as its cleavage happens mechanistically differently from APP. Our study demonstrates that, identical to APP, NrCAM requires ADAM10 for its constitutive cleavage, but that an increase of substrate cleavage occurs through different mechanisms for both substrates, APP and NrCAM. Thus, sNrCAM has the potential to be a companion diagnostic for clinical trials with ADAM10 activators, as it allows distinguishing between the intended specific increase in sAPPα and a potential non‐desired increase of other ADAM10 substrate cleavage products, including NrCAM. Similar considerations hold true for the two unrelated proteases β‐ and γ‐secretase, which convert APP to the Aβ peptide. For example, γ‐secretase inhibitors, which have been discontinued for AD drug development, also block cleavage of numerous additional γ‐secretase substrates besides APP, including Notch (Golde *et al*, 2013). Thus, Notch cleavage is used to counter‐screen γ‐secretase inhibitors to identify such compounds that spare Notch cleavage but still block APP cleavage (Golde *et al*, 2013). Similarly, sNrCAM may be used to identify additional drugs besides acitretin that preferentially activate ADAM10 cleavage of APP.

Our study demonstrates that acitretin did not alter NrCAM shedding *in vitro* or *in vivo*, making it unlikely that the acitretin‐mediated increase in ADAM10 leads to mechanism‐based side effects resulting specifically from altered NrCAM function. However, our study clearly demonstrates that other ways of modulating ADAM10 levels or specifically altering ADAM10 cleavage of NrCAM have major consequences for neurite outgrowth. While ADAM10 inhibition enhanced full‐length NrCAM levels at the neuronal surface and increased neurite outgrowth in the *in vitro* assay, a knock‐down of NrCAM abolished the increased neurite outgrowth. This is consistent with previous antibody perturbation experiments which also inhibited neurite outgrowth *in vitro* and additionally disturbed axonal guidance, by interfering with the interaction between NrCAM and its respective ligands at the neuronal or glial surface (Morales *et al*, 1993; Stoeckli & Landmesser, 1995; Sakurai *et al*, 1997; Stoeckli *et al*, 1997). ADAM10 has been shown to differentially regulate neurite outgrowth and axonal guidance through the cleavage of several cell adhesion molecules other than NrCAM (e.g., L1CAM, IgLONs) in distinct anatomical locations and developmental stages (Maretzky *et al*, 2005; Saftig & Lichtenthaler, 2015; Sanz *et al*, 2017). For example, while ADAM10 inhibition decreased neurite growth in cultured DRG neurons/explants and chick retinal ganglion cells, it increased the number of neuritic processes in neuronal SH‐SY5Y cells (Paudel *et al*, 2013; Meyer *et al*, 2015; Martins *et al*, 2017; Sanz *et al*, 2017). Likewise, inhibition of L1 shedding in cerebellar microexplant cultures only affected neurite outgrowth, when the cultures were placed on L1‐coated coverslips (Maretzky *et al*, 2005). Neurite outgrowth and guidance are complexly regulated and largely rely on the temporal and spatial expression of specific receptors and ligands. Thus, it is not surprising that ADAM10 can have different effects on those processes, depending on the local expression of its respective substrates at specific time points (Kiryushko *et al*, 2004; Maness & Schachner, 2007). This study shows for the first time that ADAM10 can affect neurite outgrowth/length in cortical neurons in an NrCAM‐dependent manner. This could be of relevance in neuropsychiatric disorders, such as autism spectrum disorders (ASD) and schizophrenia, to which NrCAM is linked (Marui *et al*, 2009; Ayalew *et al*, 2012) and where neurite outgrowth and branching may be down‐regulated by NrCAM shedding in a process impairing neuronal connectivity.

An ADAM10‐dependent receptor function for full‐length NrCAM is further corroborated by the observation that mice with a conditional knock‐out of ADAM10 in neurons (ADAM10 cKO) and NrCAM‐deficient mice show opposite phenotypes with regard to dendritic spines. While ADAM10 cKO mice have a reduced number of dendritic spines (Prox *et al*, 2013), NrCAM KO mice have increased dendritic spine densities. Together with Neuropilin‐2 and Plexin A3, NrCAM is part of a Sema3F receptor complex, which is mediating a Sema3F‐induced spine retraction (Demyanenko *et al*, 2014; Mohan *et al*, 2018). Thus, increased NrCAM surface levels, as we observed upon inhibition of ADAM10, would be expected to decrease spine density, as reported for ADAM10 cKO mice. Conversely, knock‐out of NrCAM would increase spine density, as reported (Demyanenko *et al*, 2014). Taken together, these results suggest that alterations of ADAM10 activity, and therefore of NrCAM shedding and surface levels, may be a means of controlling synaptic plasticity, by strengthening the contacts of highly active synapses, while making them less susceptible to Sema3F‐induced spine retraction. Yet, given our finding that acitretin did not alter NrCAM shedding, it is likely that the physiological functions of NrCAM are not altered in clinical trials with acitretin.

Besides the function of full‐length NrCAM as a surface receptor, as discussed above, the cleavage products—either sNrCAM or the C‐terminal fragment of NrCAM or both—may also be functional, because we previously reported an olfactory axon targeting deficit in ADAM10^−/−^ mice, that was similar, if not identical to NrCAM^−/−^ mice (Heyden *et al*, 2008; Kuhn *et al*, 2016). Thus, it appears possible that both full‐length NrCAM and its cleavage product(s) may have different functions during development of the nervous system. This is reminiscent of death receptor 6, a member of the TNF receptor superfamily. Full‐length DR6 can act as a surface receptor transmitting cell death signals (Nikolaev *et al*, 2009; Mi *et al*, 2011; Strilic *et al*, 2016; Fujikura *et al*, 2017; Gamage *et al*, 2017), while the ADAM10‐cleaved DR6 ectodomain can act as a cytokine suppressing proliferation of Schwann cells and delaying myelination in the peripheral nervous system (Colombo *et al*, 2018).

In summary, our study identifies a novel function for ADAM10‐dependent processing of NrCAM in regulating neurite outgrowth. Moreover, it establishes the soluble, ADAM10‐cleaved sNrCAM as an excellent marker for substrate‐selective ADAM10 activation *in vitro* and in patients and reveals that a substrate‐selective activation of ADAM10 is feasible in patients. The ability to distinguish between the potentially detrimental activation of ADAM10/NrCAM processing and the protective ADAM10/APP processing will provide new opportunities for safe drug development in AD targeting ADAM10.

## Materials and Methods

### Materials

Antibodies: ADAM10 (1:1,000), ADAM17 (Schlondorff *et al*, 2000) (1:1,000), β‐actin (1:1,000), calnexin (1:1,000), N‐term. NrCAM (1:1,000), monoclonal human NrCAM (1:500), C‐term. NrCAM (1:1000), APP 22C11 (1:1,000), sAPPα 5G11 (1:10) and 14D6 (1:10) (Colombo *et al*, 2013), VSV‐G (Santa Cruz) (1:1,000), GluR6/7 (1:1000), hSA (1:1,000), FLAG (1:1,000) (Sigma), and HRP‐coupled anti‐mouse and anti‐rabbit secondary antibodies (DAKO) (1:10,000). Drugs and reagents: GI254023X (Sigma, 5 μM), acitretin (4 μM), NMDA (50 μM), D‐APV (Abcam, 100 μM), dec‐CMK (Bachem, 50 μM), PMA (1 μM) recombinant trypsin (Promega), recombinant LysC (Promega), iodoacetamide (Sigma), and dithiothreitol (Biozol).

### Antibodies

**Table:** 

Antibodies	Source; reference
Anti‐NrCAM (N‐terminal)	Abcam (ab24344); (Lustig *et al*, 2001)
Anti‐NrCAM (C‐terminal)	Cell Signaling (55284)
Anti‐human NrCAM (N‐terminal)	R&D (MAB20341); (Wildsmith *et al*, 2014)
Anti‐APP: 22C11 (N‐terminal)	Millipore (MAB384); (Kuhn *et al*, 2010)
Anti‐sAPPα: 5G11 and 14D6	(Colombo *et al*, 2013)
Anti‐ADAM10 (C‐terminal)	Abcam (ab124695); (Kuhn *et al*, 2016)
Anti‐ADAM17 (C‐terminal)	(Schlondorff *et al*, 2000)
Anti‐β‐actin	Sigma‐Aldrich (A5316); (Pigoni *et al*, 2016)
Anti‐calnexin	(Enzo, Stressgen, ADI‐SPA‐860); (Pigoni *et al*, 2016)
Anti‐flag M2	Sigma‐Aldrich (F1804); (Pigoni *et al*, 2016)
Anti‐hSA	Sigma‐Aldrich (A6684); (Carter‐Dawson *et al*, 2010)
Anti‐GluR 6/7 (GriK 2/3)	Abcam (ab124702); (Kung *et al*, 2013)
Anti‐VSV‐G	Santa Cruz (sc‐365019)

### Plasmids

pcINeo‐NrCAM‐VSV was kindly provided by Dr. Ben Ze've (Conacci‐Sorrell *et al*, 2005). Generation of pEF6A FLAG human TSPAN15 was described previously (Haining *et al*, 2012).

### Mouse strains and cell lines

The following mouse strains were used in this study: wild‐type (WT) C57BL/6NCrl (Charles River), ADAM10fl/fl (Prox *et al*, 2013; Kuhn *et al*, 2016), ADAM17fl/fl (Horiuchi *et al*, 2007). All mice were on a C57BL/6 background and were maintained on a 12/12‐h light–dark cycle with food and water *ad libitum*. All experimental procedures on animals were performed in accordance with the European Communities Council Directive (2010/63/EU) and in compliance with the German animal welfare law. Embryos were extracted from the pregnant mothers at E15/16. HEK293 cells were purchased from ATCC. HEK293 cells were kept in DMEM supplemented with 10% FBS and 1% penicillin/streptomycin.

### Patients

Patient samples from the prospective, randomized, placebo‐controlled phase II trial on acitretin in AD patients were analyzed in this study. The study was initiated after approval by the ethic committees, and executed in accordance with the Good Clinical Practice guidelines (Declaration of Helsinki and International Conference on Harmonization), as described previously (Endres *et al*, 2014). The trial was monitored by the IZKS (University Medical Centre, Mainz) and registered with ClinicalTrials.gov (NCT01078168). Patients provided written informed consent before enrollment.

### Isolation and treatment of primary neurons

Neurons from WT mice were prepared at E15/E16 embryos and cultured as described previously (Mitterreiter *et al*, 2010). The gender of the embryos cannot be determined at this stage. In brief, neurons from wt and ADAM10/ADAM17^Fl/Fl^ mice were prepared at E15/E16 and kept in Neurobasal^®^ medium, supplemented with l‐glutamine (0.5 mM), 1% penicillin/streptomycin, and B27. At 5 days *in vitro* (DIV), the cells were washed with PBS and the medium was replaced with fresh Neurobasal medium supplemented with l‐glutamine (0. 5 mM), 1% penicillin/streptomycin, B27, and the respective drugs. After 48 h of incubation, supernatants were collected and the cells were lysed in STET lysis buffer (50 mM Tris, pH 7.5, 150 mM NaCl, 2 mM EDTA, 1% Triton) that contained GI254023x (5 μM), to prevent an autocatalytic degradation of mADAM10 (Brummer *et al*, 2018). For NMDA treatments, cells were cultured as described earlier (Wan *et al*, 2012). For the treatment with acitretin, the neurons were treated like previously described (Tippmann *et al*, 2009). ADAM10fl/fl and ADAM17fl/fl neurons were infected with iCre, or the control lentivirus at DIV3. For NMDA treatments, cells were cultured as described earlier (Wan *et al*, 2012). For the treatment with acitretin, the neurons were treated like previously described (Tippmann *et al*, 2009). ADAM10fl/fl and ADAM17fl/fl neurons were infected with iCre, or the control lentivirus at DIV3.

### Isolation and treatment of primary rat neurons

All experimental procedures were performed in accordance with the Guidelines for Animal Experiments of the University of Tokyo. Cortical primary neurons from rat were prepared according to a previously described method. In brief, cerebral cortices were dissected from Wistar rat (embryonic day E18), dissociated in Hanks’ balanced salt solution (HBSS, Life Technologies, Darmstadt, Germany) including 0.125% trypsin and 5 U/ml DNAse I (both Life Technologies, Darmstadt, Germany) and incubated at 37°C for 15 min. After the addition of DMEM medium containing 10% FBS, 2 mM l‐glutamine, and 50 U/ml penicillin/50 μg/ml streptomycin (all Life Technologies, Darmstadt, Germany), cells were pipetted through a cell strainer (100 μm, BD Falcon, Heidelberg, Germany) and subsequently centrifuged at 4°C, 200 *g* for 5 min. Cells were suspended in fresh DMEM culture medium and seeded in a concentration of 1 × 10^6^ cells/ml on poly‐L‐ornithine‐coated plates (PLO: Sigma, St. Louis, MO, USA). After day 1 *in vitro* (DIV1), medium was exchanged to Neurobasal medium including B27 supplement mix (both Life Technologies, Darmstadt, Germany), 1% glutamine, and 50 U/ml penicillin/50 μg/ml streptomycin. Neurons were cultured for 7 days at 37°C, 5% CO_2_, and 95% humidity. Cells were treated with acitretin (2 μM) at DIV19, medium was and fresh substances were added every day as described previously (Reinhardt *et al*, 2016).

### Virus production

iCre recombinase lentiviruses were prepared as previously described (Kuhn *et al*, 2016). iCre recombinase lentiviruses were prepared as previously described (Kuhn *et al*, 2016). The following shRNA sequences were used: shRNA1: 5′CGCGTCCGGGGACACCCGTGAGGACTATATCTCGAGATATAGTCCTCACGGGTGTCCTTTTTGGAAA′3; shRNA2: 5′CGCGTCCGGATAGATGGCGATACCATTATACTCGAGTATAATGGTATCGCCATCTATTTTTTGGAAA′3. Targeting sequences, as well as scrambled control sequence, were cloned into plKO2mod‐EGFP‐WPRE, as previously described (Kuhn *et al*, 2010).

### Transfection

10^6^ HEK293 cells per well were seeded in a 6‐well format and transfected in solution in OptiMEM (Gibco) with the respective vectors (600 ng), using Lipofectamine 2000 (Thermo Fisher)—as to the companies instructions—and incubated for 48 h. Then, the cells were either treated with the respective substances or lysed directly.

### Cell toxicity assay

Wt neurons were placed in a 96‐well format (20,000 cells per well); 4 h after plating were either infected with a lentivirus (scr. shRNA‐EGFP control, or NrCAM shRNA‐EGFP (shRNA1 and shRNA2), 1:1,000) or not. At DIV3, NaOH was added to the cells serving as negative control for cell viability. At DIV4, cell viability was analyzed using the Cell Counting Kit 8 (96992, Sigma‐Aldrich) according to the provided instructions. The absorbance was measured with a microplate reader at 450 nm. The individual measurements were performed in 1‐h steps, up to 3 h. The values from the initial measurement served as baseline values, and the latter values were normalized on the baseline.

### Cell surface biotinylation

Surface biotinylation was done as described previously (Pigoni *et al*, 2016).

### Cell lysate/supernatant preparation

First, supernatants from HEK293 and neurons were collected. For NMDA (30‐min treatment)‐, PMA (2‐h treatment)‐, and acitretin (5 h of conditioned media collection)‐treated cells, the supernatants were subjected to TCA precipitation (50 μl TCA/1 ml supernatant) and incubated over‐night at 4°C. Then, the samples were centrifuged at 16,000 *g* at 4°C for 5 min, and the pellets were resuspended in 1× Laemmli buffer (2% SDS; 10% glycerol; 0.00625% bromophenol blue; 2.5% β‐mercaptoethanol; 31.25 mM Tris; pH 6.8). Cells were washed twice with PBS and lysed in STET lysis buffer (50 mM Tris, pH 7.5, 150 mM NaCl, 2 mM EDTA, 1% Triton), containing protease inhibitor cocktail (1:500, Sigma, P‐8340) and GI254023X (5 μM) (Brummer *et al*, 2018). Samples were centrifuged at 16,000 *g* and 4°C for 5 min, the supernatants were transferred to fresh tubes, and the protein concentration was measured using a BCA (Uptima Interchim, UP95425).

### Quantitative real‐time PCRs

NrCAM and ADAM10 qPCR primers were purchased from Bio‐Rad (PrimePCR SYBR Green Assay: Adam10, Mouse; PrimePCR SYBR Green Assay: Nrcam, Mouse). Total RNA was extracted using RNeasy Mini Kit (Qiagen) from primary neurons following the manufacturer's instructions. Concentrations and purities of total RNA were spectrophotometrically measured at 260 and 280 nm. Total RNA was reverse transcribed into cDNA, using high‐capacity cDNA reverse transcription kit (Applied Biosystems/ABI). Real‐time PCR was carried out on a 7500 Fast Real‐Time PCR machine (ABI) with the POWER SYBR Green PCR Master Mix (ABI). Reactions were performed in duplicate in 96‐well plates. mRNA levels of ADAM10 and NrCAM were normalized on levels of a housekeeping gene (β‐actin).

### Co‐immunoprecipitations

NrCAM‐VSV‐transfected HEK293 cells were lysed in CoIP buffer (20 mM HEPES, 150 mM NaCl, 0.5% NP‐40, 2 mM EDTA, 10% glycerol) containing protease inhibitor cocktail. Samples were precleared by rotating them with protein G beads (50 μl per 500 μl lysate) at 4°C for 1 h. Then, the samples were pulled down over‐night at 4°C with a VSV‐G (1:100) or NrCAM antibody (1:100). Samples were centrifuged at 1,500 *g* at 4°C, the supernatant was removed, and the beads were washed twice with CoIP buffer and once with 1× PBS. The samples were then resuspended in 1× Laemmli buffer. The detection was done with the respective opposite antibody.

### Western blotting

The protein concentration was measured using a BCA, and equal protein concentrations for every sample were then subjected to SDS–PAGE separation. Hence, the amount of sample per lane was normalized to the total protein concentration of each sample. 15–20 μg of total protein was used for SDS–PAGE separation. In order to have a second line of control, we also blotted for a housekeeping gene (actin or calnexin), to assure the initial normalization was done properly. Samples were boiled at 95°C for 5 min in reducing (8% SDS; 40% glycerol; 0.025% bromophenol blue; 10% β‐mercaptoethanol; 125 mM Tris; pH 6.8) or non‐reducing (8% SDS; 40% glycerol; 0.025% bromophenol blue; 125 mM Tris; pH 6.8) Laemmli buffer and then separated on 8% SDS–PAGE gels. The proteins were transferred to PVDF membranes (Millipore) and blocked with 5% milk for 1 h at room temperature. Membranes were incubated for 1 h, or over‐night at 4°C with the primary antibody solutions. Then, the membranes were incubated with the secondary antibody for 45 min at room temperature. Membranes were developed with ECL prime (GE Healthcare, RPN2232V1). Western blots were quantified by Multi Gauge software (version 3.0). The values of the band intensity were normalized to the respective control values for each experiment.

### Neurite outgrowth assay

1 × 10^5^ neurons were plated into XONA‐microfluidic chambers (standard neuron device, SND450) that had been placed on PDL‐coated coverslips, according to the manufacturer's instructions. After 4 h, the plated cells were infected with the respective viruses (scr. shRNA‐EGFP control, or NrCAM shRNA‐EGFP, 1:1000). Cells were kept until DIV3 at 37°C and 5% CO_2_, and then, the first photomicrographs of the fluorescent neurons were taken with a Leica DM6000 inverted microscope. The images covered the whole channel area in the middle of the respective chambers. Afterward, the neurons were treated with GI254023x (5 μM), or vehicle and kept at 37°C and 5% CO_2_ for 24 h. At DIV4, a second set of photomicrographs of the same areas were taken and analyzed for length differences of single neurites (length in mm at 24–0 h) with Leica LASX software. Only neurites that had already entered and not yet left the channels on the other side at the timepoint 0 h were used for the calculation. When neurites were separating after leaving the main channel, the longest process was quantified.

### Mass spectrometry

CSF from nine patients per group treated with either acitretin or vehicle control was collected before (baseline value) and after the treatment (Endres *et al*, 2014). The CSF analysis was blinded. A volume of 5 μl per sample was subjected to tryptic digestion followed by liquid chromatography–tandem mass spectrometry (LC‐MS/MS) for protein label‐free quantification (LFQ) as previously described (Pigoni *et al*, 2016). Nine patients per group were treated with either acitretin or vehicle control. Cerebrospinal fluid was collected before (baseline value) and after treatment. A volume of 5 μl of CSF per sample was subjected to proteolytic digestion in 50 mM ammonium bicarbonate with 0.1% sodium deoxycholate (Sigma‐Aldrich, Germany) as previously described (Pigoni *et al*, 2016). Briefly, protein disulfide bonds were reduced with dithiothreitol and sulfhydryl residues were alkylated using iodoacetamide. Proteins were digested using 0.1 μg LysC (Promega) and 0.1 μg trypsin (Promega). Deoxycholate was precipitated by acidification and removed by centrifugation at 16,000 *g* and 4°C for 10 min. Proteolytic peptides were desalted by stop and go extraction (STAGE) with C18 tips (Rappsilber *et al*, 2003). The purified peptides were dried by vacuum centrifugation. Samples were dissolved in 20 μl 0.1% formic acid.

Samples were separated on a nanoLC system (EASY‐nLC 1000, Proxeon—part of Thermo Scientific, USA; PRSO‐V1 column oven: Sonation, Germany) using an in‐house packed C18 column (30 cm × 75 μm ID, ReproSil‐Pur 120 C18‐AQ, 1.9 μm, Dr. Maisch GmbH, Germany) with a binary gradient of water (A) and acetonitrile (B) containing 0.1% formic acid at 50°C column temperature and a flow of 250 nl/min (0 min, 2% B; 3:30 min, 5% B; 137:30 min, 25% B; 168:30 min, 35% B; 182:30 min, 60% B). The nanoLC was coupled online via a nanospray flex ion source (Proxeon—part of Thermo Scientific, USA) to a Q‐Exactive mass spectrometer (Thermo Scientific, USA). Full MS spectra were acquired at a resolution of 70,000. The ten most intense ions exceeding an intensity of 1.5 × 10^4^ were chosen for collision induced dissociation, and spectra were acquired at a resolution of 17,500. The dynamic exclusion for peptide fragmentation was set to 120 s. The data were analyzed with the software Maxquant (maxquant.org, Max‐Planck Institute Munich) version 1.5.3.12. The MS data were searched against a reviewed fasta database of *Homo sapiens* from UniProt including isoforms (download: January 09, 2017, 42120 entries). Trypsin was defined as protease. Two missed cleavages were allowed for the database search. Carbamidomethylation of cysteine was defined as static modification. Acetylation of the proteins N‐terminus and oxidation of methionine were set as variable modifications. The false discovery rate for both peptides and proteins was adjusted to < 1% using a target and decoy approach (concatenated forward/reverse database). Razor and unique peptides were used for quantification. Label‐free quantification (LFQ) of proteins required at least two ratio counts of razor or unique peptides.

### Statistical analysis

All tests were done on an exploratory 2‐sided 5% significance level. *P*‐values for Western blots were calculated with relative values using two‐sided Student's *t*‐test with a Welch's correction or one‐way ANOVA with Dunnett's multiple comparison test for multiple hypothesis testing (software GraphPad Prism 7). Normal distribution was assumed.

For mass spectrometry, protein LFQ intensities after the treatment were divided by the related baseline values of the individual patient. The LFQ ratios (treatment/baseline) were log2‐transformed, and a two‐sided Student's *t*‐test was applied to calculate significance levels for each protein between the acitretin‐treated and the vehicle control groups. The sample size was chosen by the expected effect size of the respective experiments. No samples were excluded. The sample size of the CSF samples from human patients was chosen according to the availability of the material.

The CSF samples from patients treated with acitretin were analyzed blinded.

## Author contributions

TB, FP‐M, and SFL designed the research; TB, FP‐M, and SAM analyzed the data; TB, SAM, and FY performed the research; TB and SFL wrote the paper; and FP‐M, AF, TT, and KE contributed new important reagents or analytic tools.

## Conflict of interest

The authors declare that they have no conflict of interest.

## For more information

- https://www.dzne.de/en/research/research-areas/fundamental-research/research groups/lichtenthaler
- Acitretin in AD: https://www.alzforum.org/therapeutics/acitretin
- ADAM10 in AD: https://www.alzforum.org/alzpedia/adam10
- ADAM10 gene: https://www.ncbi.nlm.nih.gov/gene/102
- ADAM10 protein: https://www.uniprot.org/uniprot/O14672
- NRCAM gene: https://www.ncbi.nlm.nih.gov/gene/4897
- NrCAM protein: https://www.uniprot.org/uniprot/Q92823

## Supporting information



Expanded View Figures PDF

Dataset EV1

Source Data for Expanded View

Review Process File

Source Data for Figure 1

Source Data for Figure 2

Source Data for Figure 3

Source Data for Figure 4

Source Data for Figure 5

Source Data for Figure 7

## Data Availability

Proteomics: The mass spectrometry proteomics data have been deposited to the ProteomeXchange Consortium via the PRIDE partner repository with the dataset identifier PXD010756. Imaging: The raw images of the neurite outgrowth experiment have been deposited to the Zenodo platform with https://doi.org/10.5281/zenodo.1344972.

## References

[emmm201809695-bib-0001] Anders A , Gilbert S , Garten W , Postina R , Fahrenholz F (2001) Regulation of the alpha‐secretase ADAM10 by its prodomain and proprotein convertases. FASEB J 15: 1837–1839 11481247 10.1096/fj.01-0007fje

[emmm201809695-bib-0002] Ayalew M , Le‐Niculescu H , Levey DF , Jain N , Changala B , Patel SD , Winiger E , Breier A , Shekhar A , Amdur R *et al* (2012) Convergent functional genomics of schizophrenia: from comprehensive understanding to genetic risk prediction. Mol Psychiatry 17: 887–905 22584867 10.1038/mp.2012.37 PMC3427857

[emmm201809695-bib-0003] Bozkulak EC , Weinmaster G (2009) Selective use of ADAM10 and ADAM17 in activation of Notch1 signaling. Mol Cell Biol 29: 5679–5695 19704010 10.1128/MCB.00406-09 PMC2772745

[emmm201809695-bib-0004] Brummer T , Pigoni M , Rossello A , Wang H , Noy PJ , Tomlinson MG , Blobel CP , Lichtenthaler SF (2018) The metalloprotease ADAM10 (a disintegrin and metalloprotease 10) undergoes rapid, postlysis autocatalytic degradation. FASEB J 32: 3560–3573 29430990 10.1096/fj.201700823RR PMC5998973

[emmm201809695-bib-0005] Burian M , Schittek B (2015) The secrets of dermcidin action. Int J Med Microbiol 305: 283–286 25596890 10.1016/j.ijmm.2014.12.012

[emmm201809695-bib-0006] Cai Z , Zhang A , Choksi S , Li W , Li T , Zhang XM , Liu ZG (2016) Activation of cell‐surface proteases promotes necroptosis, inflammation and cell migration. Cell Res 26: 886–900 27444869 10.1038/cr.2016.87 PMC4973336

[emmm201809695-bib-0007] Carter‐Dawson L , Zhang Y , Harwerth RS , Rojas R , Dash P , Zhao XC , WoldeMussie E , Ruiz G , Chuang A , Dubinsky WP *et al* (2010) Elevated albumin in retinas of monkeys with experimental glaucoma. Invest Ophthalmol Vis Sci 51: 952–959 19797225 10.1167/iovs.09-4331 PMC2868465

[emmm201809695-bib-0008] Colombo A , Wang H , Kuhn PH , Page R , Kremmer E , Dempsey PJ , Crawford HC , Lichtenthaler SF (2013) Constitutive alpha‐ and beta‐secretase cleavages of the amyloid precursor protein are partially coupled in neurons, but not in frequently used cell lines. Neurobiol Dis 49: 137–147 22940630 10.1016/j.nbd.2012.08.011 PMC4310234

[emmm201809695-bib-0009] Colombo A , Hsia HE , Wang M , Kuhn PH , Brill MS , Canevazzi P , Feederle R , Taveggia C , Misgeld T , Lichtenthaler SF (2018) Non‐cell‐autonomous function of DR6 in Schwann cell proliferation. EMBO J 37: e97390 29459438 10.15252/embj.201797390 PMC5881626

[emmm201809695-bib-0010] Conacci‐Sorrell M , Kaplan A , Raveh S , Gavert N , Sakurai T , Ben‐Ze'ev A (2005) The shed ectodomain of Nr‐CAM stimulates cell proliferation and motility, and confers cell transformation. Can Res 65: 11605–11612 10.1158/0008-5472.CAN-05-2647 16357171

[emmm201809695-bib-0011] Crawford HC , Dempsey PJ , Brown G , Adam L , Moss ML (2009) ADAM10 as a therapeutic target for cancer and inflammation. Curr Pharm Des 15: 2288–2299 19601831 10.2174/138161209788682442

[emmm201809695-bib-0012] Dai J , Buhusi M , Demyanenko GP , Brennaman LH , Hruska M , Dalva MB , Maness PF (2013) Neuron glia‐related cell adhesion molecule (NrCAM) promotes topographic retinocollicular mapping. PLoS ONE 8: e73000 24023801 10.1371/journal.pone.0073000 PMC3759449

[emmm201809695-bib-0013] Dash‐Wagh S , Neumann JR , Veitinger S , Grote‐Westrick C , Landgraf P , Pape HC , Kreutz MR , von Holst A , Wahle P (2011) The survival promoting peptide Y‐P30 promotes cellular migration. Mol Cell Neurosci 48: 195–204 21820515 10.1016/j.mcn.2011.07.005

[emmm201809695-bib-0014] Davis JQ , Bennett V (1994) Ankyrin binding activity shared by the neurofascin/L1/NrCAM family of nervous system cell adhesion molecules. J Biol Chem 269: 27163–27166 7961622

[emmm201809695-bib-0015] Demyanenko GP , Riday TT , Tran TS , Dalal J , Darnell EP , Brennaman LH , Sakurai T , Grumet M , Philpot BD , Maness PF (2011) NrCAM deletion causes topographic mistargeting of thalamocortical axons to the visual cortex and disrupts visual acuity. J Neurosci 31: 1545–1558 21273439 10.1523/JNEUROSCI.4467-10.2011 PMC3037548

[emmm201809695-bib-0016] Demyanenko GP , Mohan V , Zhang X , Brennaman LH , Dharbal KE , Tran TS , Manis PB , Maness PF (2014) Neural cell adhesion molecule NrCAM regulates Semaphorin 3F‐induced dendritic spine remodeling. J Neurosci 34: 11274–11287 25143608 10.1523/JNEUROSCI.1774-14.2014 PMC4138338

[emmm201809695-bib-0017] Dovey HF , John V , Anderson JP , Chen LZ , de Saint Andrieu P , Fang LY , Freedman SB , Folmer B , Goldbach E , Holsztynska EJ *et al* (2001) Functional gamma‐secretase inhibitors reduce beta‐amyloid peptide levels in brain. J Neurochem 76: 173–181 11145990 10.1046/j.1471-4159.2001.00012.x

[emmm201809695-bib-0018] Endres K , Postina R , Schroeder A , Mueller U , Fahrenholz F (2005) Shedding of the amyloid precursor protein‐like protein APLP2 by disintegrin‐metalloproteinases. FEBS J 272: 5808–5820 16279945 10.1111/j.1742-4658.2005.04976.x

[emmm201809695-bib-0019] Endres K , Fahrenholz F , Lotz J , Hiemke C , Teipel S , Lieb K , Tuscher O , Fellgiebel A (2014) Increased CSF APPs‐alpha levels in patients with Alzheimer disease treated with acitretin. Neurology 83: 1930–1935 25344383 10.1212/WNL.0000000000001017

[emmm201809695-bib-0020] Epis R , Marcello E , Gardoni F , Vastagh C , Malinverno M , Balducci C , Colombo A , Borroni B , Vara H , Dell'Agli M *et al* (2010) Blocking ADAM10 synaptic trafficking generates a model of sporadic Alzheimer's disease. Brain 133: 3323–3335 20805102 10.1093/brain/awq217

[emmm201809695-bib-0021] Falk J , Thoumine O , Dequidt C , Choquet D , Faivre‐Sarrailh C (2004) NrCAM coupling to the cytoskeleton depends on multiple protein domains and partitioning into lipid rafts. Mol Biol Cell 15: 4695–4709 15254265 10.1091/mbc.E04-03-0171 PMC519160

[emmm201809695-bib-0022] Freese C , Garratt AN , Fahrenholz F , Endres K (2009) The effects of alpha‐secretase ADAM10 on the proteolysis of neuregulin‐1. FEBS J 276: 1568–1580 19220854 10.1111/j.1742-4658.2009.06889.x

[emmm201809695-bib-0023] Fujikura D , Ikesue M , Endo T , Chiba S , Higashi H , Uede T (2017) Death receptor 6 contributes to autoimmunity in lupus‐prone mice. Nat Commun 8: 13957 28045014 10.1038/ncomms13957 PMC5216082

[emmm201809695-bib-0024] Gamage KK , Cheng I , Park RE , Karim MS , Edamura K , Hughes C , Spano AJ , Erisir A , Deppmann CD (2017) Death receptor 6 promotes wallerian degeneration in peripheral axons. Curr Biol 27: 890–896 28285993 10.1016/j.cub.2017.01.062 PMC5360522

[emmm201809695-bib-0025] Golde TE , Koo EH , Felsenstein KM , Osborne BA , Miele L (2013) gamma‐Secretase inhibitors and modulators. Biochem Biophys Acta 1828: 2898–2907 23791707 10.1016/j.bbamem.2013.06.005 PMC3857966

[emmm201809695-bib-0026] Grumet M , Mauro V , Burgoon MP , Edelman GM , Cunningham BA (1991) Structure of a new nervous system glycoprotein, Nr‐CAM, and its relationship to subgroups of neural cell adhesion molecules. J Cell Biol 113: 1399–1412 2045418 10.1083/jcb.113.6.1399 PMC2289038

[emmm201809695-bib-0027] Haining EJ , Yang J , Bailey RL , Khan K , Collier R , Tsai S , Watson SP , Frampton J , Garcia P , Tomlinson MG (2012) The TspanC8 subgroup of tetraspanins interacts with A disintegrin and metalloprotease 10 (ADAM10) and regulates its maturation and cell surface expression. J Biol Chem 287: 39753–39765 23035126 10.1074/jbc.M112.416503 PMC3501075

[emmm201809695-bib-0028] Heyden A , Angenstein F , Sallaz M , Seidenbecher C , Montag D (2008) Abnormal axonal guidance and brain anatomy in mouse mutants for the cell recognition molecules close homolog of L1 and NgCAM‐related cell adhesion molecule. Neuroscience 155: 221–233 18588951 10.1016/j.neuroscience.2008.04.080

[emmm201809695-bib-0029] Hinkle CL , Diestel S , Lieberman J , Maness PF (2006) Metalloprotease‐induced ectodomain shedding of neural cell adhesion molecule (NCAM). J Neurobiol 66: 1378–1395 16967505 10.1002/neu.20257

[emmm201809695-bib-0030] Hogl S , Kuhn PH , Colombo A , Lichtenthaler SF (2011) Determination of the proteolytic cleavage sites of the amyloid precursor‐like protein 2 by the proteases ADAM10, BACE1 and gamma‐secretase. PLoS ONE 6: e21337 21695060 10.1371/journal.pone.0021337 PMC3117885

[emmm201809695-bib-0031] Hohoff C , Borchers T , Rustow B , Spener F , van Tilbeurgh H (1999) Expression, purification, and crystal structure determination of recombinant human epidermal‐type fatty acid binding protein. Biochemistry 38: 12229–12239 10493790 10.1021/bi990305u

[emmm201809695-bib-0032] Horiuchi K , Kimura T , Miyamoto T , Takaishi H , Okada Y , Toyama Y , Blobel CP (2007) Cutting edge: TNF‐alpha‐converting enzyme (TACE/ADAM17) inactivation in mouse myeloid cells prevents lethality from endotoxin shock. J Immunol 179: 2686–2689 17709479 10.4049/jimmunol.179.5.2686

[emmm201809695-bib-0033] Hu WT , Chen‐Plotkin A , Arnold SE , Grossman M , Clark CM , Shaw LM , Pickering E , Kuhn M , Chen Y , McCluskey L *et al* (2010) Novel CSF biomarkers for Alzheimer's disease and mild cognitive impairment. Acta Neuropathol 119: 669–678 20232070 10.1007/s00401-010-0667-0 PMC2880811

[emmm201809695-bib-0034] Hundhausen C , Misztela D , Berkhout TA , Broadway N , Saftig P , Reiss K , Hartmann D , Fahrenholz F , Postina R , Matthews V *et al* (2003) The disintegrin‐like metalloproteinase ADAM10 is involved in constitutive cleavage of CX3CL1 (fractalkine) and regulates CX3CL1‐mediated cell‐cell adhesion. Blood 102: 1186–1195 12714508 10.1182/blood-2002-12-3775

[emmm201809695-bib-0035] Jorissen E , Prox J , Bernreuther C , Weber S , Schwanbeck R , Serneels L , Snellinx A , Craessaerts K , Thathiah A , Tesseur I *et al* (2010) The disintegrin/metalloproteinase ADAM10 is essential for the establishment of the brain cortex. J Neurosci 30: 4833–4844 20371803 10.1523/JNEUROSCI.5221-09.2010 PMC2921981

[emmm201809695-bib-0036] Jouannet S , Saint‐Pol J , Fernandez L , Nguyen V , Charrin S , Boucheix C , Brou C , Milhiet PE , Rubinstein E (2016) TspanC8 tetraspanins differentially regulate the cleavage of ADAM10 substrates, Notch activation and ADAM10 membrane compartmentalization. Cell Mol Life Sci 73: 1895–1915 26686862 10.1007/s00018-015-2111-z PMC4819958

[emmm201809695-bib-0037] Jung YS , Kim KS , Kim KD , Lim JS , Kim JW , Kim E (2001) Apoptosis‐linked gene 2 binds to the death domain of Fas and dissociates from Fas during Fas‐mediated apoptosis in Jurkat cells. Biochem Biophys Res Comm 288: 420–426 11606059 10.1006/bbrc.2001.5769

[emmm201809695-bib-0038] Kayyem JF , Roman JM , de la Rosa EJ , Schwarz U , Dreyer WJ (1992) Bravo/Nr‐CAM is closely related to the cell adhesion molecules L1 and Ng‐CAM and has a similar heterodimer structure. J Cell Biol 118: 1259–1270 1512296 10.1083/jcb.118.5.1259 PMC2289593

[emmm201809695-bib-0039] Kim M , Suh J , Romano D , Truong MH , Mullin K , Hooli B , Norton D , Tesco G , Elliott K , Wagner SL *et al* (2009) Potential late‐onset Alzheimer's disease‐associated mutations in the ADAM10 gene attenuate {alpha}‐secretase activity. Hum Mol Genet 18: 3987–3996 19608551 10.1093/hmg/ddp323 PMC2748890

[emmm201809695-bib-0040] Kim J , Lilliehook C , Dudak A , Prox J , Saftig P , Federoff HJ , Lim ST (2010) Activity‐dependent alpha‐cleavage of nectin‐1 is mediated by a disintegrin and metalloprotease 10 (ADAM10). J Biol Chem 285: 22919–22926 20501653 10.1074/jbc.M110.126649 PMC2906284

[emmm201809695-bib-0041] Kiryushko D , Berezin V , Bock E (2004) Regulators of neurite outgrowth: role of cell adhesion molecules. Ann N Y Acad Sci 1014: 140–154 15153429 10.1196/annals.1294.015

[emmm201809695-bib-0042] Kuhn PH , Wang H , Dislich B , Colombo A , Zeitschel U , Ellwart JW , Kremmer E , Rossner S , Lichtenthaler SF (2010) ADAM10 is the physiologically relevant, constitutive alpha‐secretase of the amyloid precursor protein in primary neurons. EMBO J 29: 3020–3032 20676056 10.1038/emboj.2010.167 PMC2944055

[emmm201809695-bib-0043] Kuhn PH , Voss M , Haug‐Kroper M , Schroder B , Schepers U , Brase S , Haass C , Lichtenthaler SF , Fluhrer R (2015) Secretome analysis identifies novel signal peptide peptidase‐like 3 (Sppl3) substrates and reveals a role of Sppl3 in multiple Golgi glycosylation pathways. Mol Cell Proteomics 14: 1584–1598 25827571 10.1074/mcp.M115.048298 PMC4458722

[emmm201809695-bib-0044] Kuhn PH , Colombo AV , Schusser B , Dreymueller D , Wetzel S , Schepers U , Herber J , Ludwig A , Kremmer E , Montag D *et al* (2016) Systematic substrate identification indicates a central role for the metalloprotease ADAM10 in axon targeting and synapse function. Elife 5: e12748 26802628 10.7554/eLife.12748 PMC4786429

[emmm201809695-bib-0045] Kung LH , Gong K , Adedoyin M , Ng J , Bhargava A , Ohara PT , Jasmin L (2013) Evidence for glutamate as a neuroglial transmitter within sensory ganglia. PLoS ONE 8: e68312 23844184 10.1371/journal.pone.0068312 PMC3699553

[emmm201809695-bib-0046] Kunzel U , Grieve AG , Meng Y , Sieber B , Cowley SA , Freeman M (2018) FRMD8 promotes inflammatory and growth factor signalling by stabilising the iRhom/ADAM17 sheddase complex. Elife 7: e35012 29897336 10.7554/eLife.35012 PMC6042961

[emmm201809695-bib-0047] Kuwajima T , Yoshida Y , Takegahara N , Petros TJ , Kumanogoh A , Jessell TM , Sakurai T , Mason C (2012) Optic chiasm presentation of Semaphorin6D in the context of Plexin‐A1 and Nr‐CAM promotes retinal axon midline crossing. Neuron 74: 676–690 22632726 10.1016/j.neuron.2012.03.025 PMC3361695

[emmm201809695-bib-0048] Lammich S , Kojro E , Postina R , Gilbert S , Pfeiffer R , Jasionowski M , Haass C , Fahrenholz F (1999) Constitutive and regulated alpha‐secretase cleavage of Alzheimer's amyloid precursor protein by a disintegrin metalloprotease. Proc Natl Acad Sci USA 96: 3922–3927 10097139 10.1073/pnas.96.7.3922 PMC22396

[emmm201809695-bib-0049] Lane MA , Bailey SJ (2005) Role of retinoid signalling in the adult brain. Prog Neurobiol 75: 275–293 15882777 10.1016/j.pneurobio.2005.03.002

[emmm201809695-bib-0050] Lichtenthaler SF , Haass C , Steiner H (2011) Regulated intramembrane proteolysis–lessons from amyloid precursor protein processing. J Neurochem 117: 779–796 21413990 10.1111/j.1471-4159.2011.07248.x

[emmm201809695-bib-0051] Lichtenthaler SF , Lemberg MK , Fluhrer R (2018) Proteolytic ectodomain shedding of membrane proteins in mammals‐hardware, concepts, and recent developments. EMBO J 37: e99456 29976761 10.15252/embj.201899456 PMC6068445

[emmm201809695-bib-0052] Lopez‐Perez E , Zhang Y , Frank SJ , Creemers J , Seidah N , Checler F (2001) Constitutive alpha‐secretase cleavage of the beta‐amyloid precursor protein in the furin‐deficient LoVo cell line: involvement of the pro‐hormone convertase 7 and the disintegrin metalloprotease ADAM10. J Neurochem 76: 1532–1539 11238737 10.1046/j.1471-4159.2001.00180.x

[emmm201809695-bib-0053] Ludwig A , Hundhausen C , Lambert MH , Broadway N , Andrews RC , Bickett DM , Leesnitzer MA , Becherer JD (2005) Metalloproteinase inhibitors for the disintegrin‐like metalloproteinases ADAM10 and ADAM17 that differentially block constitutive and phorbol ester‐inducible shedding of cell surface molecules. Comb Chem High Throughput Screen 8: 161–171 15777180 10.2174/1386207053258488

[emmm201809695-bib-0054] Lustig M , Erskine L , Mason CA , Grumet M , Sakurai T (2001) Nr‐CAM expression in the developing mouse nervous system: ventral midline structures, specific fiber tracts, and neuropilar regions. J Comp Neurol 434: 13–28 11329126 10.1002/cne.1161

[emmm201809695-bib-0055] Maness PF , Schachner M (2007) Neural recognition molecules of the immunoglobulin superfamily: signaling transducers of axon guidance and neuronal migration. Nat Neurosci 10: 19–26 17189949 10.1038/nn1827

[emmm201809695-bib-0056] Marcello E , Gardoni F , Mauceri D , Romorini S , Jeromin A , Epis R , Borroni B , Cattabeni F , Sala C , Padovani A *et al* (2007) Synapse‐associated protein‐97 mediates alpha‐secretase ADAM10 trafficking and promotes its activity. J Neurosci 27: 1682–1691 17301176 10.1523/JNEUROSCI.3439-06.2007 PMC6673742

[emmm201809695-bib-0057] Marcello E , Borroni B , Pelucchi S , Gardoni F , Di Luca M (2017) ADAM10 as a therapeutic target for brain diseases: from developmental disorders to Alzheimer's disease. Expert Opin Ther Targets 21: 1017–1026 28960088 10.1080/14728222.2017.1386176

[emmm201809695-bib-0058] Maretzky T , Schulte M , Ludwig A , Rose‐John S , Blobel C , Hartmann D , Altevogt P , Saftig P , Reiss K (2005) L1 is sequentially processed by two differently activated metalloproteases and presenilin/gamma‐secretase and regulates neural cell adhesion, cell migration, and neurite outgrowth. Mol Cell Biol 25: 9040–9053 16199880 10.1128/MCB.25.20.9040-9053.2005 PMC1265787

[emmm201809695-bib-0059] Martins F , Serrano JB , Muller T , da Cruz ESOAB , Rebelo S (2017) BRI2 processing and its neuritogenic role are modulated by protein phosphatase 1 complexing. J Cell Biochem 118: 2752–2763 28176357 10.1002/jcb.25925

[emmm201809695-bib-0060] Marui T , Funatogawa I , Koishi S , Yamamoto K , Matsumoto H , Hashimoto O , Nanba E , Nishida H , Sugiyama T , Kasai K *et al* (2009) Association of the neuronal cell adhesion molecule (NRCAM) gene variants with autism. Int J Neuropsychopharmacol 12: 1–10 18664314 10.1017/S1461145708009127

[emmm201809695-bib-0061] Matthews AL , Szyroka J , Collier R , Noy PJ , Tomlinson MG (2017) Scissor sisters: regulation of ADAM10 by the TspanC8 tetraspanins. Biochem Soc Trans 45: 719–730 28620033 10.1042/BST20160290 PMC5473022

[emmm201809695-bib-0062] Meyer zu Horste G , Derksen A , Stassart R , Szepanowski F , Thanos M , Stettner M , Boettcher C , Lehmann HC , Hartung HP , Kieseier BC (2015) Neuronal ADAM10 promotes outgrowth of small‐caliber myelinated axons in the peripheral nervous system. J Neuropathol Exp Neurol 74: 1077–1085 26426268 10.1097/NEN.0000000000000253

[emmm201809695-bib-0063] Mi S , Lee X , Hu Y , Ji B , Shao Z , Yang W , Huang G , Walus L , Rhodes K , Gong BJ *et al* (2011) Death receptor 6 negatively regulates oligodendrocyte survival, maturation and myelination. Nat Med 17: 816–821 21725297 10.1038/nm.2373

[emmm201809695-bib-0064] Mitterreiter S , Page RM , Kamp F , Hopson J , Winkler E , Ha HR , Hamid R , Herms J , Mayer TU , Nelson DJ *et al* (2010) Bepridil and amiodarone simultaneously target the Alzheimer's disease beta‐ and gamma‐secretase via distinct mechanisms. J Neurosci 30: 8974–8983 20592218 10.1523/JNEUROSCI.1199-10.2010 PMC6632893

[emmm201809695-bib-0065] Mohan V , Sullivan CS , Guo J , Wade SD , Majumder S , Agarwal A , Anton ES , Temple BS , Maness PF (2018) Temporal regulation of dendritic spines through NrCAM‐Semaphorin3F receptor signaling in developing cortical pyramidal neurons. Cereb Cortex 29: 963–977 10.1093/cercor/bhy004 PMC6499012 29415226

[emmm201809695-bib-0066] Morales G , Hubert M , Brummendorf T , Treubert U , Tarnok A , Schwarz U , Rathjen FG (1993) Induction of axonal growth by heterophilic interactions between the cell surface recognition proteins F11 and Nr‐CAM/Bravo. Neuron 11: 1113–1122 8274278 10.1016/0896-6273(93)90224-f

[emmm201809695-bib-0067] Nawabi H , Briancon‐Marjollet A , Clark C , Sanyas I , Takamatsu H , Okuno T , Kumanogoh A , Bozon M , Takeshima K , Yoshida Y *et al* (2010) A midline switch of receptor processing regulates commissural axon guidance in vertebrates. Genes Dev 24: 396–410 20159958 10.1101/gad.542510 PMC2816738

[emmm201809695-bib-0068] Nikolaev A , McLaughlin T , O'Leary DD , Tessier‐Lavigne M (2009) APP binds DR6 to trigger axon pruning and neuron death via distinct caspases. Nature 457: 981–989 19225519 10.1038/nature07767 PMC2677572

[emmm201809695-bib-0069] Noy PJ , Yang J , Reyat JS , Matthews AL , Charlton AE , Furmston J , Rogers DA , Rainger GE , Tomlinson MG (2016) TspanC8 tetraspanins and A disintegrin and metalloprotease 10 (ADAM10) interact via their extracellular regions: EVIDENCE FOR DISTINCT BINDING MECHANISMS FOR DIFFERENT TspanC8 PROTEINS. J Biol Chem 291: 3145–3157 26668317 10.1074/jbc.M115.703058 PMC4751363

[emmm201809695-bib-0070] Oikonomidi I , Burbridge E , Cavadas M , Sullivan G , Collis B , Naegele H , Clancy D , Brezinova J , Hu T , Bileck A *et al* (2018) iTAP, a novel iRhom interactor, controls TNF secretion by policing the stability of iRhom/TACE. Elife 7: e35032 29897333 10.7554/eLife.35032 PMC6042963

[emmm201809695-bib-0071] Pan D , Rubin GM (1997) Kuzbanian controls proteolytic processing of Notch and mediates lateral inhibition during *Drosophila* and vertebrate neurogenesis. Cell 90: 271–280 9244301 10.1016/s0092-8674(00)80335-9

[emmm201809695-bib-0072] Paudel S , Kim YH , Huh MI , Kim SJ , Chang Y , Park YJ , Lee KW , Jung JC (2013) ADAM10 mediates N‐cadherin ectodomain shedding during retinal ganglion cell differentiation in primary cultured retinal cells from the developing chick retina. J Cell Biochem 114: 942–954 23129104 10.1002/jcb.24435

[emmm201809695-bib-0073] Pigoni M , Wanngren J , Kuhn PH , Munro KM , Gunnersen JM , Takeshima H , Feederle R , Voytyuk I , De Strooper B , Levasseur MD *et al* (2016) Seizure protein 6 and its homolog seizure 6‐like protein are physiological substrates of BACE1 in neurons. Mol Neurodegener 11: 67 27716410 10.1186/s13024-016-0134-z PMC5053352

[emmm201809695-bib-0074] Postina R , Schroeder A , Dewachter I , Bohl J , Schmitt U , Kojro E , Prinzen C , Endres K , Hiemke C , Blessing M *et al* (2004) A disintegrin‐metalloproteinase prevents amyloid plaque formation and hippocampal defects in an Alzheimer disease mouse model. J Clin Invest 113: 1456–1464 15146243 10.1172/JCI20864 PMC406531

[emmm201809695-bib-0075] Postina R (2012) Activation of alpha‐secretase cleavage. J Neurochem 120(Suppl 1): 46–54 10.1111/j.1471-4159.2011.07459.x 21883223

[emmm201809695-bib-0076] Prinzen C , Trumbach D , Wurst W , Endres K , Postina R , Fahrenholz F (2009) Differential gene expression in ADAM10 and mutant ADAM10 transgenic mice. BMC Genom 10: 66 10.1186/1471-2164-10-66 PMC2647556 19196476

[emmm201809695-bib-0077] Prox J , Willenbrock M , Weber S , Lehmann T , Schmidt‐Arras D , Schwanbeck R , Saftig P , Schwake M (2012) Tetraspanin15 regulates cellular trafficking and activity of the ectodomain sheddase ADAM10. Cell Mol Life Sci 69: 2919–2932 22446748 10.1007/s00018-012-0960-2 PMC11114675

[emmm201809695-bib-0078] Prox J , Bernreuther C , Altmeppen H , Grendel J , Glatzel M , D'Hooge R , Stroobants S , Ahmed T , Balschun D , Willem M *et al* (2013) Postnatal disruption of the disintegrin/metalloproteinase ADAM10 in brain causes epileptic seizures, learning deficits, altered spine morphology, and defective synaptic functions. J Neurosci 33: 12915–12928, 12928a 23926248 10.1523/JNEUROSCI.5910-12.2013 PMC6619719

[emmm201809695-bib-0079] Rappsilber J , Ishihama Y , Mann M (2003) Stop and go extraction tips for matrix‐assisted laser desorption/ionization, nanoelectrospray, and LC/MS sample pretreatment in proteomics. Anal Chem 75: 663–670 12585499 10.1021/ac026117i

[emmm201809695-bib-0080] Reinhardt S , Grimm MO , Stahlmann C , Hartmann T , Shudo K , Tomita T , Endres K (2016) Rescue of hypovitaminosis a induces non‐amyloidogenic amyloid precursor protein (APP) processing. Curr Alzheimer Res 13: 1277–1289 27335034 10.2174/1567205013666160603002105

[emmm201809695-bib-0081] Reiss K , Maretzky T , Ludwig A , Tousseyn T , de Strooper B , Hartmann D , Saftig P (2005) ADAM10 cleavage of N‐cadherin and regulation of cell‐cell adhesion and beta‐catenin nuclear signalling. EMBO J 24: 742–752 15692570 10.1038/sj.emboj.7600548 PMC549617

[emmm201809695-bib-0082] Saftig P , Lichtenthaler SF (2015) The alpha secretase ADAM10: a metalloprotease with multiple functions in the brain. Prog Neurobiol 135: 1–20 26522965 10.1016/j.pneurobio.2015.10.003

[emmm201809695-bib-0083] Sahin U , Weskamp G , Kelly K , Zhou HM , Higashiyama S , Peschon J , Hartmann D , Saftig P , Blobel CP (2004) Distinct roles for ADAM10 and ADAM17 in ectodomain shedding of six EGFR ligands. J Cell Biol 164: 769–779 14993236 10.1083/jcb.200307137 PMC2172154

[emmm201809695-bib-0084] Sakry D , Neitz A , Singh J , Frischknecht R , Marongiu D , Biname F , Perera SS , Endres K , Lutz B , Radyushkin K *et al* (2014) Oligodendrocyte precursor cells modulate the neuronal network by activity‐dependent ectodomain cleavage of glial NG2. PLoS Biol 12: e1001993 25387269 10.1371/journal.pbio.1001993 PMC4227637

[emmm201809695-bib-0085] Sakurai T , Lustig M , Nativ M , Hemperly JJ , Schlessinger J , Peles E , Grumet M (1997) Induction of neurite outgrowth through contactin and Nr‐CAM by extracellular regions of glial receptor tyrosine phosphatase beta. J Cell Biol 136: 907–918 9049255 10.1083/jcb.136.4.907 PMC2132488

[emmm201809695-bib-0086] Sanchez‐Irizarry C , Carpenter AC , Weng AP , Pear WS , Aster JC , Blacklow SC (2004) Notch subunit heterodimerization and prevention of ligand‐independent proteolytic activation depend, respectively, on a novel domain and the LNR repeats. Mol Cell Biol 24: 9265–9273 15485896 10.1128/MCB.24.21.9265-9273.2004 PMC522238

[emmm201809695-bib-0087] Sanz RL , Ferraro GB , Girouard MP , Fournier AE (2017) Ectodomain shedding of Limbic System‐Associated Membrane Protein (LSAMP) by ADAM Metallopeptidases promotes neurite outgrowth in DRG neurons. Sci Rep 7: 7961 28801670 10.1038/s41598-017-08315-0 PMC5554145

[emmm201809695-bib-0088] Schafer MKE , Tegeder I (2018) NG2/CSPG4 and progranulin in the posttraumatic glial scar. Matrix Biol 68–69: 571–588 10.1016/j.matbio.2017.10.002 29054751

[emmm201809695-bib-0089] Scheltens P , Blennow K , Breteler MMB , de Strooper B , Frisoni GB , Salloway S , Van der Flier WM (2016) Alzheimer's disease. Lancet 388: 505–517 26921134 10.1016/S0140-6736(15)01124-1

[emmm201809695-bib-0090] Schlondorff J , Becherer JD , Blobel CP (2000) Intracellular maturation and localization of the tumour necrosis factor alpha convertase (TACE). Biochem J 347(Pt 1): 131–138 10727411 PMC1220940

[emmm201809695-bib-0091] Seipold L , Altmeppen H , Koudelka T , Tholey A , Kasparek P , Sedlacek R , Schweizer M , Bar J , Mikhaylova M , Glatzel M *et al* (2018) *In vivo* regulation of the A disintegrin and metalloproteinase 10 (ADAM10) by the tetraspanin 15. Cell Mol Life Sci 75: 3251–3267 29520422 10.1007/s00018-018-2791-2 PMC11105247

[emmm201809695-bib-0092] Selkoe DJ , Hardy J (2016) The amyloid hypothesis of Alzheimer's disease at 25 years. EMBO Mol Med 8: 595–608 27025652 10.15252/emmm.201606210 PMC4888851

[emmm201809695-bib-0093] Smathers RL , Petersen DR (2011) The human fatty acid‐binding protein family: evolutionary divergences and functions. Hum Genomics 5: 170–191 21504868 10.1186/1479-7364-5-3-170 PMC3500171

[emmm201809695-bib-0094] Stoeckli ET , Landmesser LT (1995) Axonin‐1, Nr‐CAM, and Ng‐CAM play different roles in the *in vivo* guidance of chick commissural neurons. Neuron 14: 1165–1179 7541632 10.1016/0896-6273(95)90264-3

[emmm201809695-bib-0095] Stoeckli ET , Sonderegger P , Pollerberg GE , Landmesser LT (1997) Interference with axonin‐1 and NrCAM interactions unmasks a floor‐plate activity inhibitory for commissural axons. Neuron 18: 209–221 9052792 10.1016/s0896-6273(00)80262-7

[emmm201809695-bib-0096] Strilic B , Yang L , Albarran‐Juarez J , Wachsmuth L , Han K , Muller UC , Pasparakis M , Offermanns S (2016) Tumour‐cell‐induced endothelial cell necroptosis via death receptor 6 promotes metastasis. Nature 536: 215–218 27487218 10.1038/nature19076

[emmm201809695-bib-0097] Susuki K , Chang KJ , Zollinger DR , Liu Y , Ogawa Y , Eshed‐Eisenbach Y , Dours‐Zimmermann MT , Oses‐Prieto JA , Burlingame AL , Seidenbecher CI *et al* (2013) Three mechanisms assemble central nervous system nodes of Ranvier. Neuron 78: 469–482 23664614 10.1016/j.neuron.2013.03.005 PMC3756512

[emmm201809695-bib-0098] Suzuki K , Hayashi Y , Nakahara S , Kumazaki H , Prox J , Horiuchi K , Zeng M , Tanimura S , Nishiyama Y , Osawa S *et al* (2012) Activity‐dependent proteolytic cleavage of neuroligin‐1. Neuron 76: 410–422 23083742 10.1016/j.neuron.2012.10.003

[emmm201809695-bib-0099] Tarabykina S , Mollerup J , Winding P , Berchtold MW (2004) ALG‐2, a multifunctional calcium binding protein? Front Biosci 9: 1817–1832 14977589 10.2741/1358

[emmm201809695-bib-0100] van Tetering G , van Diest P , Verlaan I , van der Wall E , Kopan R , Vooijs M (2009) Metalloprotease ADAM10 is required for Notch1 site 2 cleavage. J Biol Chem 284: 31018–31027 19726682 10.1074/jbc.M109.006775 PMC2781502

[emmm201809695-bib-0101] Tippmann F , Hundt J , Schneider A , Endres K , Fahrenholz F (2009) Up‐regulation of the alpha‐secretase ADAM10 by retinoic acid receptors and acitretin. FASEB J 23: 1643–1654 19144697 10.1096/fj.08-121392

[emmm201809695-bib-0102] Torre ER , Gutekunst CA , Gross RE (2010) Expression by midbrain dopamine neurons of Sema3A and 3F receptors is associated with chemorepulsion *in vitro* but a mild *in vivo* phenotype. Mol Cell Neurosci 44: 135–153 20298787 10.1016/j.mcn.2010.03.003 PMC2862895

[emmm201809695-bib-0103] Vadivelu S , Stewart TJ , Qu Y , Horn K , Liu S , Li Q , Silver J , McDonald JW (2015) NG2+ progenitors derived from embryonic stem cells penetrate glial scar and promote axonal outgrowth into white matter after spinal cord injury. Stem Cells Transl Med 4: 401–411 25713464 10.5966/sctm.2014-0107 PMC4367502

[emmm201809695-bib-0104] Wan XZ , Li B , Li YC , Yang XL , Zhang W , Zhong L , Tang SJ (2012) Activation of NMDA receptors upregulates a disintegrin and metalloproteinase 10 via a Wnt/MAPK signaling pathway. J Neurosci 32: 3910–3916 22423111 10.1523/JNEUROSCI.3916-11.2012 PMC6703469

[emmm201809695-bib-0105] Wildsmith KR , Schauer SP , Smith AM , Arnott D , Zhu Y , Haznedar J , Kaur S , Mathews WR , Honigberg LA (2014) Identification of longitudinally dynamic biomarkers in Alzheimer's disease cerebrospinal fluid by targeted proteomics. Mol Neurodegener 9: 22 24902845 10.1186/1750-1326-9-22 PMC4061120

[emmm201809695-bib-0106] Yu S , Levi L , Siegel R , Noy N (2012) Retinoic acid induces neurogenesis by activating both retinoic acid receptors (RARs) and peroxisome proliferator‐activated receptor beta/delta (PPARbeta/delta). J Biol Chem 287: 42195–42205 23105114 10.1074/jbc.M112.410381 PMC3516764

[emmm201809695-bib-0107] Zelina P , Avci HX , Thelen K , Pollerberg GE (2005) The cell adhesion molecule NrCAM is crucial for growth cone behaviour and pathfinding of retinal ganglion cell axons. Development 132: 3609–3618 16033798 10.1242/dev.01934

