# Supplementary material for: NrCAM is a marker for substrate‐selective activation of ADAM10 in Alzheimer's disease
Source: EMBO Mol Med. 2019 Mar 4;11(4):e9695. doi: 10.15252/emmm.201809695 (PMC6460357; doi:10.15252/emmm.201809695)
Supplement: Supplementary file 1 — Expanded View Figures PDF [file EMMM-11-e9695-s001.pdf]

Expanded View Figures

Figure EV1. NrCAM is neither a constitutive nor a stimulated ADAM17 substrate.

A Lysates (lys.) and conditioned media (med.) from wt and NrCAM knock-down neurons, both treated with either GI254023X (5 μM) or solvent, were analyzed next to each other. The C-terminal antibody only recognized proNrCAM, while the N-terminal NrCAM antibody detected both mNrCAM, proNrCAM, and sNrCAM in the conditioned media. * indicates an unspecific band.

B ADAM17fl/fl neurons were treated with an iCre virus, inducing the ADAM17 knock-out, or a control lentivirus at DIV2. The cells were kept in culture until DIV5; then, the neurons were treated with the ADAM10-preferring inhibitor GI254023x (5 μM) or solvent (control). After 24 h, the cells were lysed and the conditioned media were collected. * indicates an unspecific band; < indicates the band of mADAM17.

C Wt neurons were kept in culture until DIV5; then, the cells were treated for 3 h with PMA (1 μM) alone, PMA (1 μM) and GI254023x (5 μM), or solvent.

D ADAM17fl/fl neurons were prepared like in (B). At DIV5, the neurons were treated with PMA (1 μM) alone, PMA (1 μM) and GI254023x (5 μM), or solvent for 3 h. In contrast to other ADAM10 substrates (e.g., L1CAM, APP), the phorbol ester PMA did not stimulate NrCAM cleavage.

Data information: In (B–D), densitometric quantifications of the Western blots are shown below. One-way ANOVA with *post hoc* Dunnett's test for (B and D), or two-sided Student's *t*-test for (C) (*$P < 0.05$; **$P < 0.01$; ****$P < 0.0001$, $n = 4$–6). Shown are mean and SEM. The mean levels of solvent-treated cells were set to 1. Representative Western blots are shown.
Source data are available online for this figure.

© 2019 The Authors

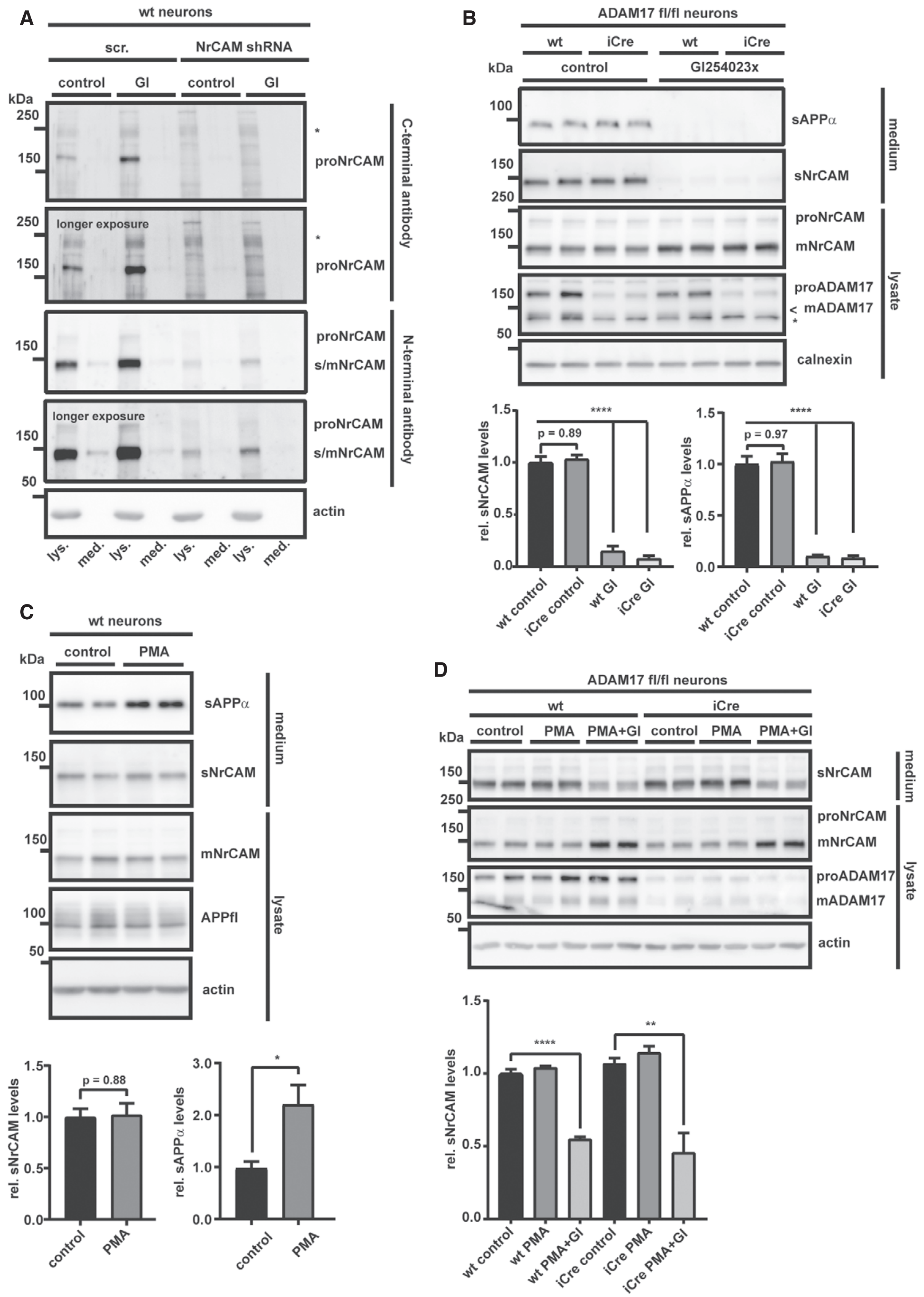

Figure EV1.

 © 2019 The Authors

Figure EV2. C- and N-terminal NrCAM fragments stay attached after initial furin cleavage.

A Co-immunoprecipitation of C- and N-terminal NrCAM fragments in lysates of HEK 293 cells transfected with a C-terminally VSV-tagged NrCAM construct, or empty vector. Cells were lysed in CoIP buffer. IP was performed with an N-terminal NrCAM antibody, detection with a C-terminal VSV antibody. The CTFf (CTFfurin) band was running a little bit higher than in the input control, presumably because of the abundant IgG heavy chain, running right beneath it. Because of the lack of a suitable antibody, we were not able to detect the small fragment between the furin and the ADAM10 cleavage site. * indicates the IgG heavy chain.

B Co-immunoprecipitation like in A, but the IP was done with a C-terminal VSV antibody and the detection with an N-terminal NrCAM antibody. NrCAM was detected as non-cleaved proNrCAM and the furin-cleaved mature mNrCAM.

C To test whether NrCAM was sequentially cleaved by furin and then ADAM10, we treated primary neurons with the furin inhibitor dec-CMK (50 μM), GI254023x (5 μM), or solvent for 48 h. Furin inhibition did not block NrCAM shedding, but caused the appearance of a 170 kDa band in the conditioned media, which was inhibited by GI254023x. In addition, dec-CMK increased the 220 kDa proNrCAM (but not mNrCAM), which was even further increased by GI254023x. The levels of total sNrCAM remained unaffected by the furin inhibition. Densitometric quantifications of the Western blots are shown. One-way ANOVA with *post hoc* Dunnett's test (****$P < 0.0001$, $n = 6$). Given are mean ± the standard error of the mean. The mean levels of solvent-treated cells were set to 1.

D Neurons were treated with dec-CMK or solvent like in (C). The small decrease in mADAM10 levels significantly reduced sAPPα, but not total sNrCAM release. Densitometric quantifications of the Western blots are shown. Two-sided Student's *t*-test (**$P < 0.01$; ***$P < 0.001$, $n = 4$). Given are mean ± the standard error of the mean. The mean levels of solvent-treated cells were set to 1. Together, these results show that ADAM10 is not only able to cleave both mNrCAM and proNrCAM, but is also the protease to release sNrCAM into the conditioned media. Thus, NrCAM is firstly cleaved by furin and then by ADAM10, releasing sNrCAM. Representative Western blots are shown.

Source data are available online for this figure.

© 2019 The Authors

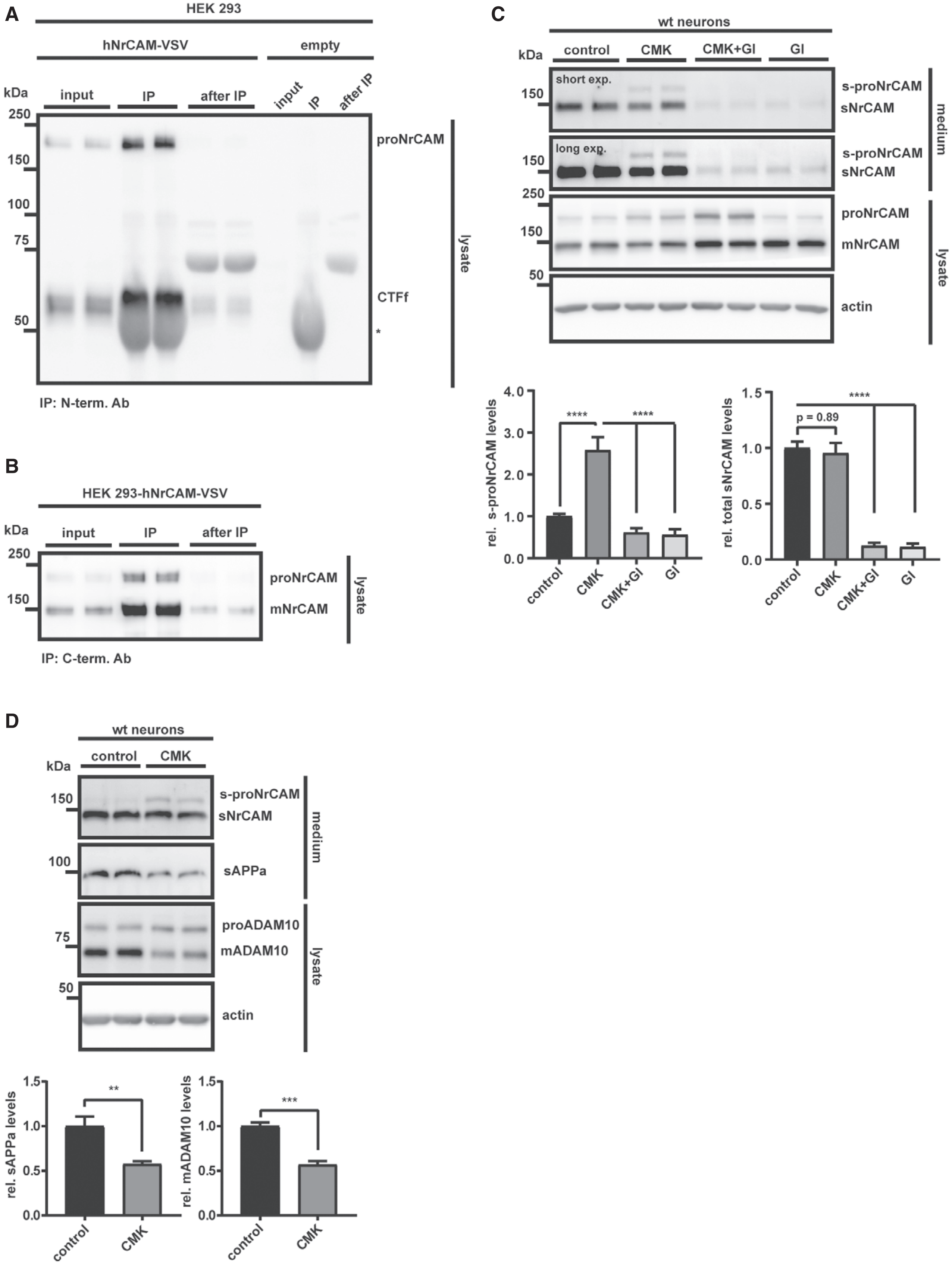

Figure EV2.

© 2019 The Authors

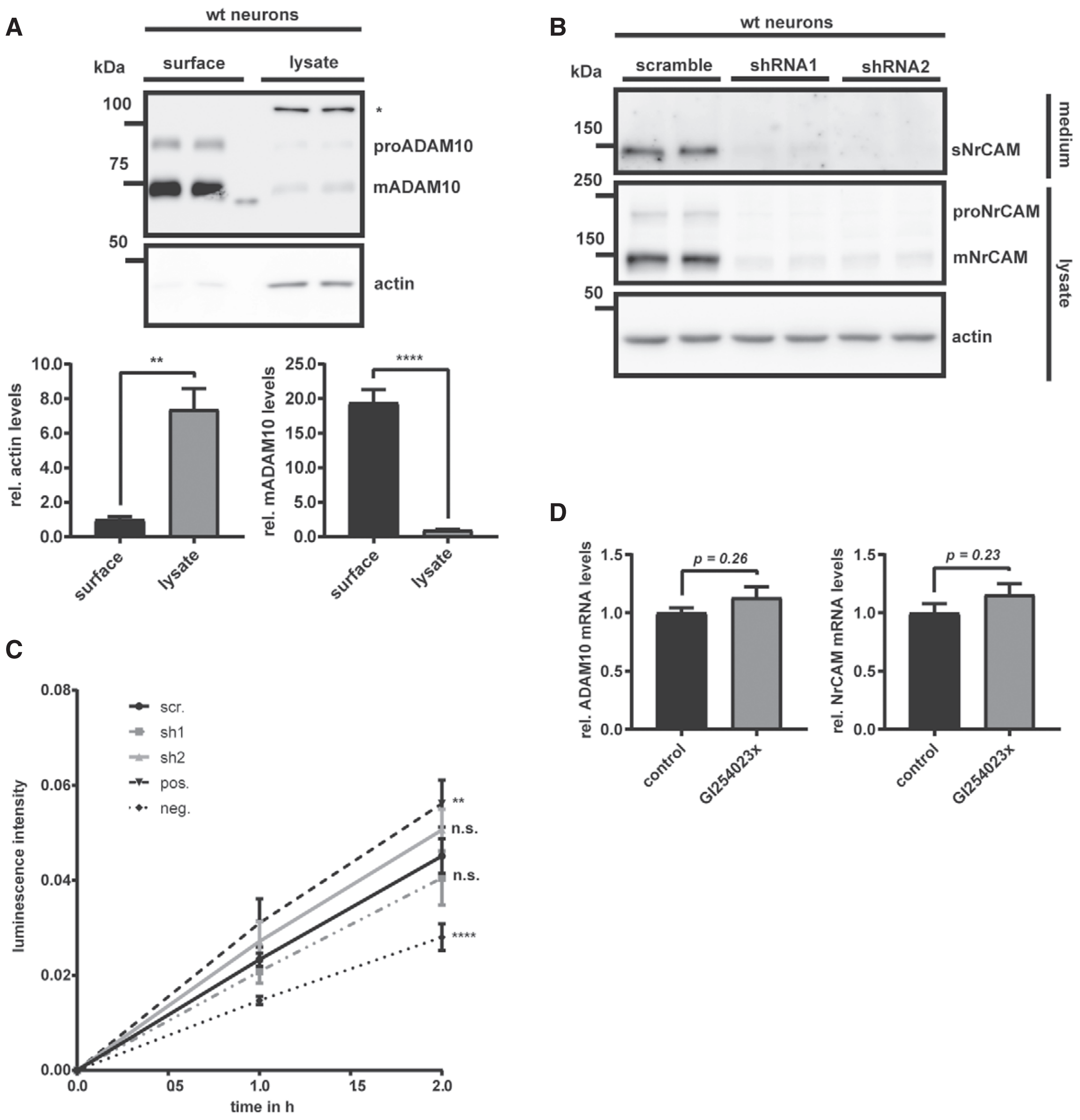

Figure EV3. Surface biotinylation efficiency and knock-down of NrCAM using shRNA-containing lentiviruses.

A To test the efficacy of our biotinylation assay, 100 μg of total protein was used for streptavidin pull-down, run next to 10 μg of total lysate, and were compared for the respective abundance of β-actin and ADAM10. Densitometric quantifications of the Western blots are shown. Two-sided Student's *t*-test (**$P < 0.01$; ****$P < 0.0001$, $n = 4$). Given are mean ± the standard error of the mean. * indicates an unspecific band. Surface and lysate samples are separated by a marker lane.

B Wt neurons were infected with NrCAM shRNA-containing lentiviruses (shRNA1 or shRNA2; 1:1,000), or a scrambled control construct (1:1,000). At DIV4, the cells were lysed and the conditioned media were collected.

C Cell viabilities after infection with the respective viruses (scr., sh1 and sh2) were compared to a positive (no toxic effect) and negative control (high toxicity) with Cell Counting Kit 8 (Sigma). The luminescence signals were measured with a microplate reader. Both shRNAs did not show higher toxicity than the scrambled control virus. Pos. = positive survival control (no treatment); neg. = negative survival control (cells treated with NaOH). One-way ANOVA with *post hoc* Dunnett's test ($^{n.s}P > 0.05$; **$P < 0.01$; ****$P < 0.0001$, $n = 8$). *P*-values were calculated by comparing the respective treatments to the treatment with the scr. shRNA. Shown are mean and SEM. Representative Western blots are shown.

D qPCRs for the ADAM10 and NrCAM mRNAs from wt neurons treated with GI254023x or solvent for 48 h (preparation and treatment of the cells were performed like in Fig 3B). Two-sided Student's *t*-test ($n = 5–6$). Given are mean ± the standard error of the mean.

Source data are available online for this figure.

© 2019 The Authors

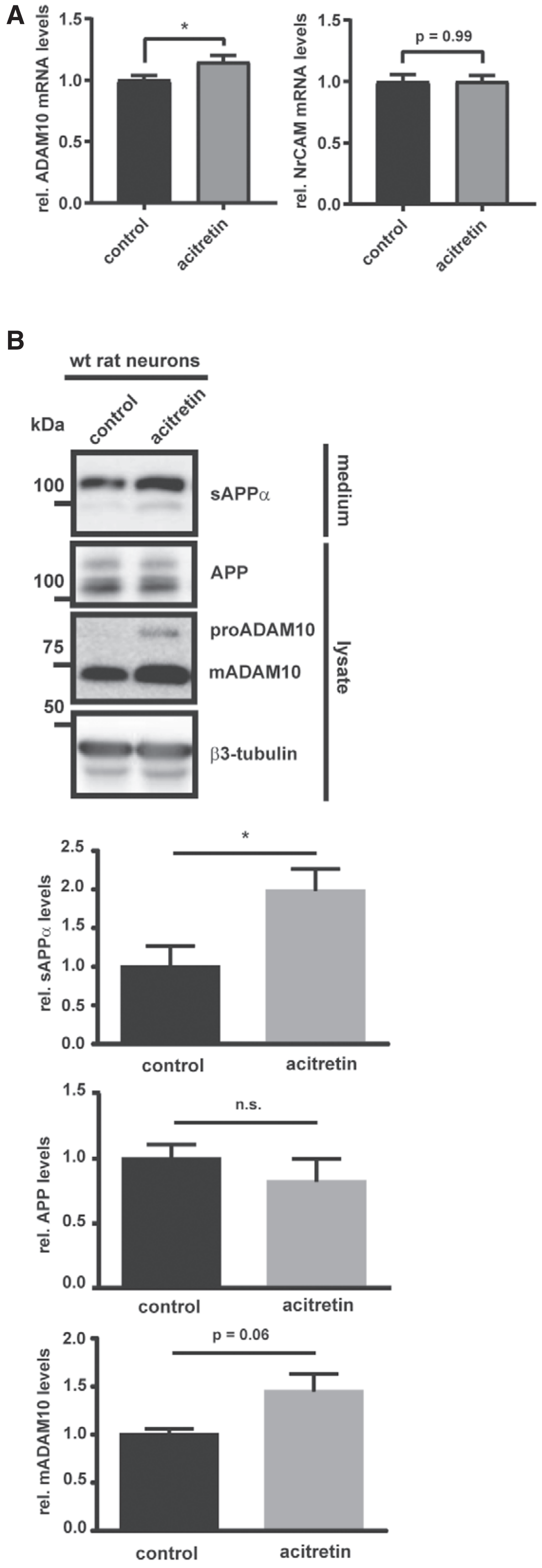

Figure EV4. Acitretin increases ADAM10 transcription in murine and ADAM10 and sAPPa levels in aged rat neurons.

A qPCRs for the ADAM10 and NrCAM mRNAs from wt neurons (DIV7) treated with acitretin (4 μM) or solvent for 48 h (preparation and treatment of the cells were performed like in Fig 4A). Two-sided Student's *t*-test ($^*P < 0.05$, $n = 15$). Given are mean ± the standard error of the mean.

B Cortical neurons were prepared from Wistar rat (embryonic day E18) and cultured for 21 days. Acitretin (2 μM) or solvent was added at DIV19. Two-sided Student's *t*-test ($^{n.s}P > 0.05$; $^*P < 0.05$, $n = 4$). Given are mean ± the standard error of the mean. Representative Western blots are shown.

Source data are available online for this figure.

© 2019 The Authors

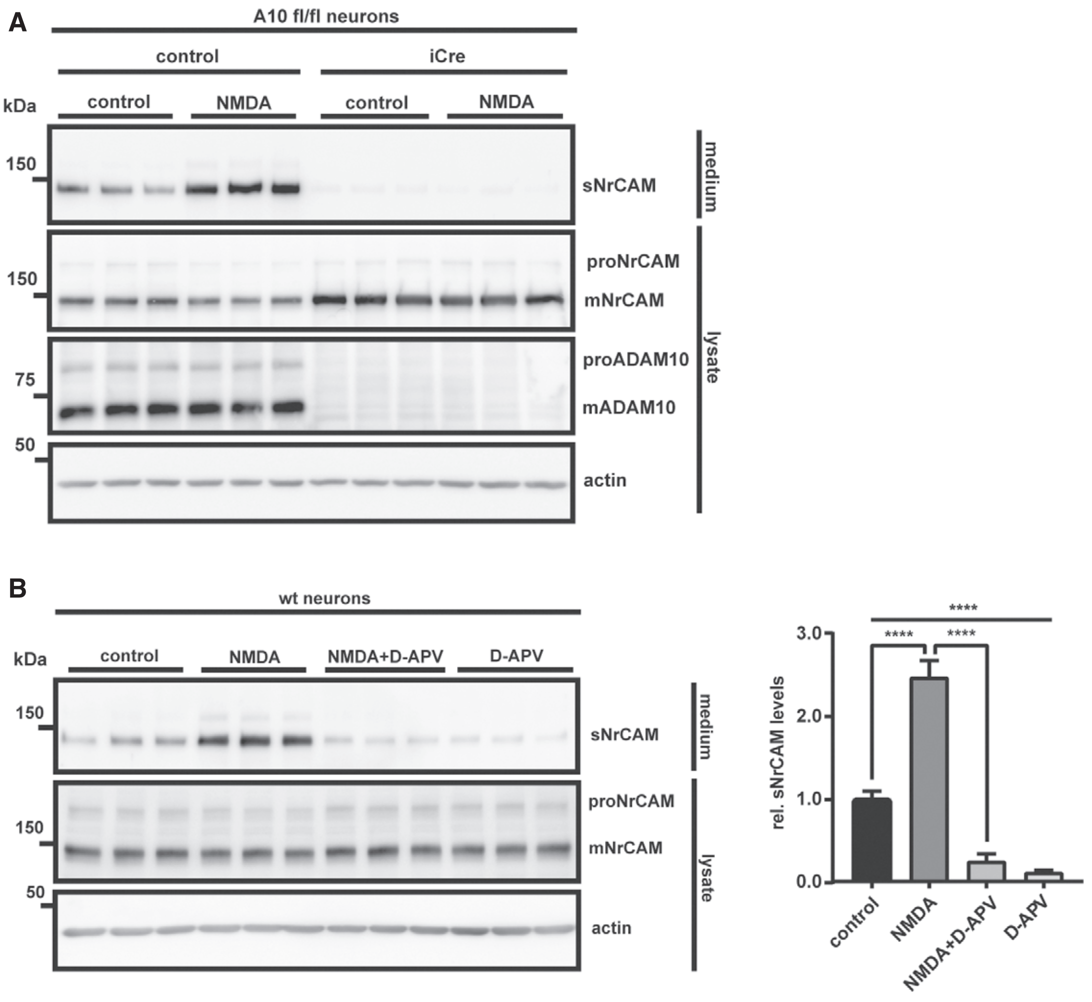

Figure EV5. Neuronal stimulation by NMDA increases ADAM10-mediated NrCAM shedding.

A To genetically validate our findings, ADAM10fl/fl neurons were treated with an iCre, or a control lentivirus at DIV2, to knock out ADAM10. The cells were kept in culture until DIV10; then, the neurons were treated with NMDA (50 μM) or vehicle for 30 min (Wan *et al*, 2012).

B Wt neurons were cultured like in (Fig 5A). At DIV10, the cells were pretreated with the NMDA receptor antagonist D-APV (100 μM) or vehicle for 30 min; then, the neurons were treated with NMDA (50 μM) or vehicle for 30 min. Densitometric quantifications of the Western blots are shown. One-way ANOVA with *post hoc* Dunnett's test (****$P < 0.0001$, $n = 6$). Shown are the mean and SEM. Representative Western blots are shown.

Source data are available online for this figure.

© 2019 The Authors
