# Supplementary material for: NrCAM is a marker for substrate‐selective activation of ADAM10 in Alzheimer's disease
Source: EMBO Mol Med. 2019 Mar 4;11(4):e9695. doi: 10.15252/emmm.201809695 (PMC6460357; doi:10.15252/emmm.201809695)
Supplement: Supplementary file 3 — Source Data for Expanded View [file EMMM-11-e9695-s009.zip › EV_source_data/Figure_EV4/Figure_EV4.docx]

**Figure EV4:**

**Western blots:**

The red dashed boxes show which representative part of the blot was chosen for the generation of the final figure.

Protein names are shown on the left side. Lys: lysate; sup: supernatant/conditioned media.

**Graph Pad prism files:**

Normal distribution was assumed.

Statistics were calculated for sAPPα, mADAM10, APP and mRNA of ADAM10 and NrCAM.

**Link for a free prism viewer:** https://www.graphpad.com/support/faqid/788/
