# Supplementary material for: NrCAM is a marker for substrate‐selective activation of ADAM10 in Alzheimer's disease
Source: EMBO Mol Med. 2019 Mar 4;11(4):e9695. doi: 10.15252/emmm.201809695 (PMC6460357; doi:10.15252/emmm.201809695)
Supplement: Supplementary file 3 — Source Data for Expanded View [file EMMM-11-e9695-s009.zip › EV_source_data/Figure_EV1/Figure_EV1.pdf]

Figure EV1

A

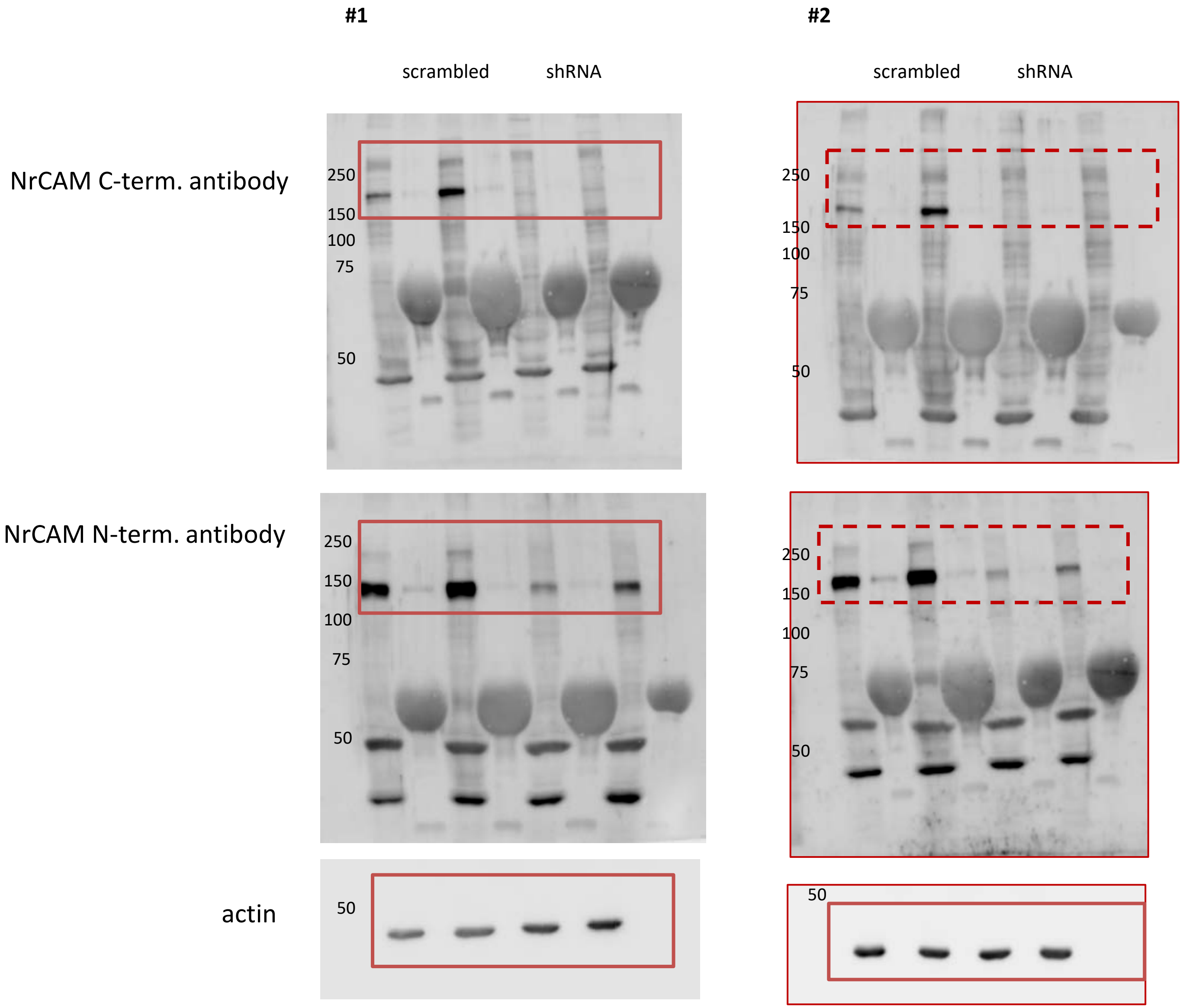
#1
#2
scrambled
shRNA
scrambled
shRNA
NrCAM C-term. antibody
250
150
100
75
50
250
150
100
75
50
NrCAM N-term. antibody
250
150
100
75
50
250
150
100
75
50
actin
50
50

B

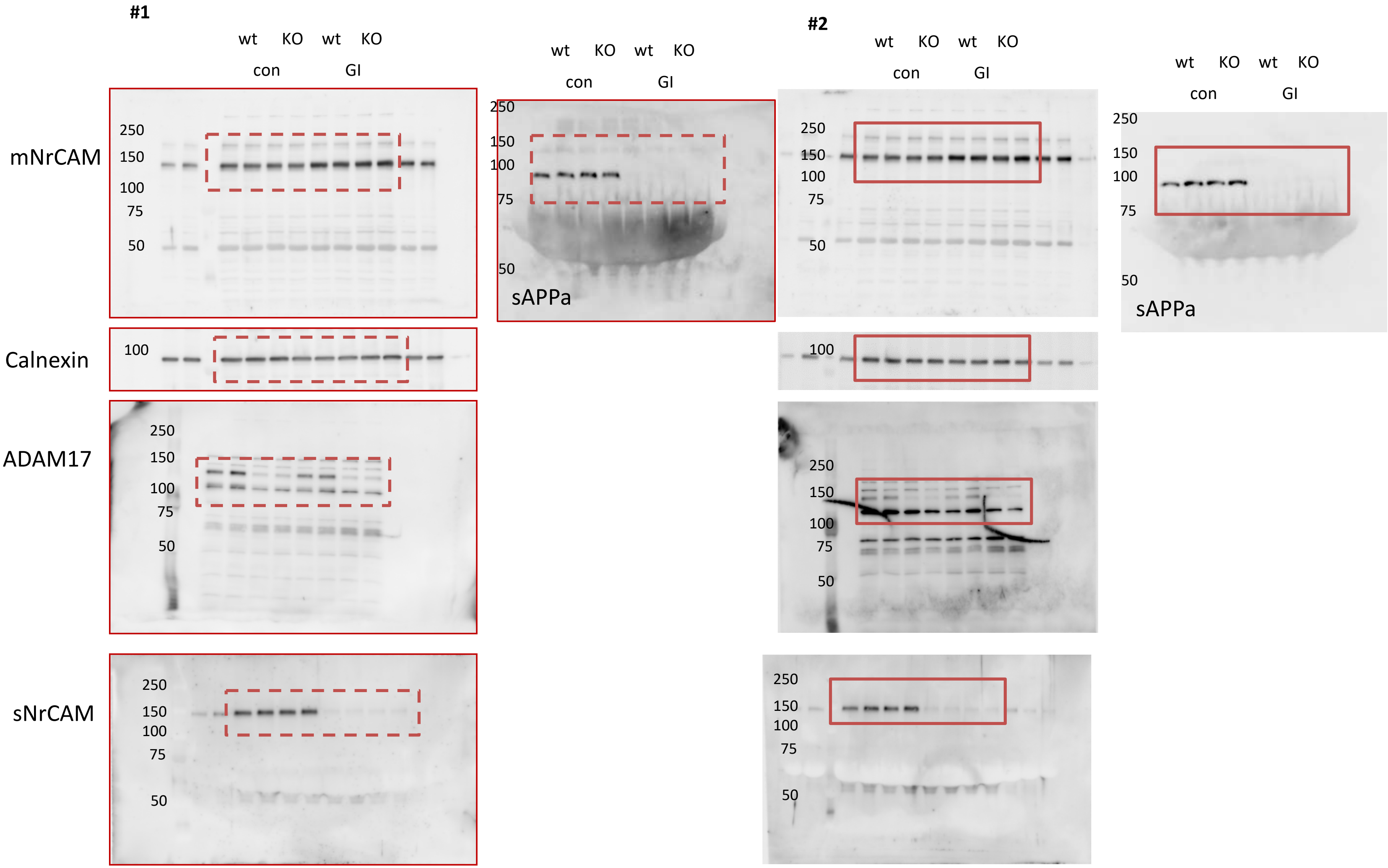
#1
wt KO wt KO
con GI
mNrCAM
250
150
100
75
50
Calnexin
100
ADAM17
250
150
100
75
50
sNrCAM
250
150
100
75
50
wt KO wt KO
con GI
250
150
100
75
50
sAPPa
#2
wt KO wt KO
con GI
250
150
100
75
50
100
250
150
100
75
50
250
150
100
75
50
wt KO wt KO
con GI
250
150
100
75
50
sAPPa

C conditioned medium

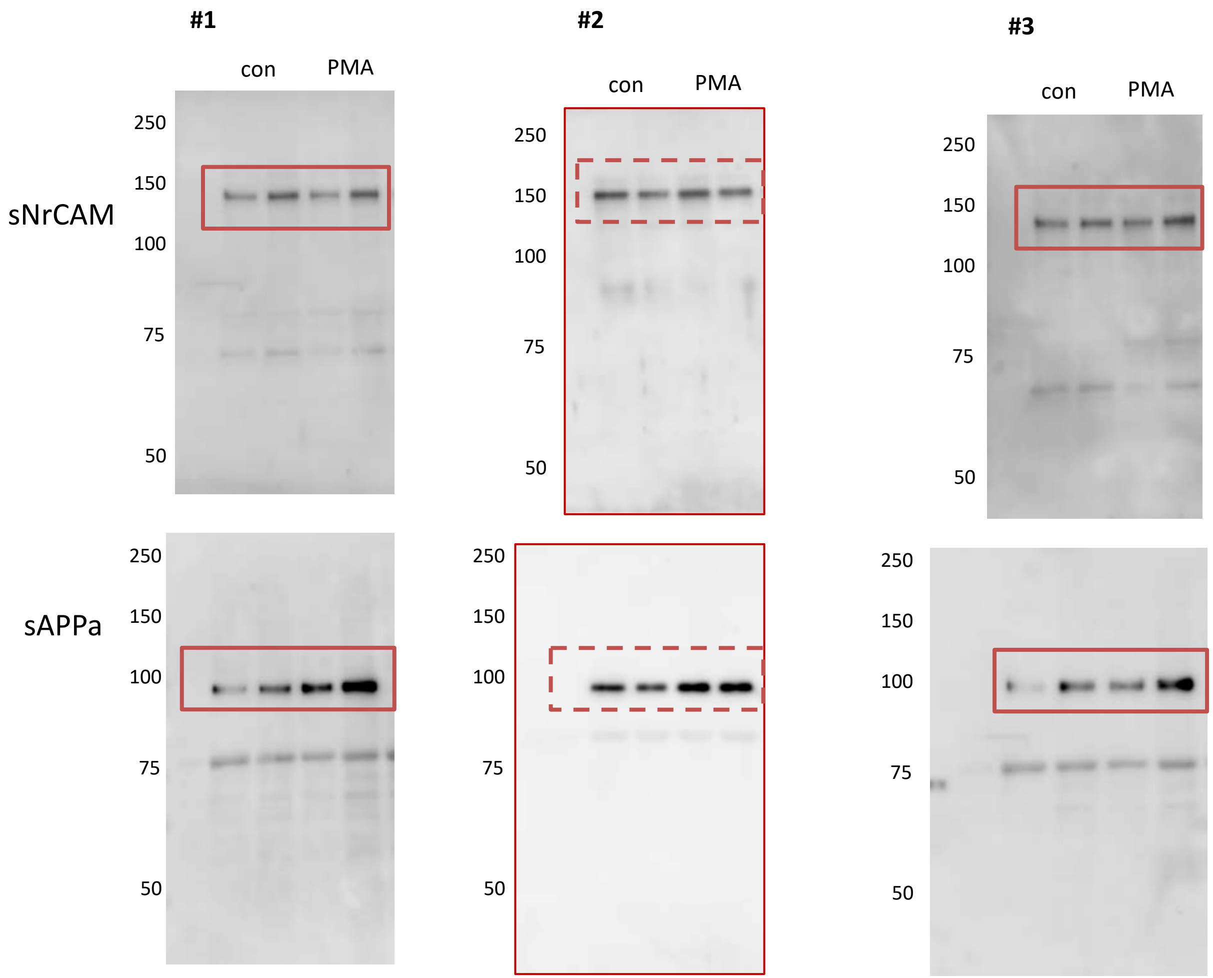

C lysate

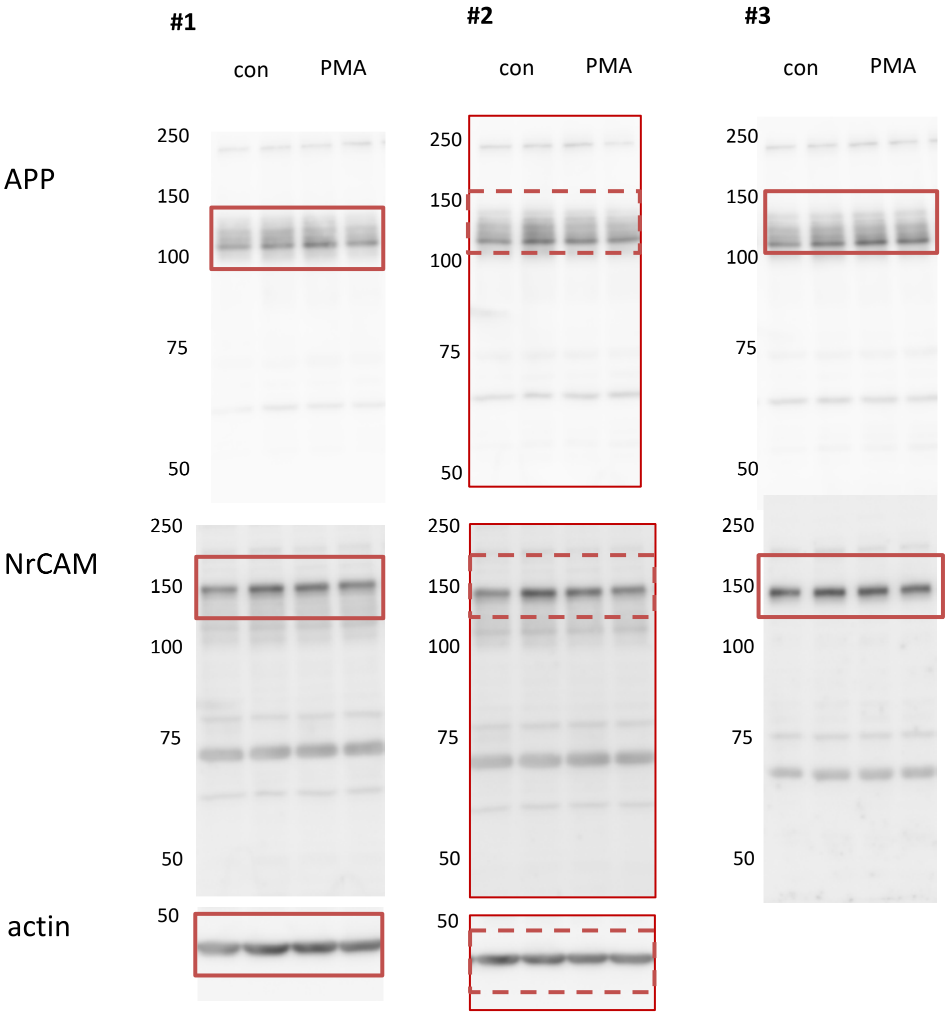
#1
#2
#3
con
PMA
con
PMA
con
PMA
APP
250
150
100
75
50
250
150
100
75
50
250
150
100
75
50
NrCAM
250
150
100
75
50
250
150
100
75
50
250
150
100
75
50
actin
50
50

D

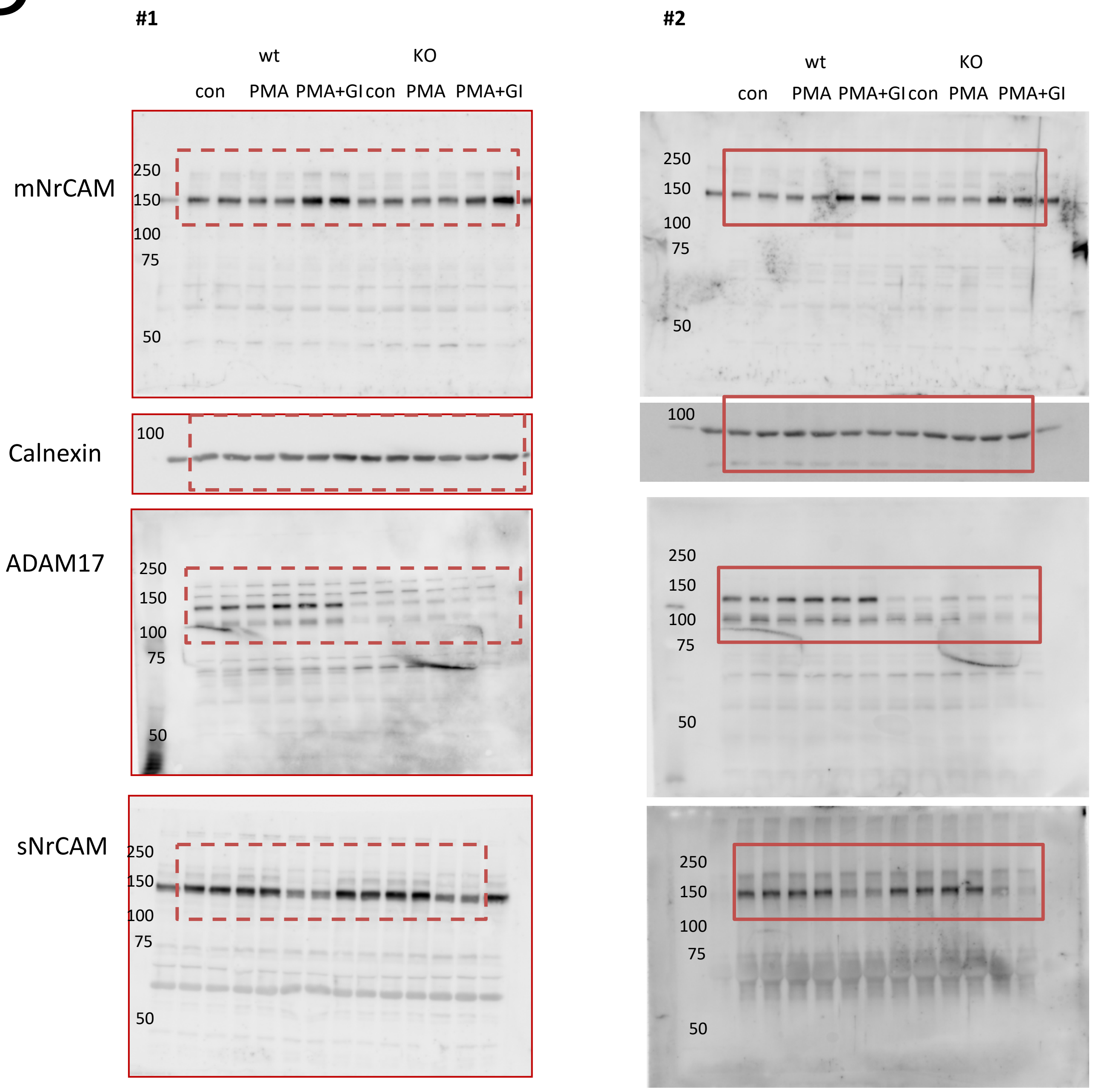

#1
#2
wt
KO
con
PMA
PMA+GI
mNrCAM
Calnexin
ADAM17
sNrCAM
250
150
100
75
50
