# Supplementary figures and images for: NrCAM is a marker for substrate‐selective activation of ADAM10 in Alzheimer's disease
Source: EMBO Mol Med. 2019 Mar 4;11(4):e9695. doi: 10.15252/emmm.201809695 (PMC6460357; doi:10.15252/emmm.201809695)

Figure EV4

B

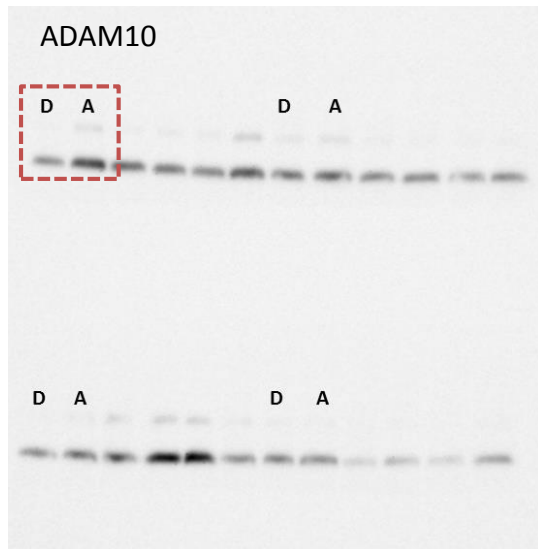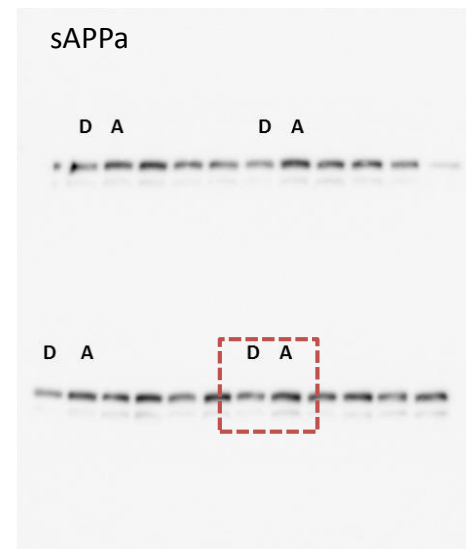

D: DMSO  
A: Acitretin

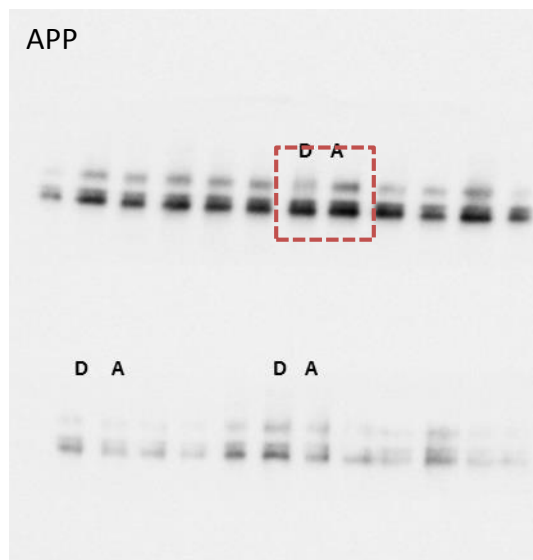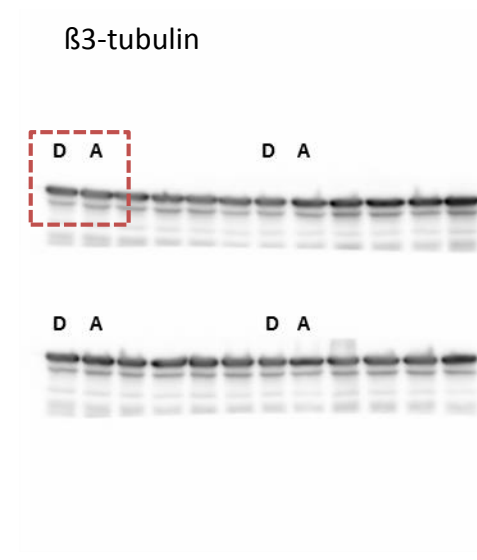

Supplement: Supplementary file 3 — Source Data for Expanded View [file EMMM-11-e9695-s009.zip › EV_source_data/Figure_EV4/Figure_EV4.pdf]

# Figure EV3

# A

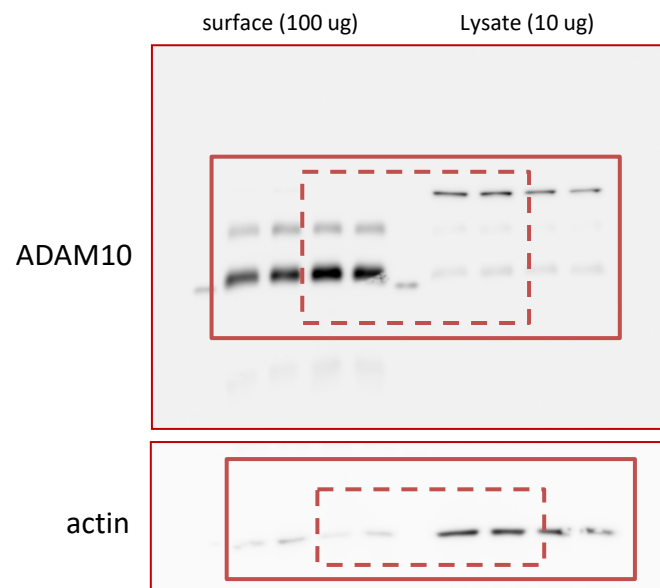

Replicates #1 and #2 were run on the same gel. Samples from 2 independent experiments.

# B

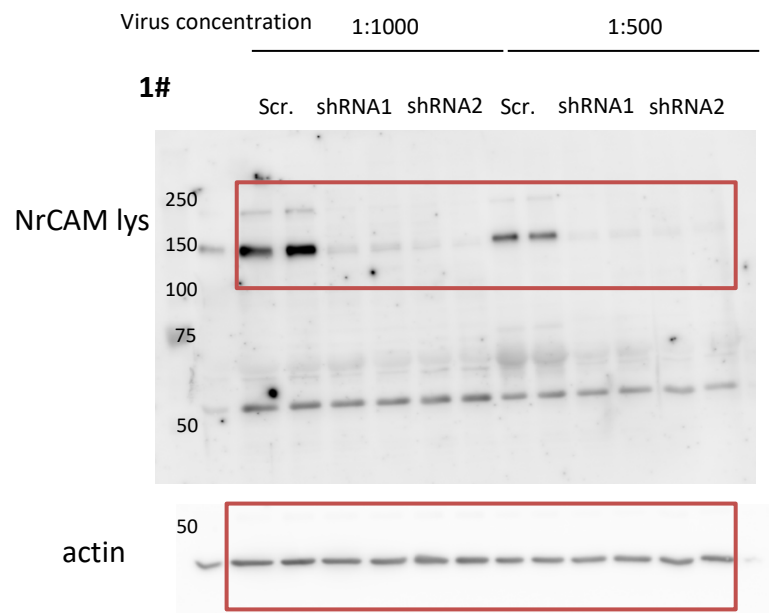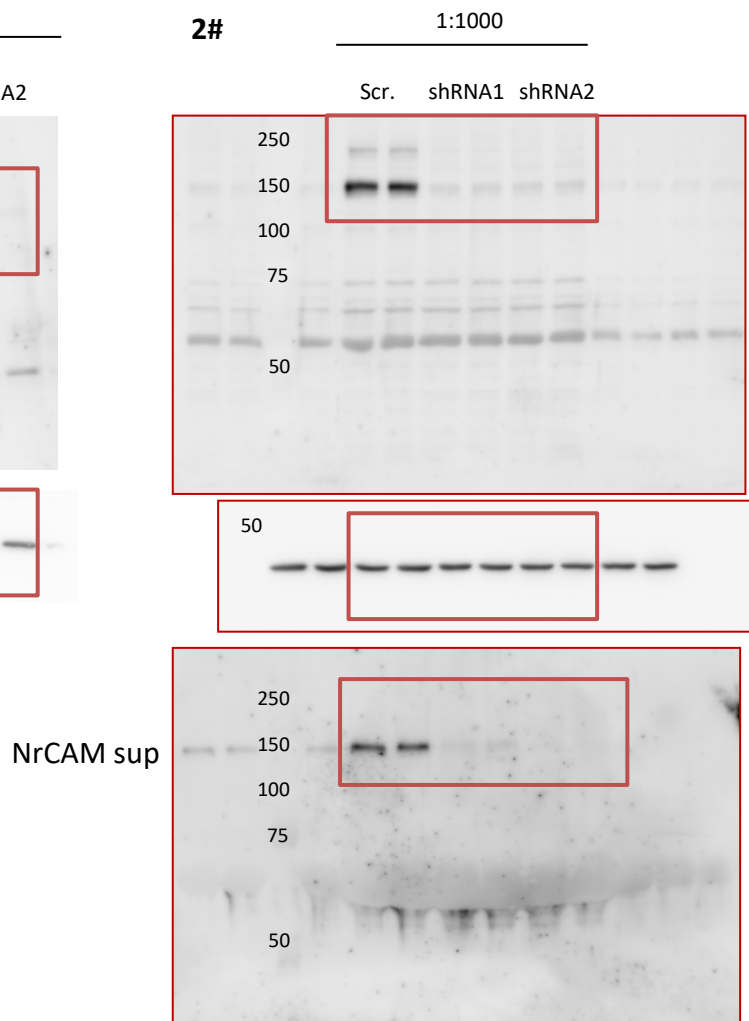

Supplement: Supplementary file 3 — Source Data for Expanded View [file EMMM-11-e9695-s009.zip › EV_source_data/Figure_EV3/Figure_EV3.pdf]

# Figure EV2

# A+B

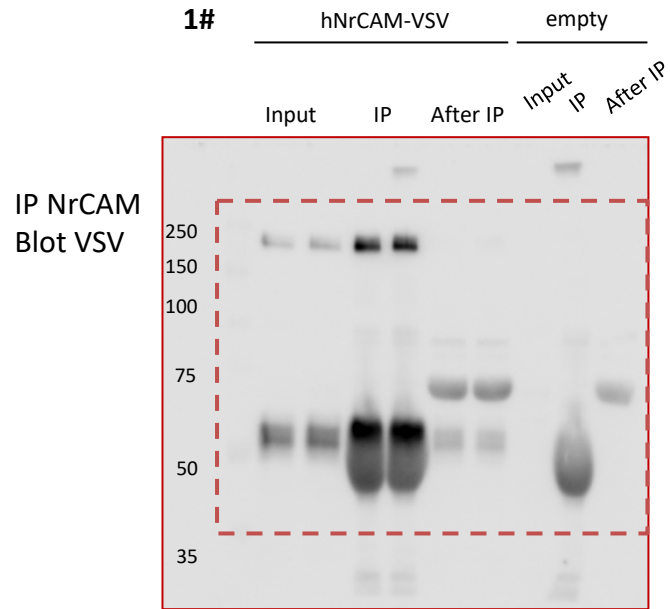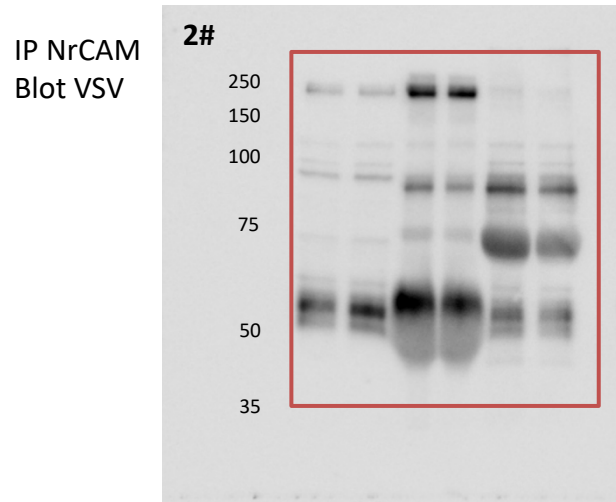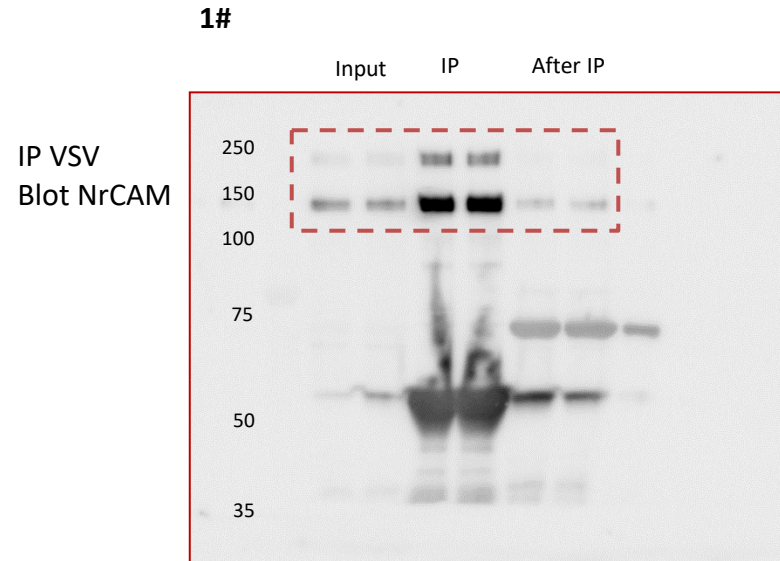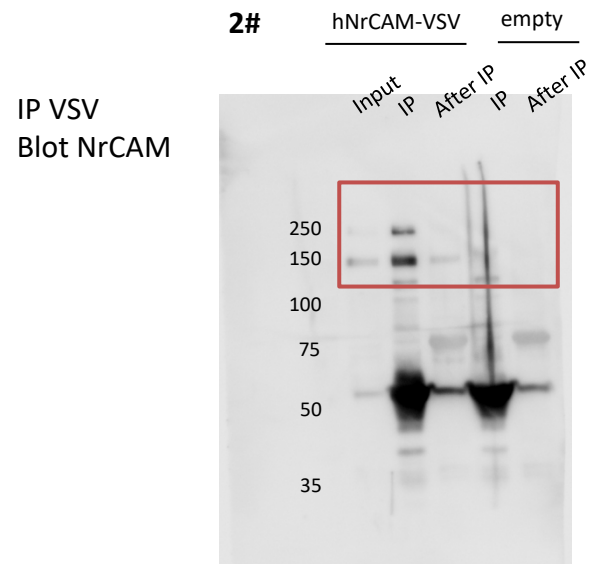

# C

## 1#

NrCAM lys

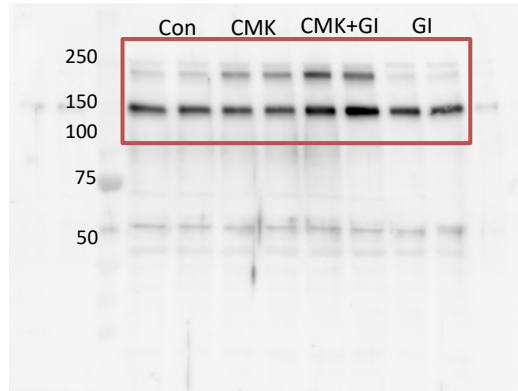

## 2#

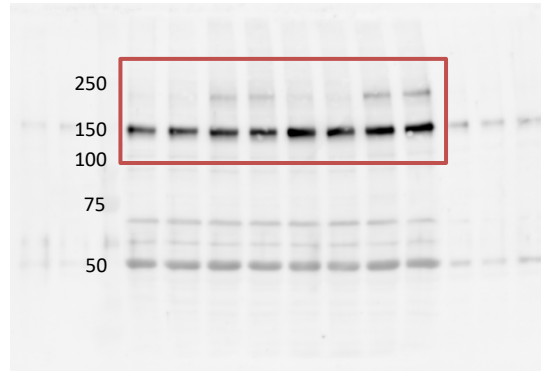

## 3#

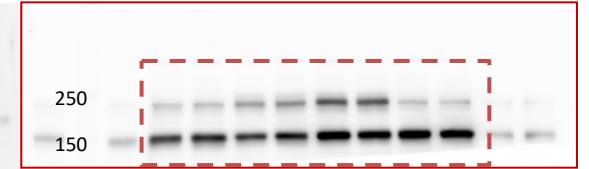

actin

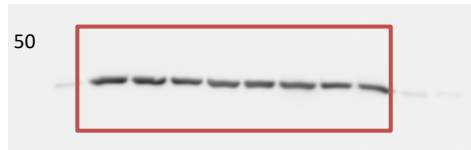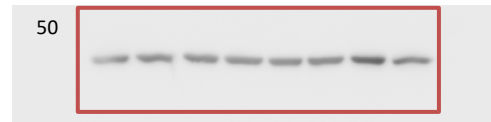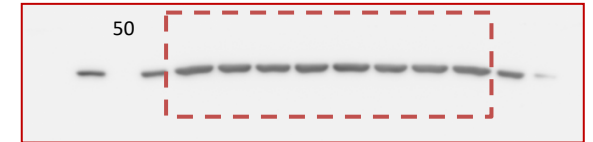

NrCAM sup

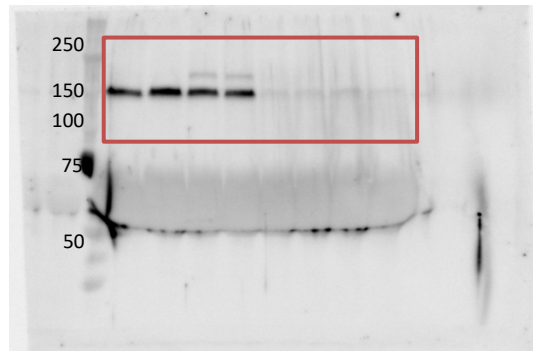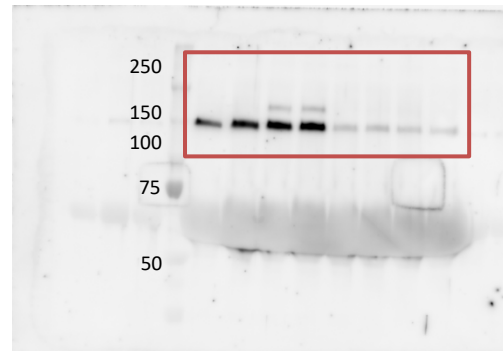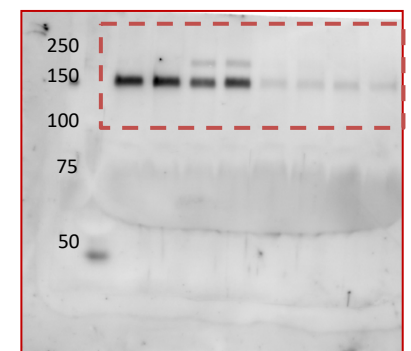

# D

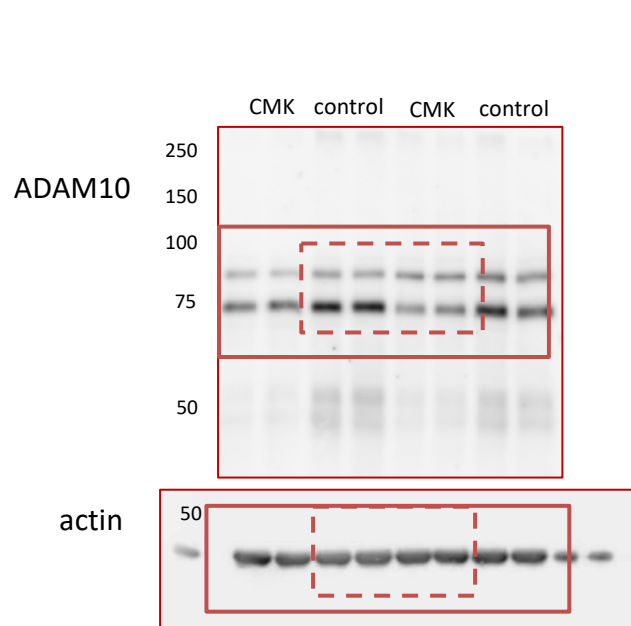

Replicates # 1 and #2 were run on the same gel

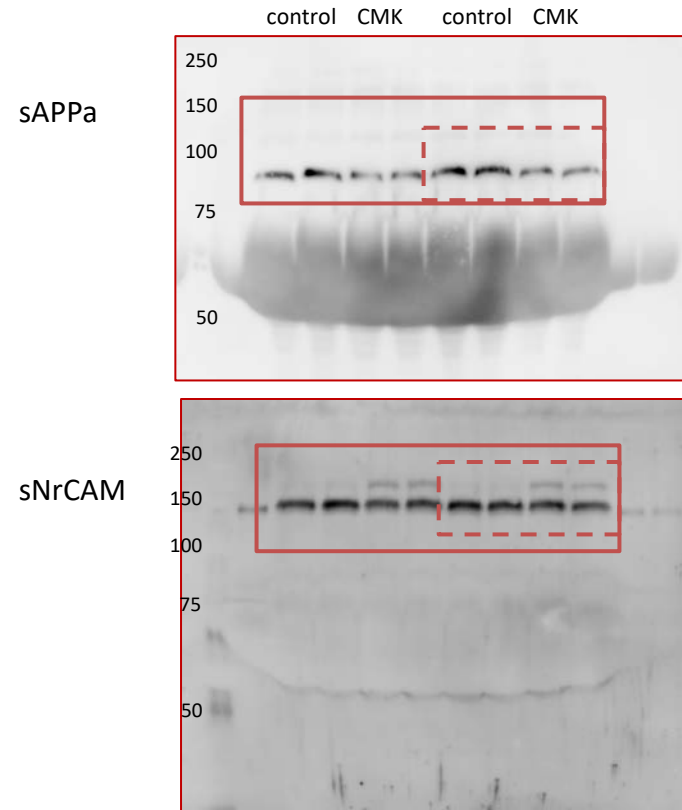

Supplement: Supplementary file 3 — Source Data for Expanded View [file EMMM-11-e9695-s009.zip › EV_source_data/Figure_EV2/Figure_EV2.pdf]

# Figure EV5

# A

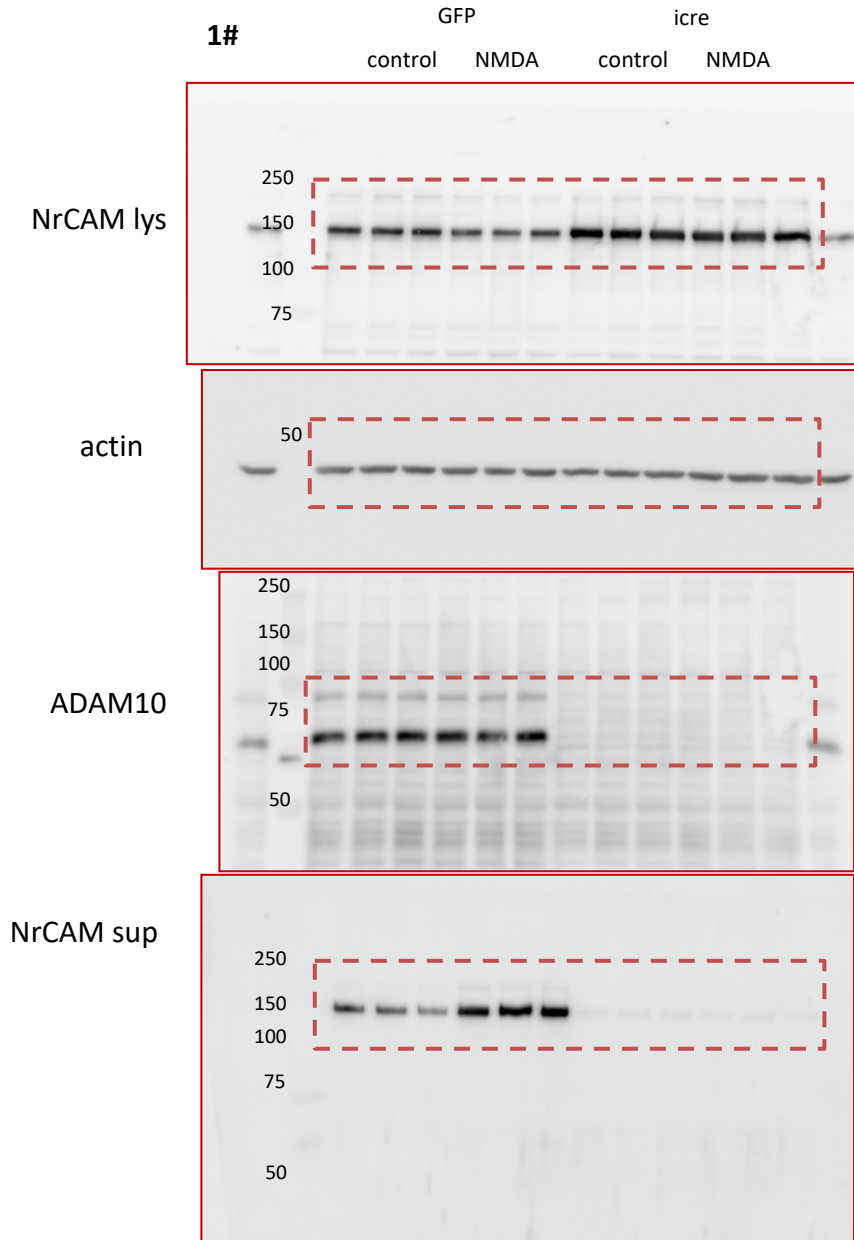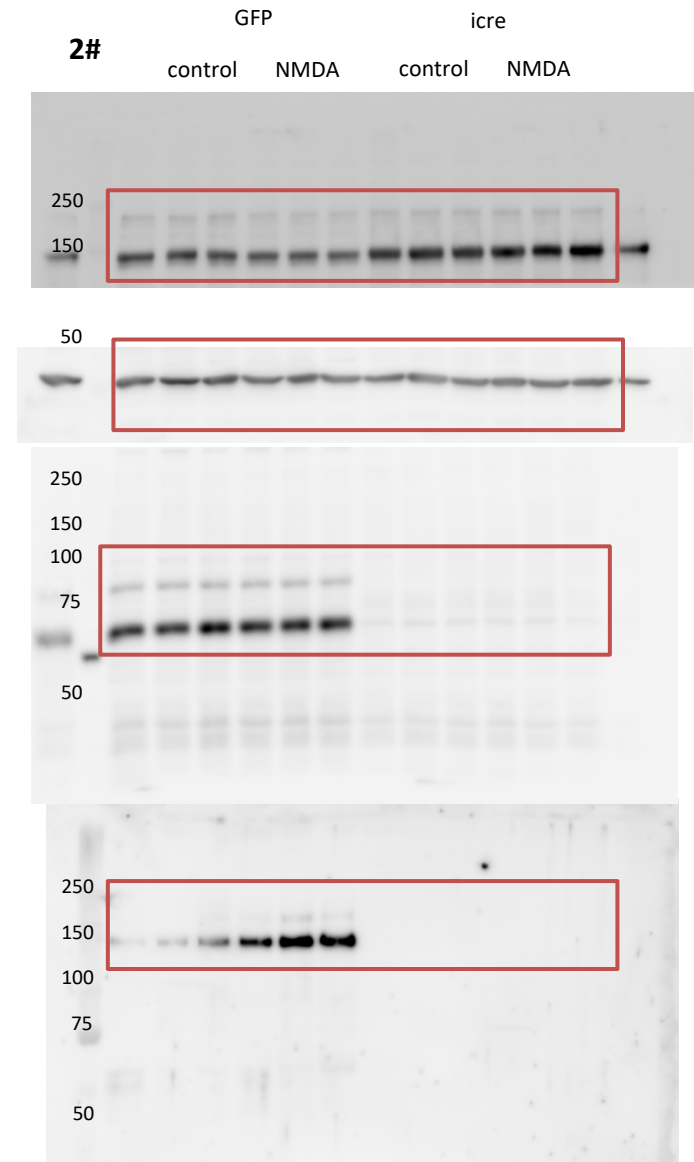

# B

## 1#

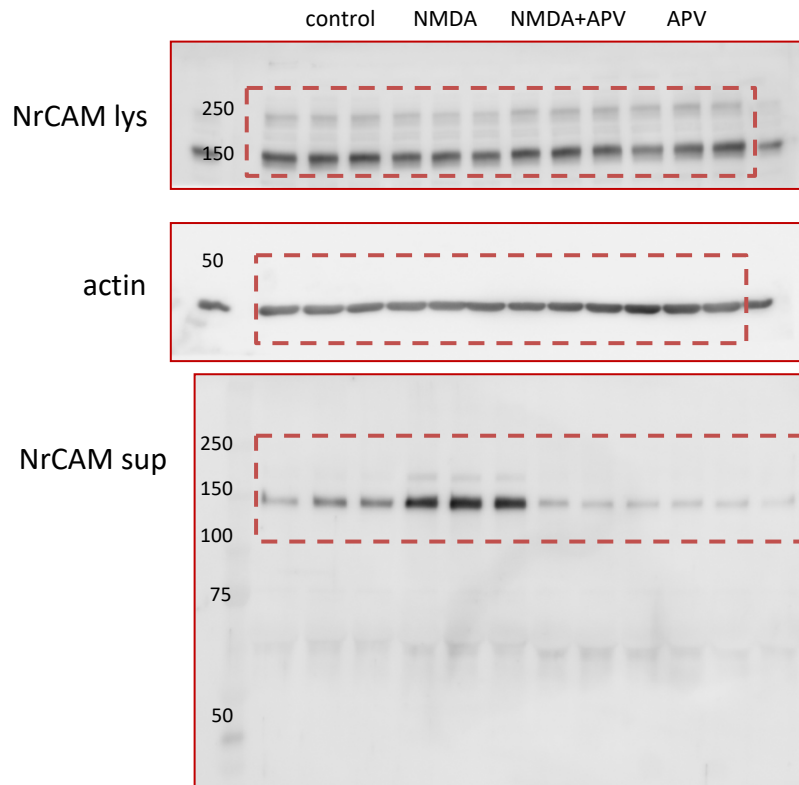

## 2#

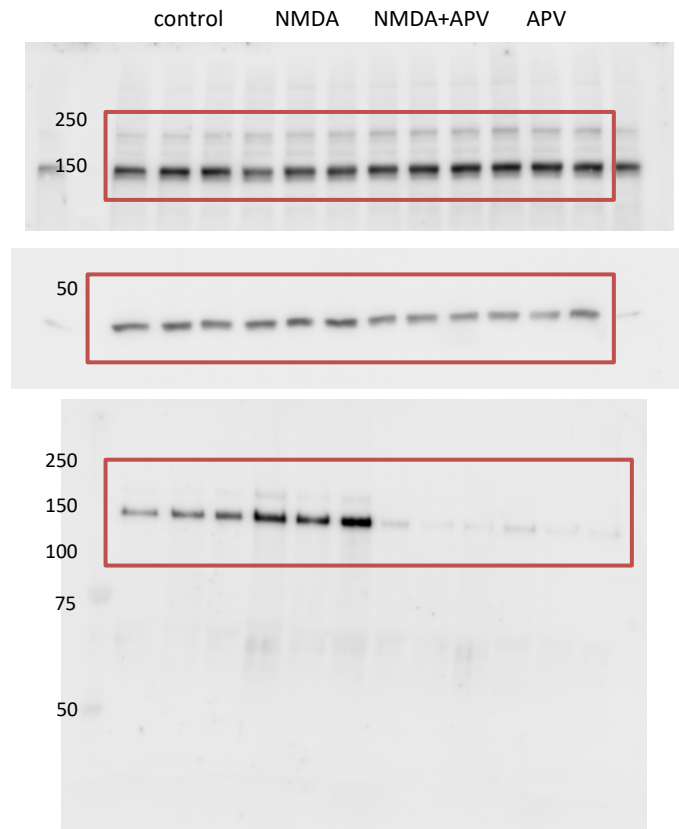

Supplement: Supplementary file 3 — Source Data for Expanded View [file EMMM-11-e9695-s009.zip › EV_source_data/Figure_EV5/Figure_EV5.pdf]

Figure 1

# B

## 1#

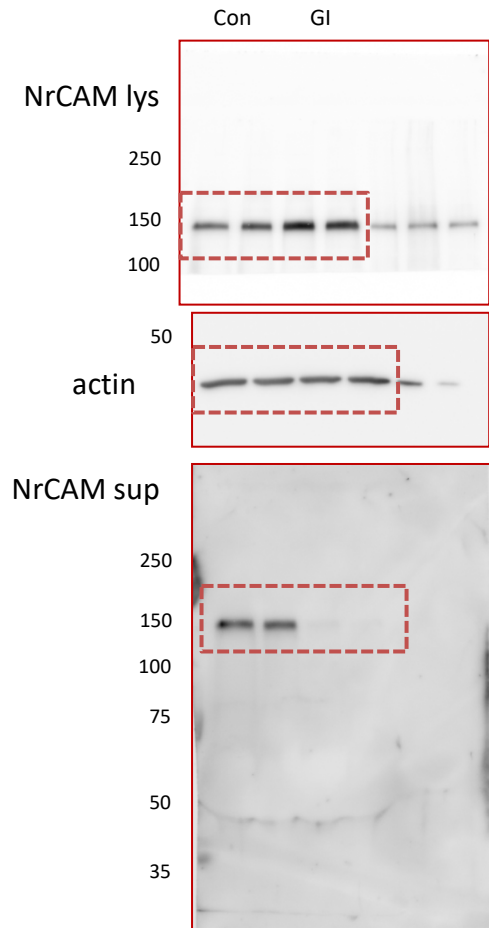

## 2#

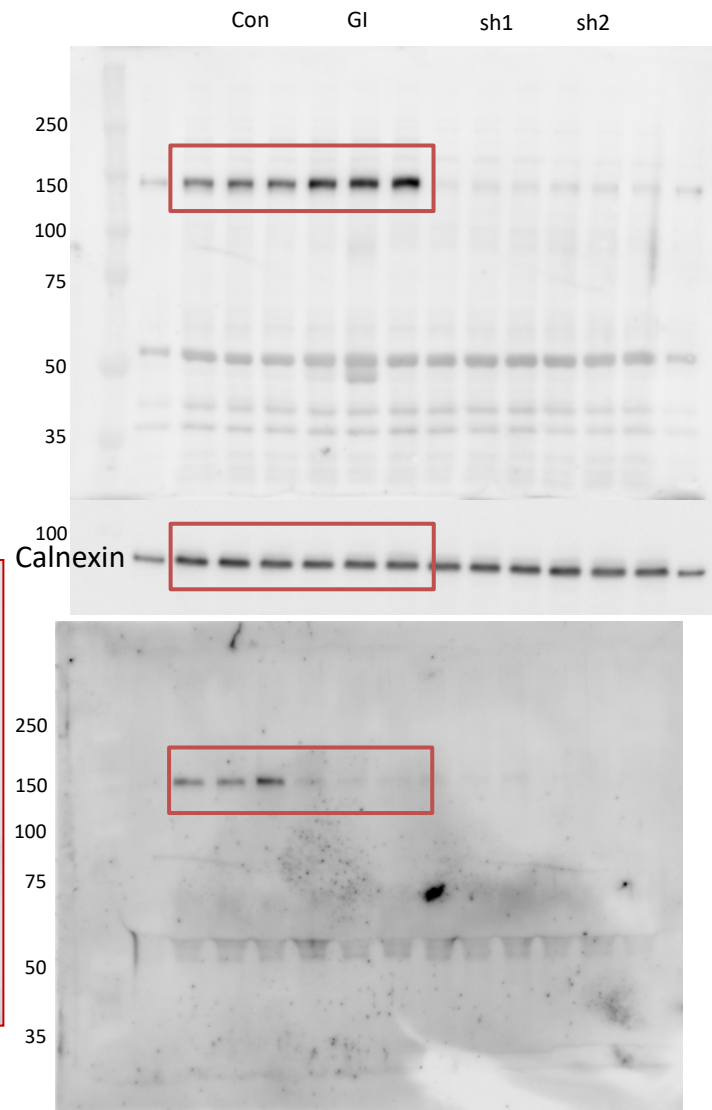

## 3#

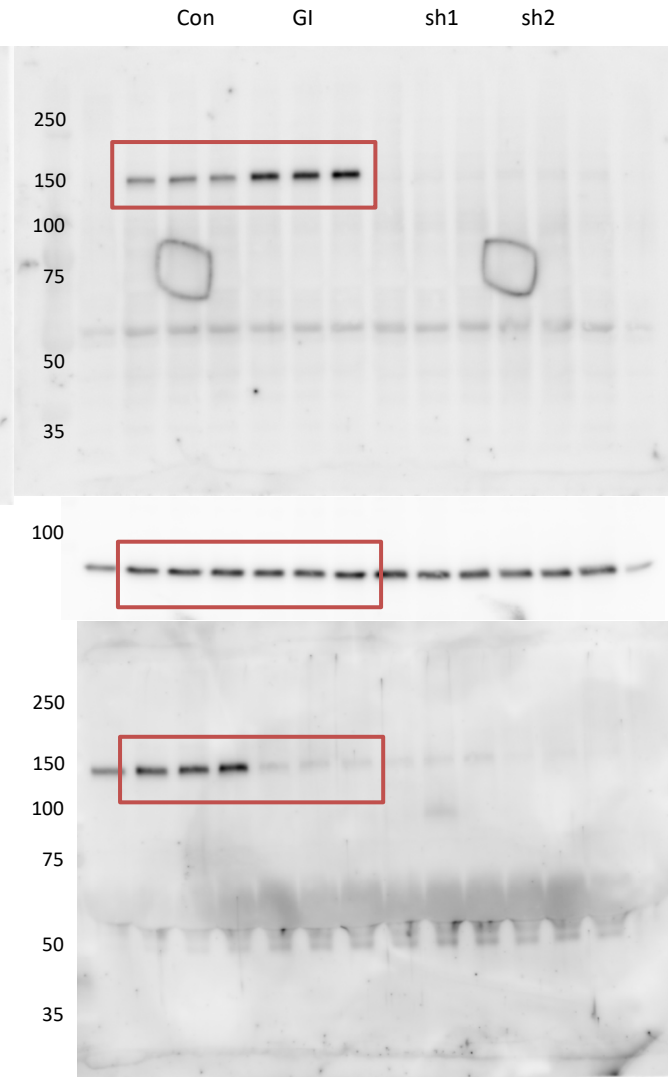

# B

1#

2#

3#

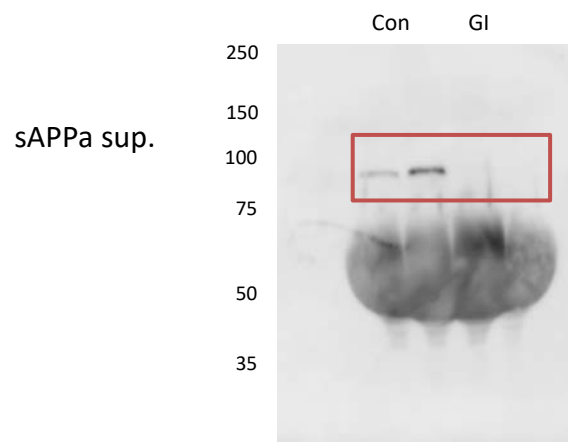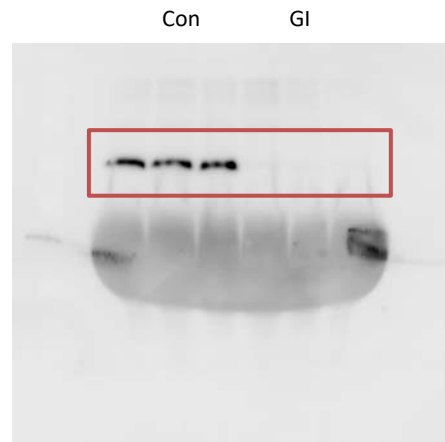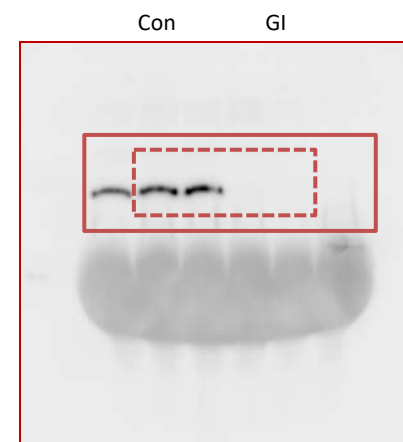

# C

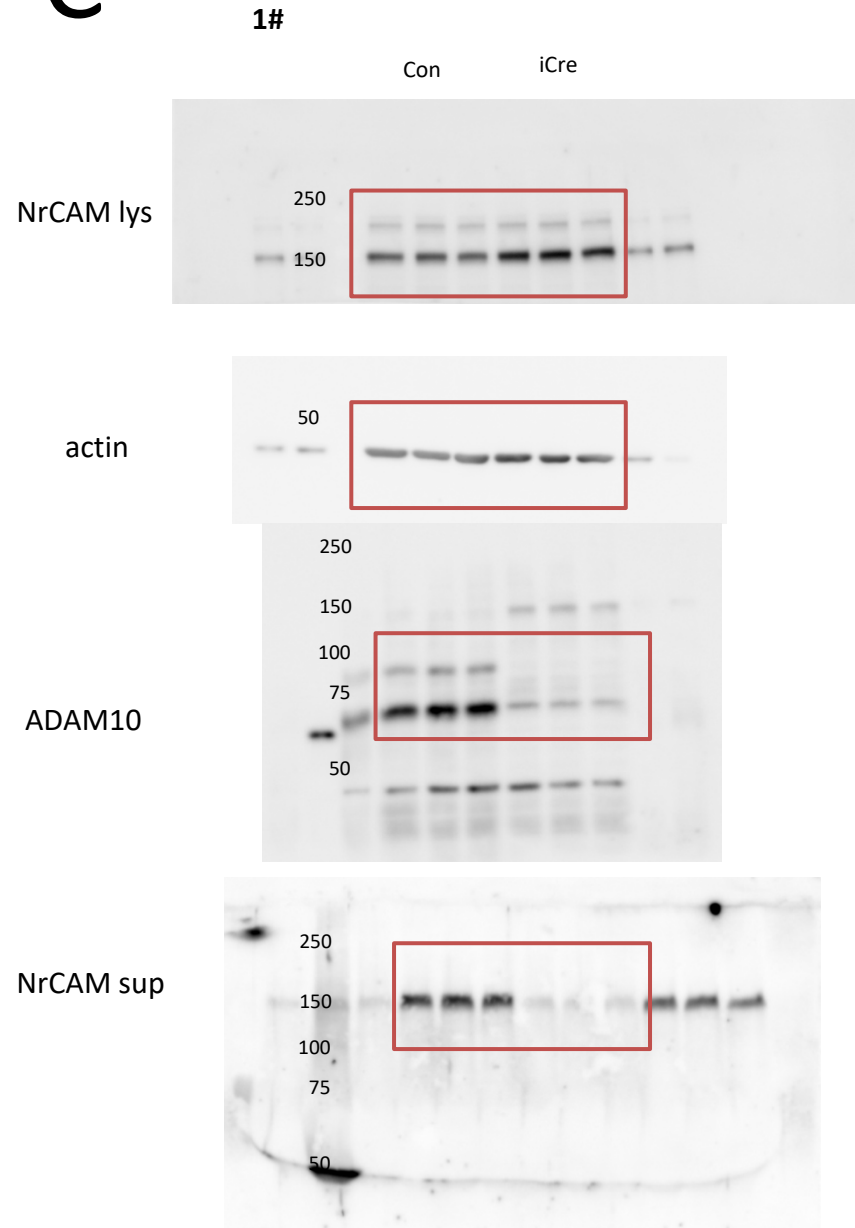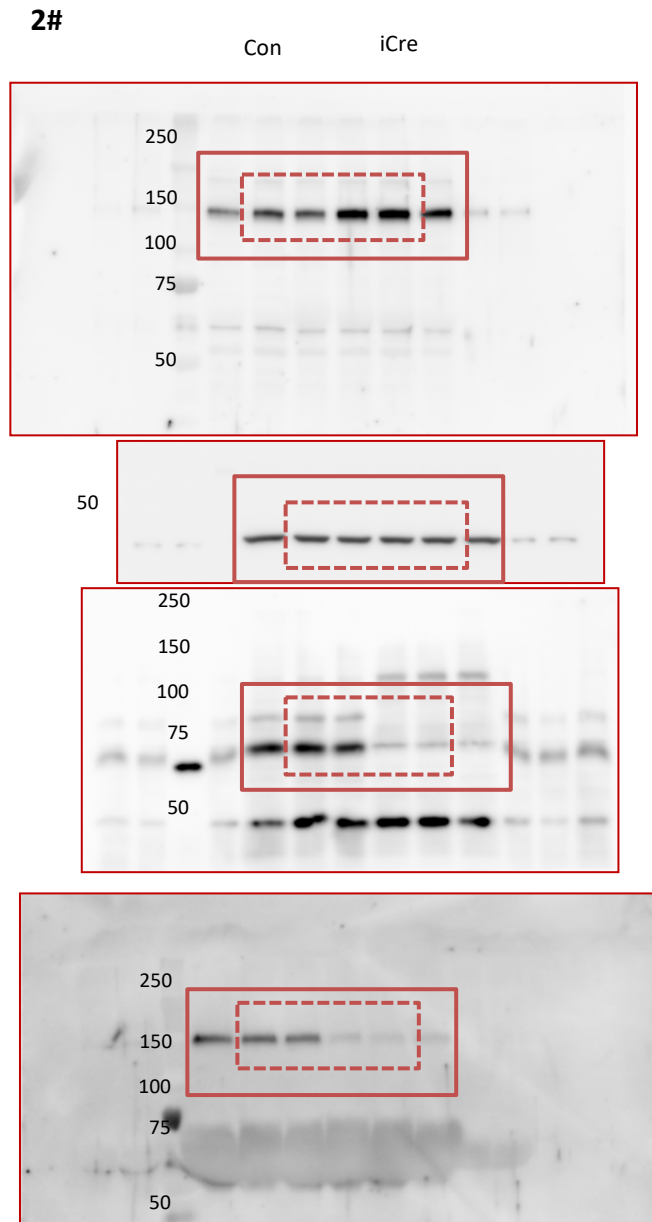

# C

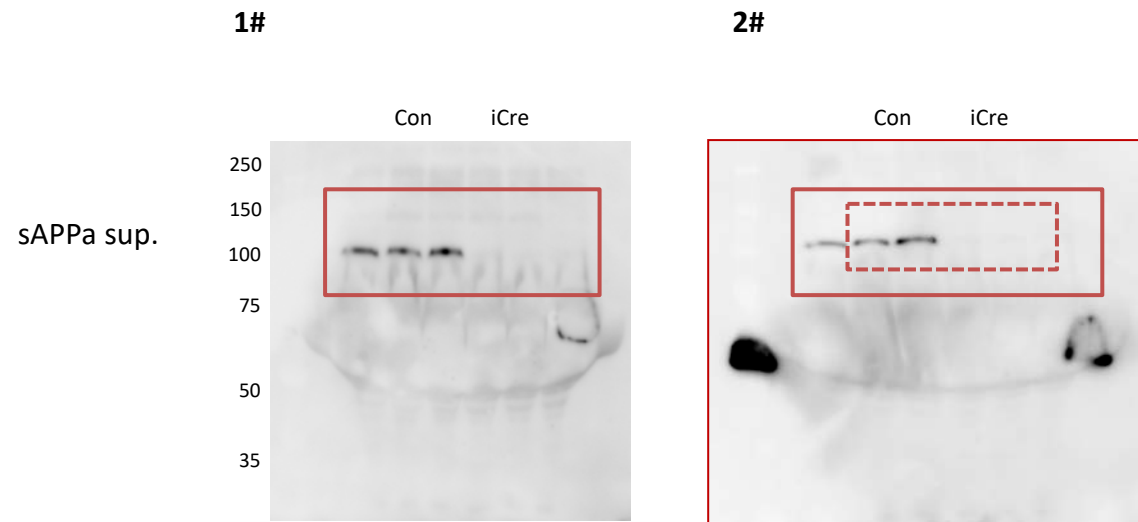

Supplement: Supplementary file 5 — Source Data for Figure 1 [file EMMM-11-e9695-s003.zip › Figure_1/Figure_1.pdf]

Figure 2

**1#**

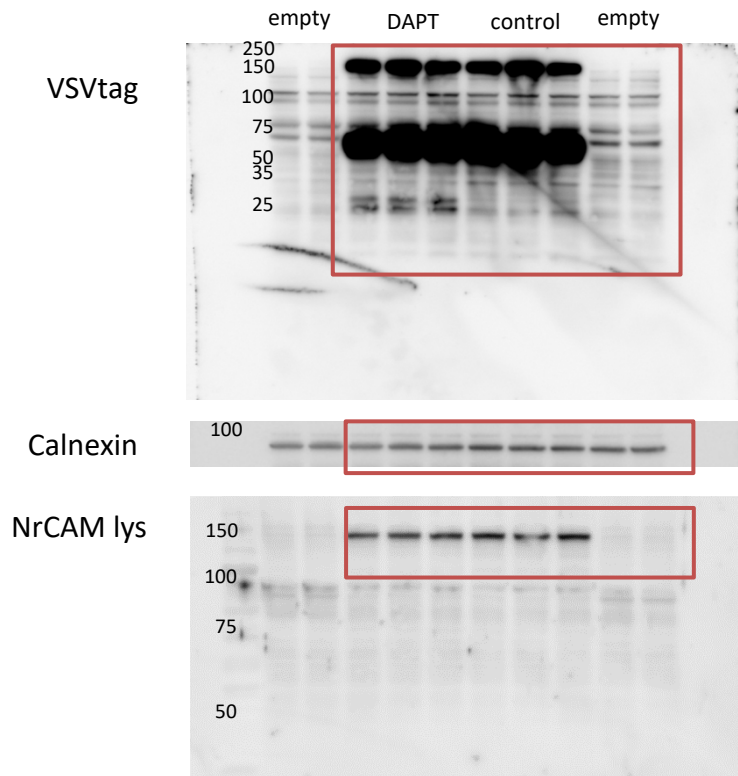

**2#**

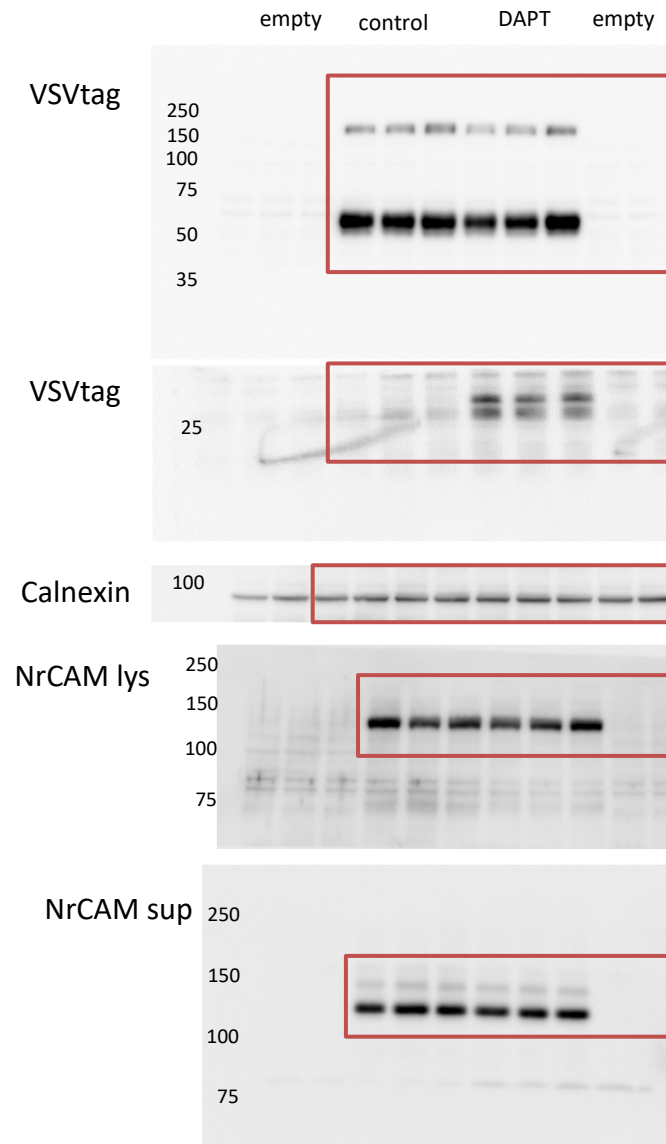

3#

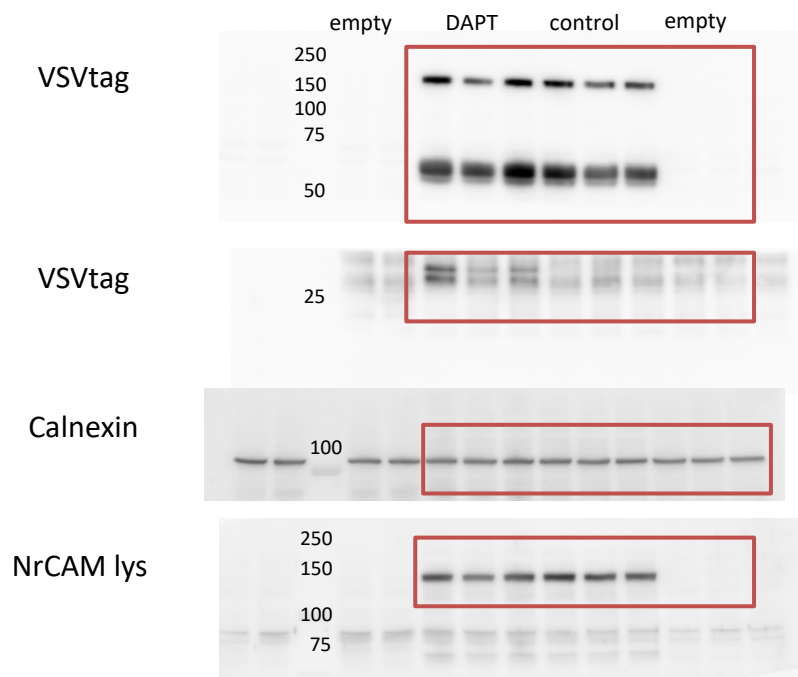

NrCAM sup

4#

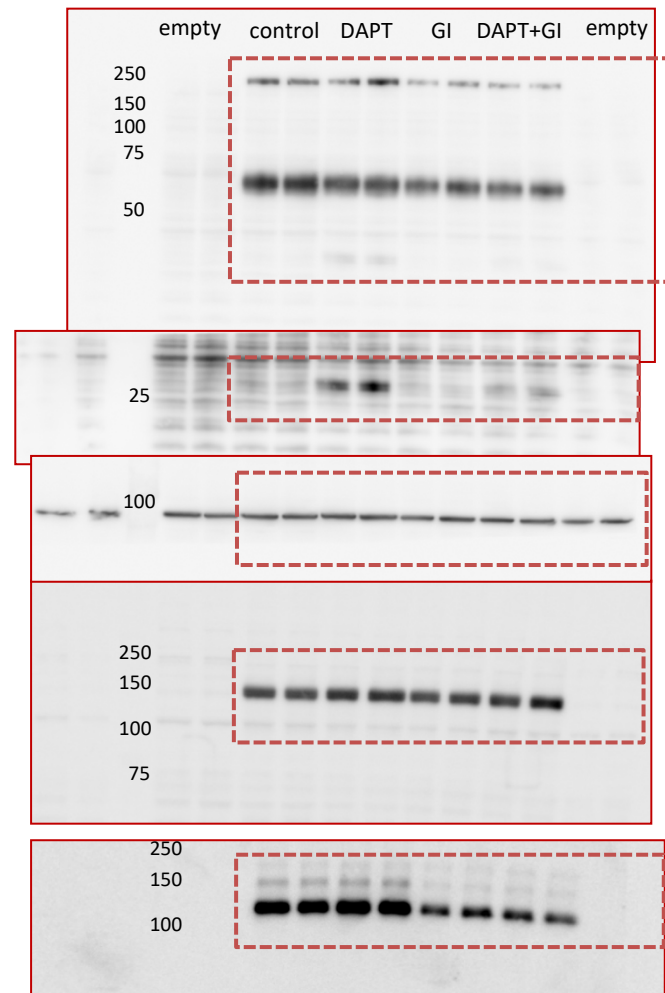

5#

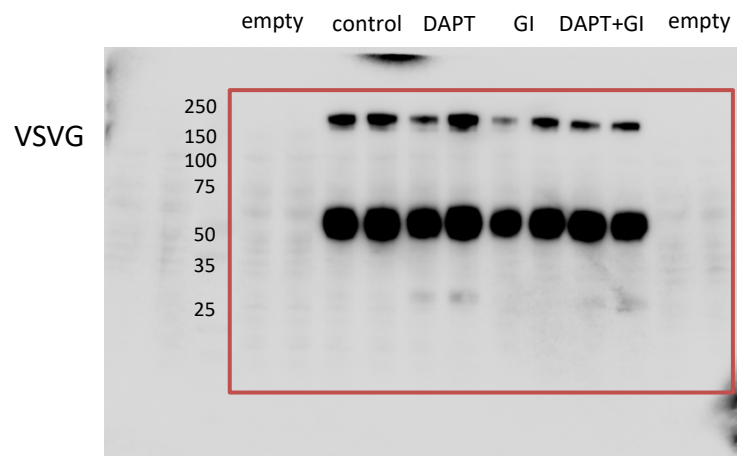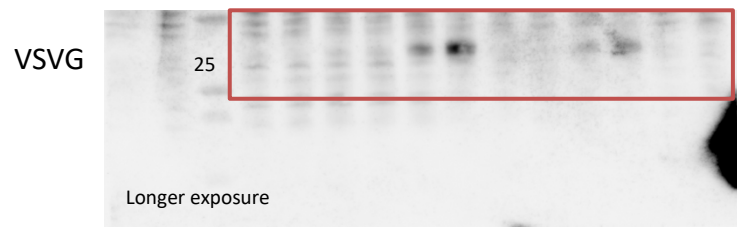

6#

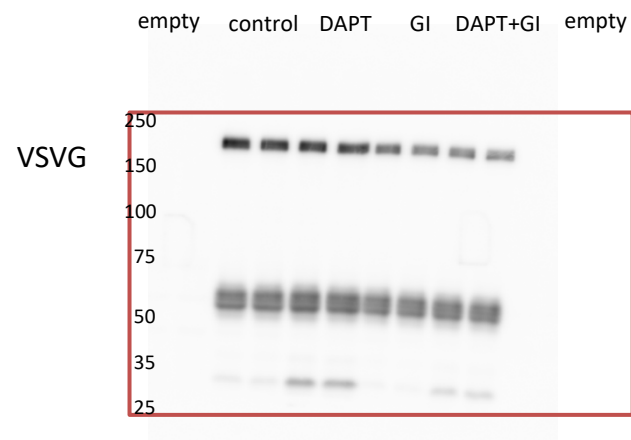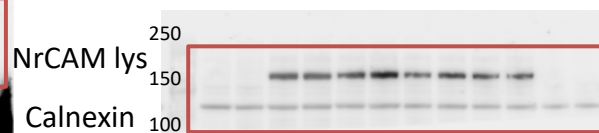

Supplement: Supplementary file 6 — Source Data for Figure 2 [file EMMM-11-e9695-s004.zip › Figure_2/Figure_2.pdf]

Figure 3

# A

1#

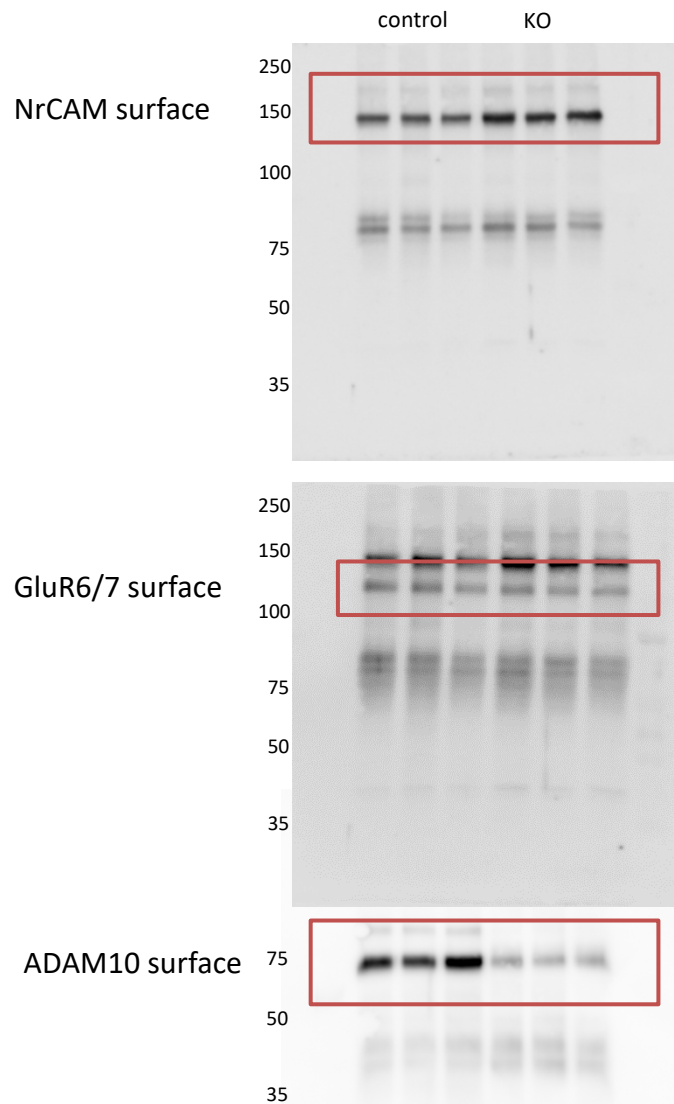

2#

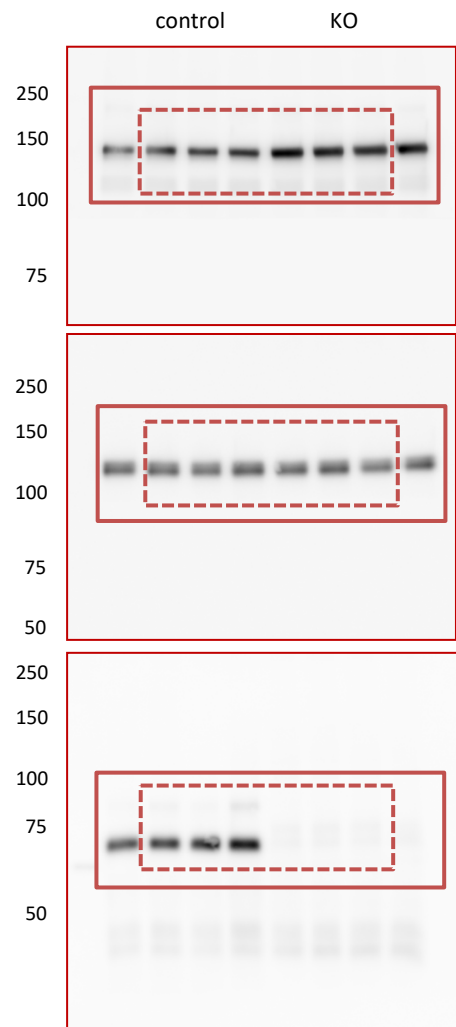

# A

1#

2#

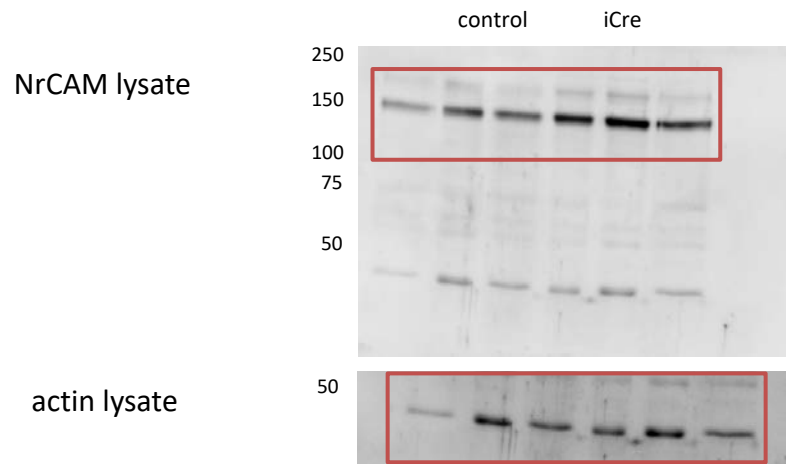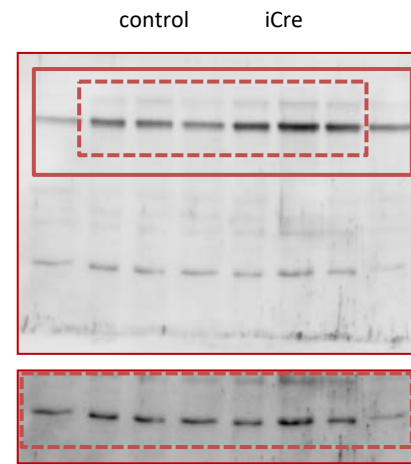

# B

1#

2#

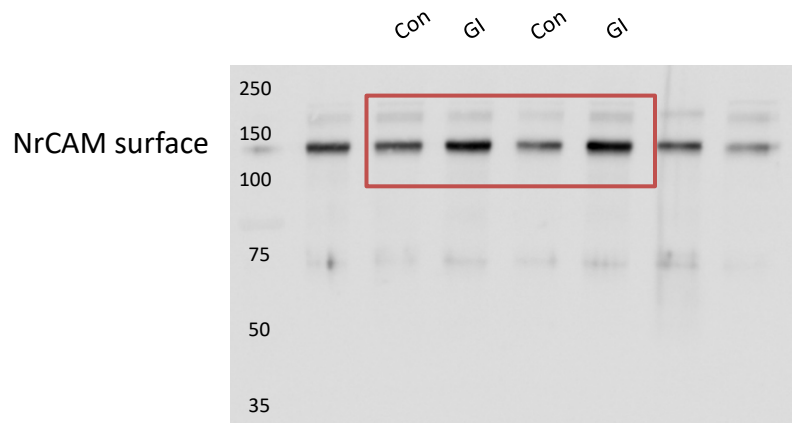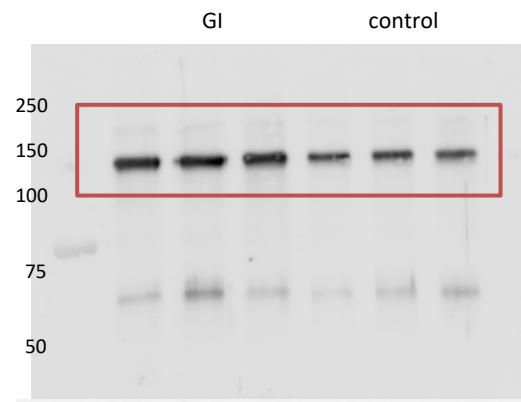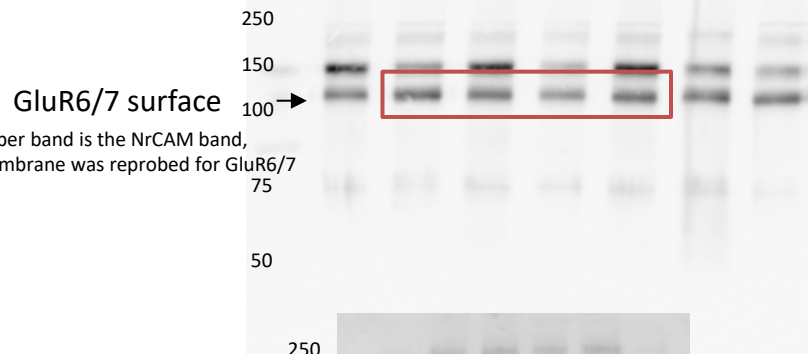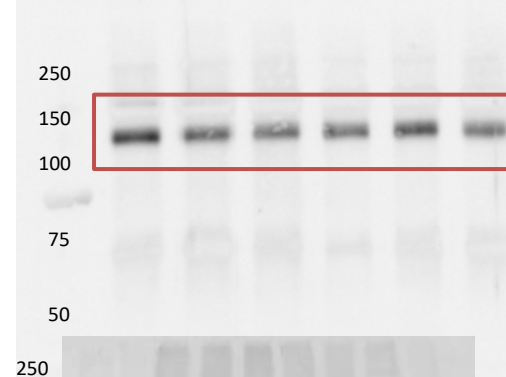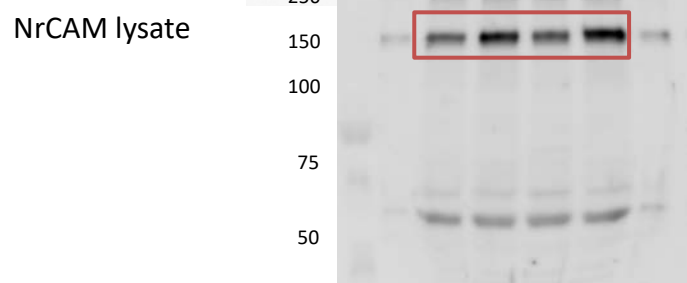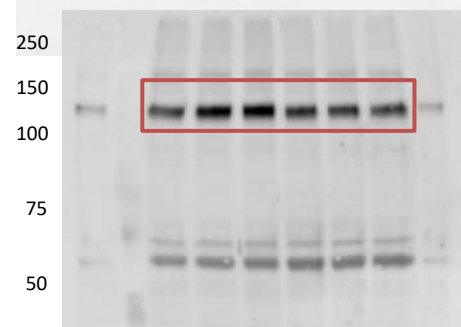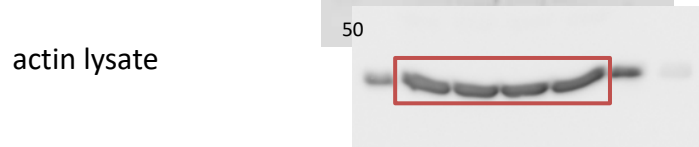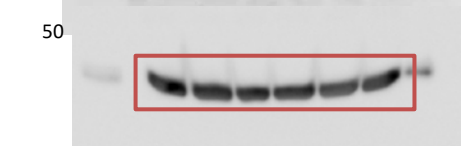

# B

## 3#

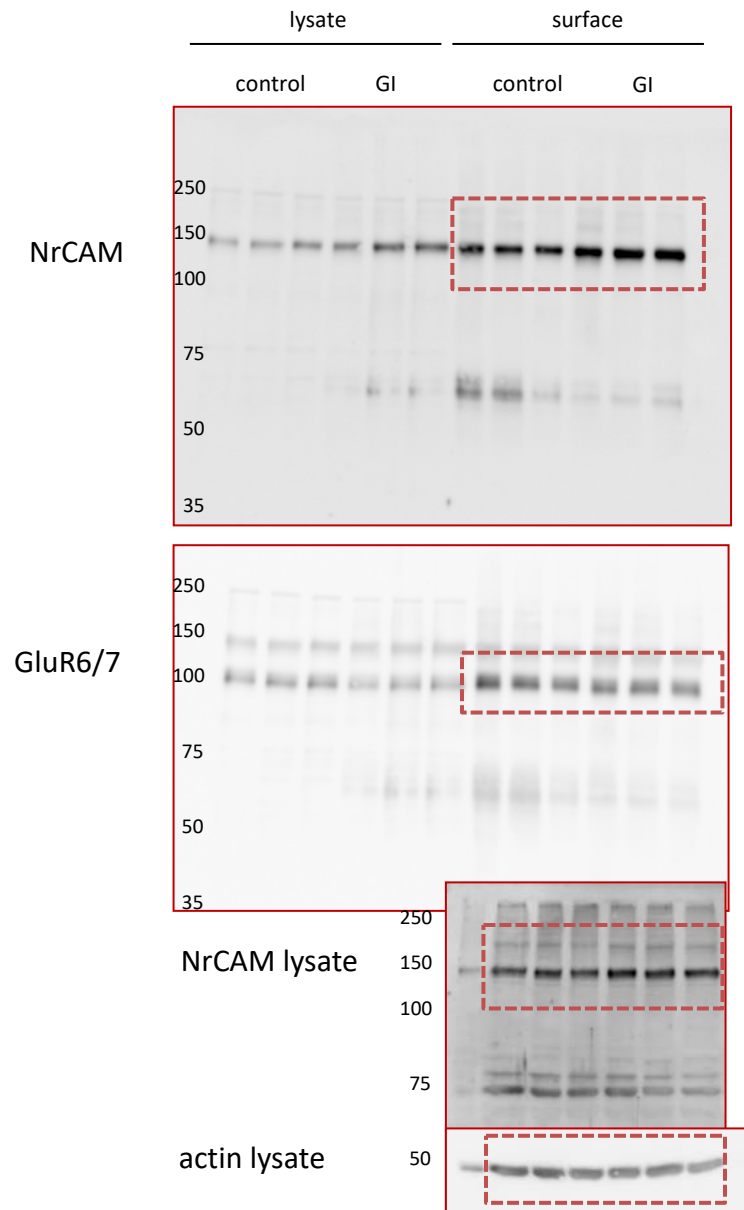

## 4#

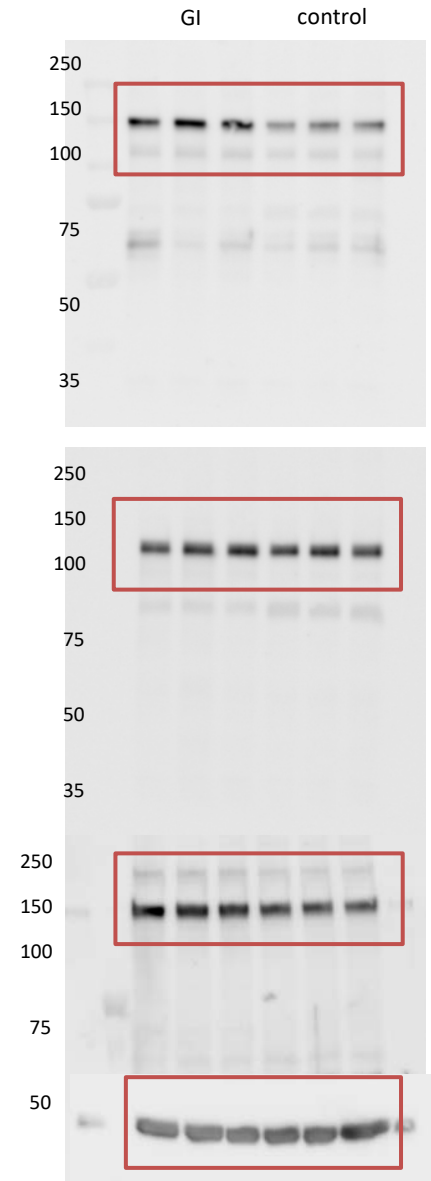

Supplement: Supplementary file 7 — Source Data for Figure 3 [file EMMM-11-e9695-s005.zip › Figure_3/Figure_3.pdf]

Figure 5

# A

1#

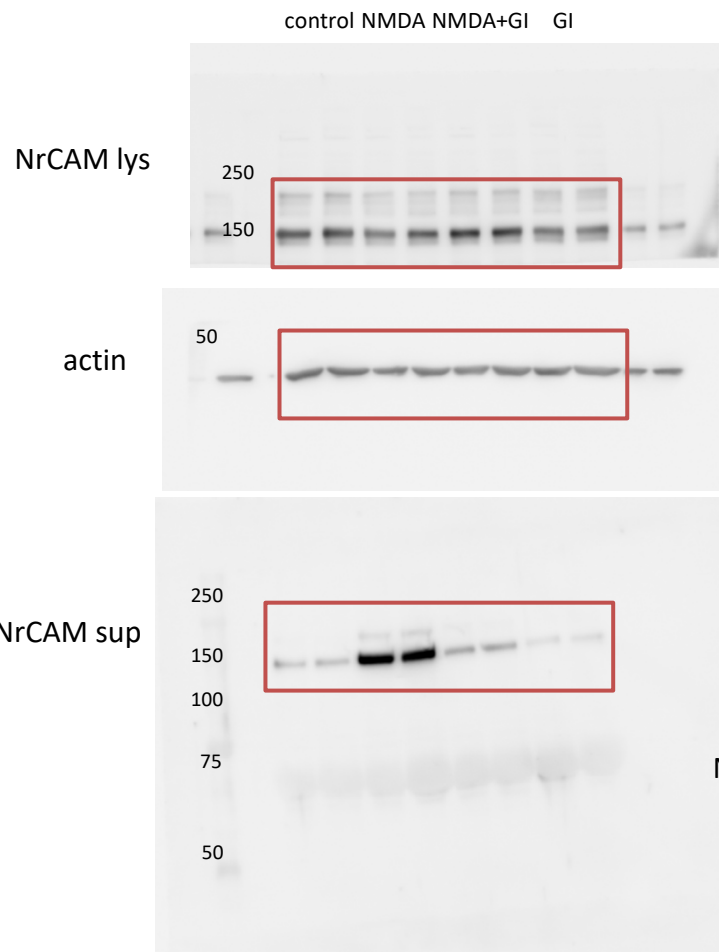

2#

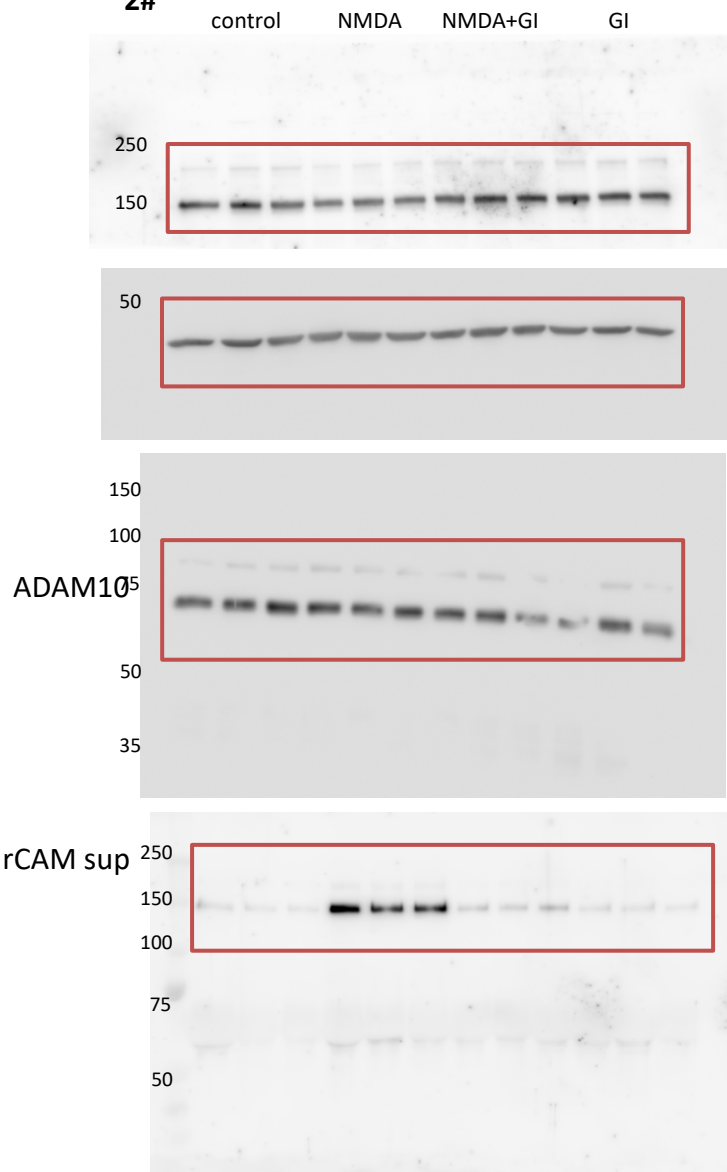

# A

3#

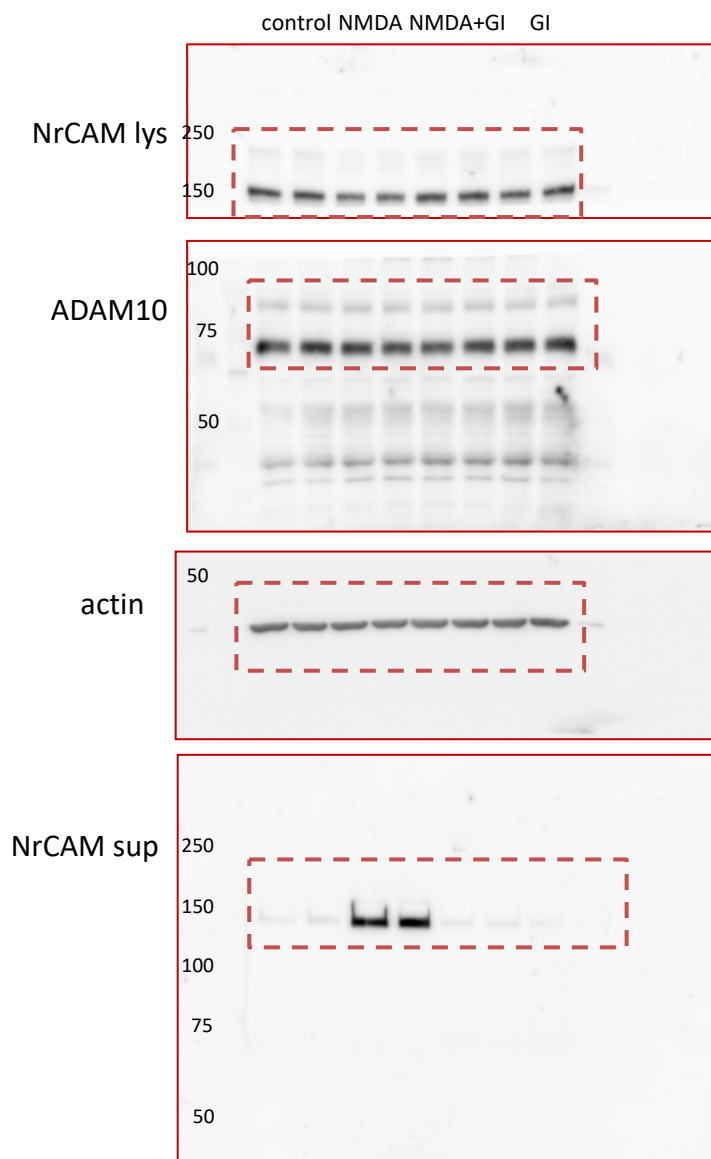

# B

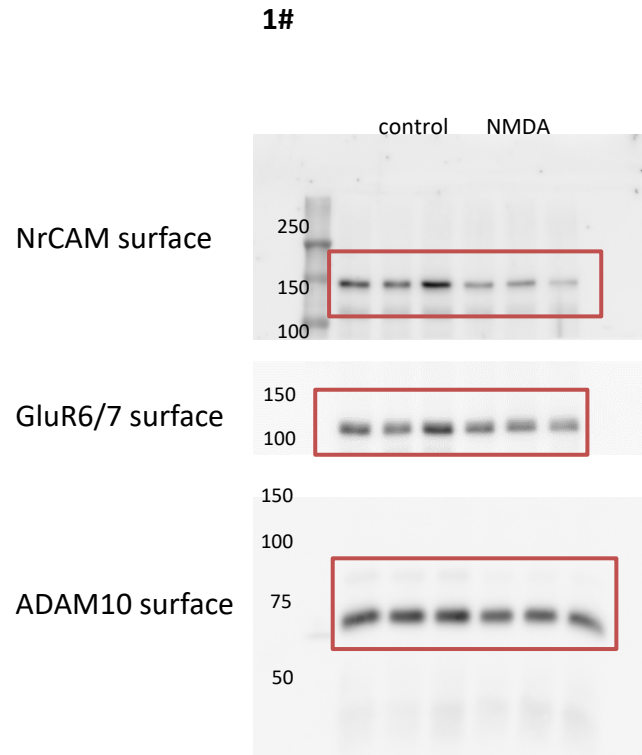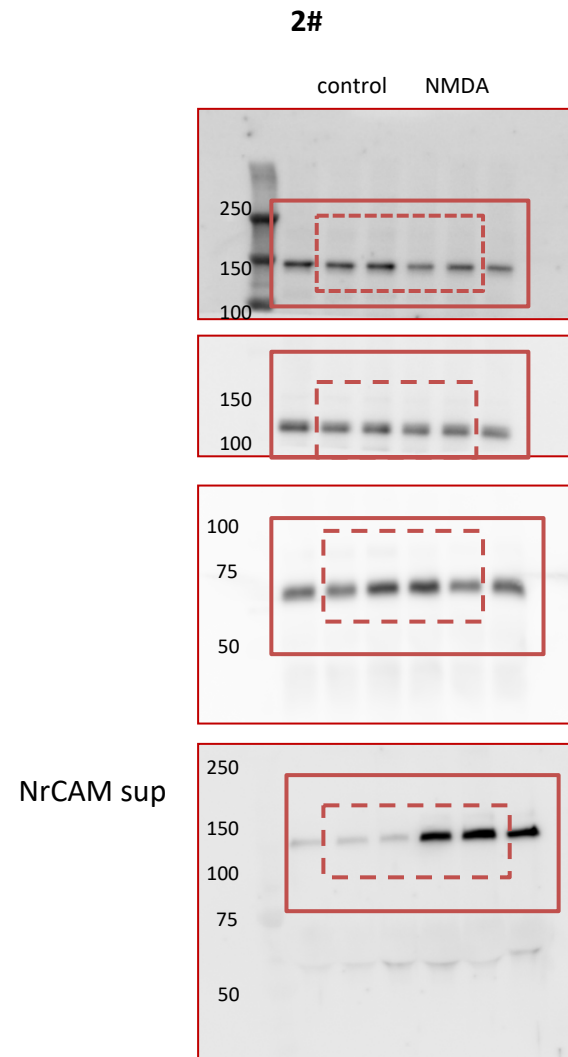

# B

1#

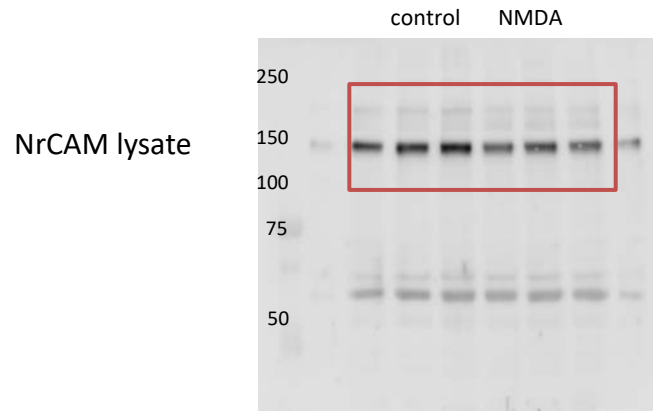

actin lysate

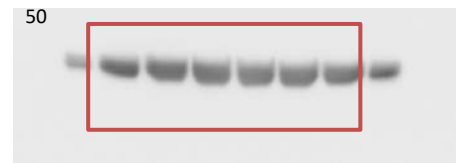

2#

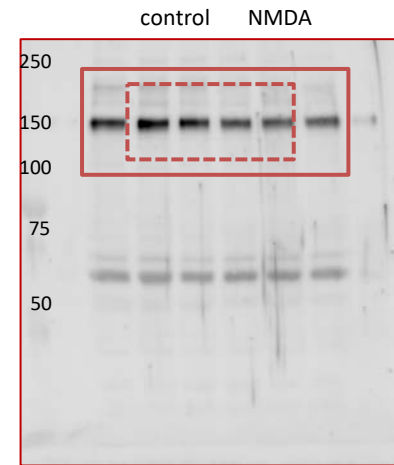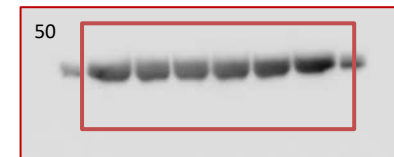

Supplement: Supplementary file 9 — Source Data for Figure 5 [file EMMM-11-e9695-s007.zip › Figure_5/Figure_5.pdf]

Figure 7

# #1

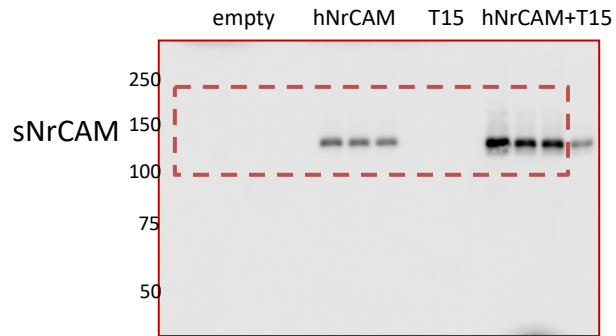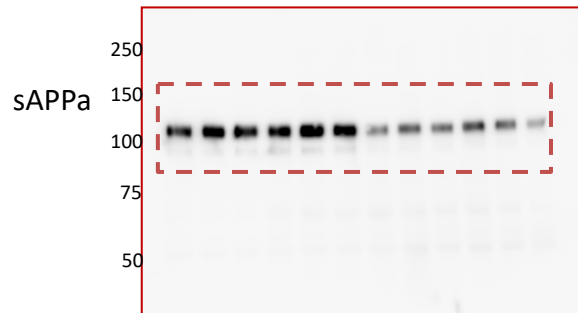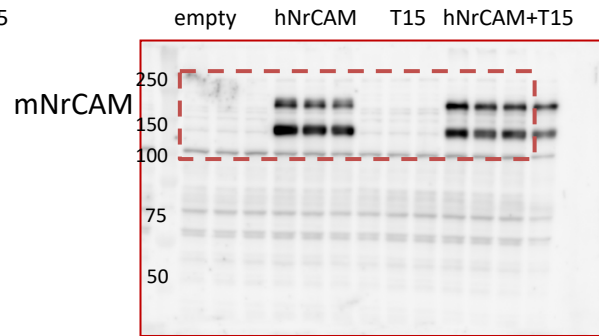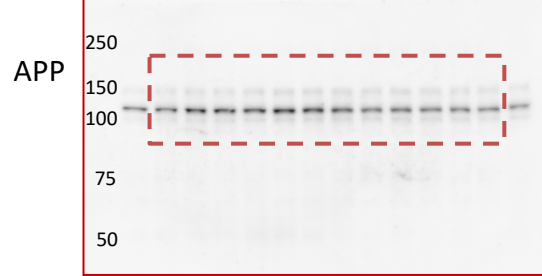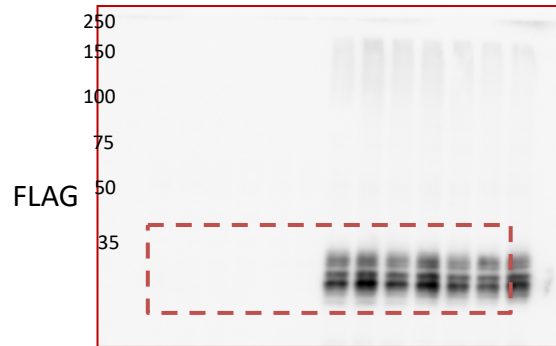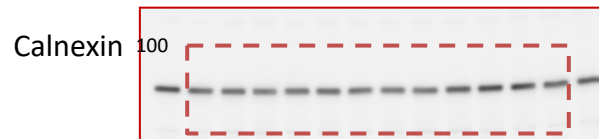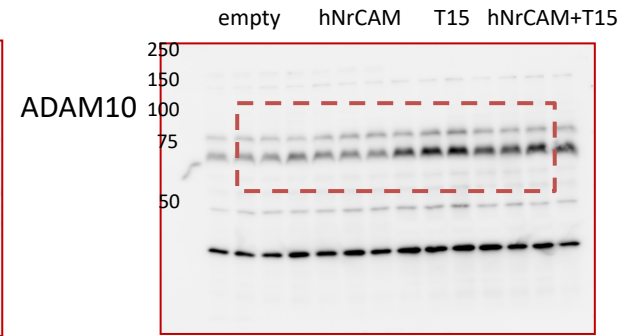

# #2

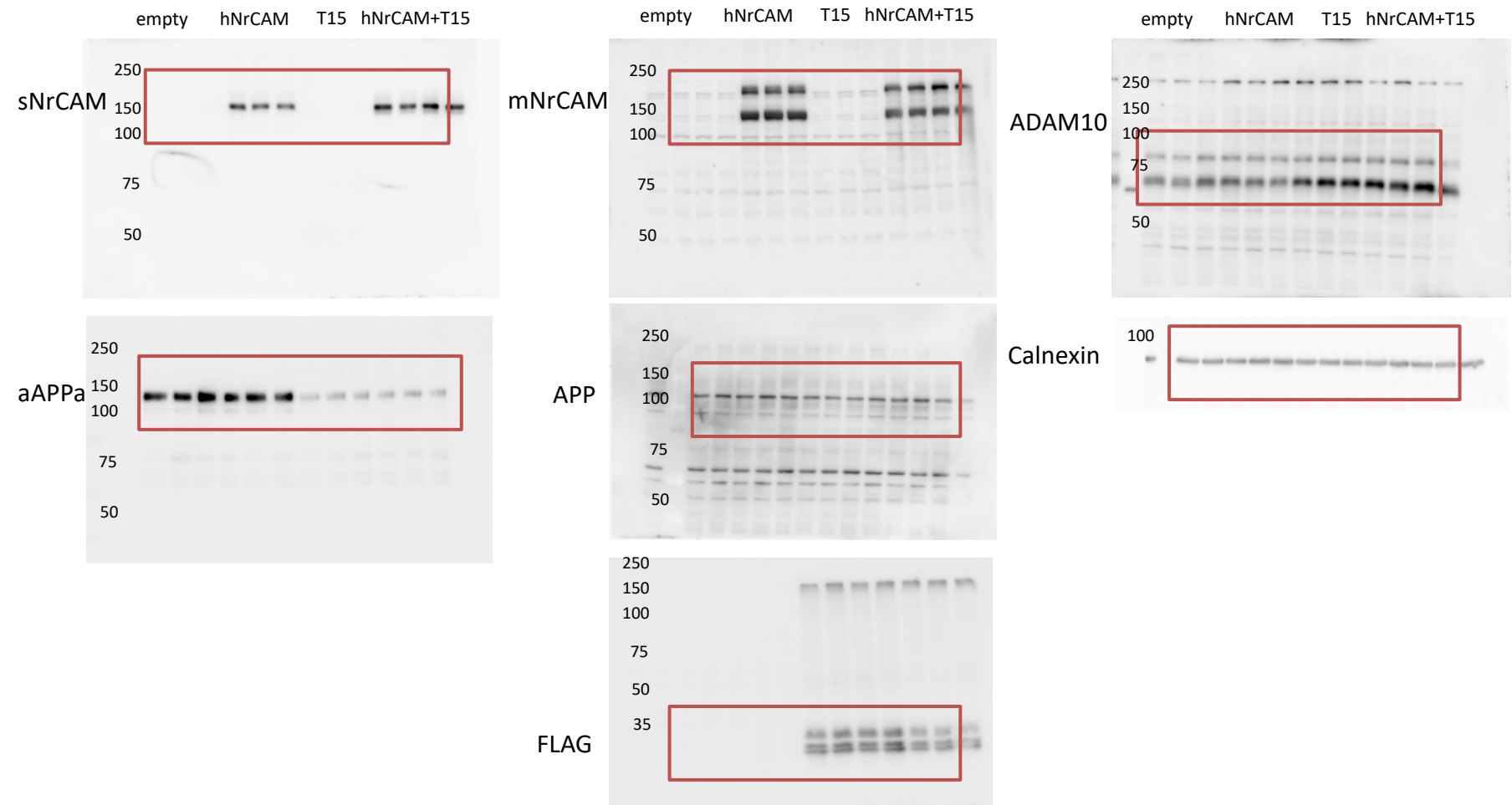

Supplement: Supplementary file 10 — Source Data for Figure 7 [file EMMM-11-e9695-s008.zip › Figure_7/Figure_7.pdf]
