# Supplementary material for: NrCAM is a marker for substrate‐selective activation of ADAM10 in Alzheimer's disease
Source: EMBO Mol Med. 2019 Mar 4;11(4):e9695. doi: 10.15252/emmm.201809695 (PMC6460357; doi:10.15252/emmm.201809695)
Supplement: Supplementary file 5 — Source Data for Figure 1 [file EMMM-11-e9695-s003.zip › Figure_1/Figure_1.docx]

**Figure 1:**

**Western blots:**

The red frame shows which blot was used for the generation of the final figure.

The red boxes show which lanes were used for the statistics.

The red dashed boxes show which representative part of the blot was chosen for the generation of the final figure.

Numbers on the left side indicate the apparent molecular weight in kDa.

Protein names are shown on the left side. Lys: lysate; sup: supernatant/conditioned media.

Figure 1B #2 and #3: sh1 and 2: KD of NrCAM with shRNAs.

**Graph Pad prism files:**

Statistics were calculated with the lanes indicated by red boxes (see above).

Normal distribution was assumed.

Statistics were calculated for sNrCAM, sAPPα and mNrCAM.

**Link for a free prism viewer:** https://www.graphpad.com/support/faqid/788/
