# Supplementary material for: NrCAM is a marker for substrate‐selective activation of ADAM10 in Alzheimer's disease
Source: EMBO Mol Med. 2019 Mar 4;11(4):e9695. doi: 10.15252/emmm.201809695 (PMC6460357; doi:10.15252/emmm.201809695)
Supplement: Supplementary file 8 — Source Data for Figure 4 [file EMMM-11-e9695-s006.zip › Figure_4/Figure_4.pdf]

Figure 4

A

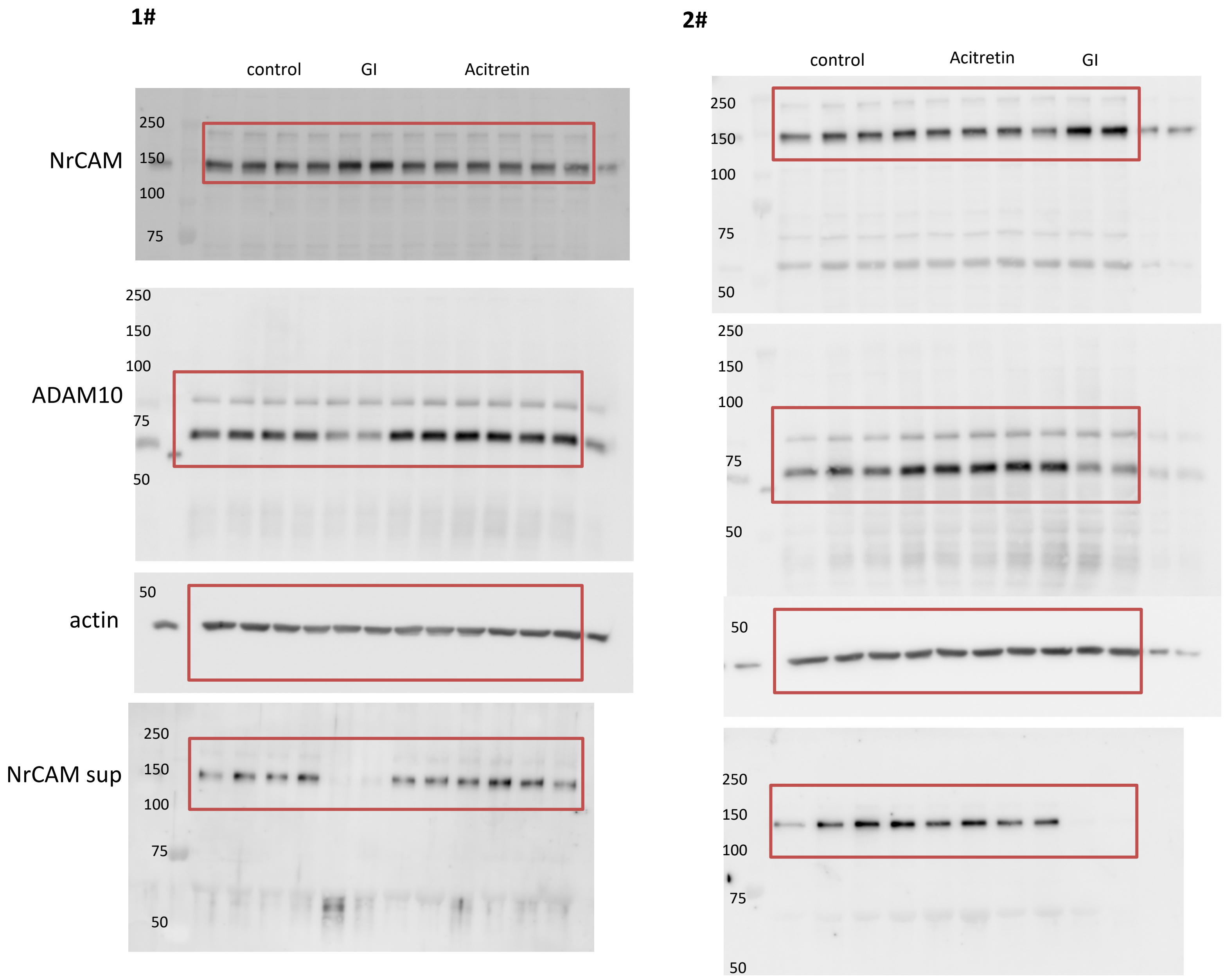
1#
control
GI
Acitretin
NrCAM
ADAM10
actin
NrCAM sup
250
150
100
75
50
2#
control
Acitretin
GI

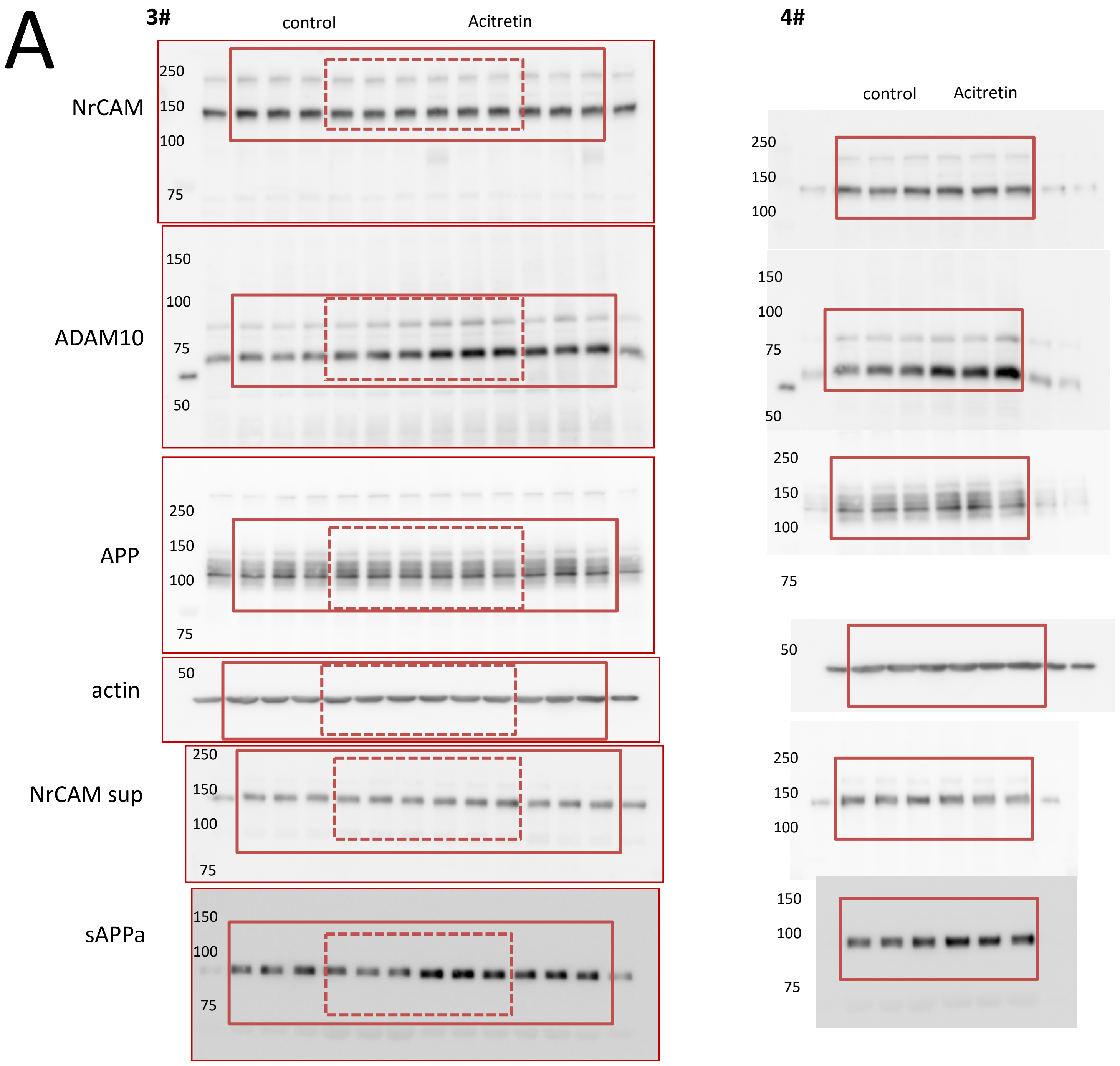
A
3#
control
Acitretin
4#
control
Acitretin
NrCAM
ADAM10
APP
actin
NrCAM sup
sAPPa
250
150
100
75
50

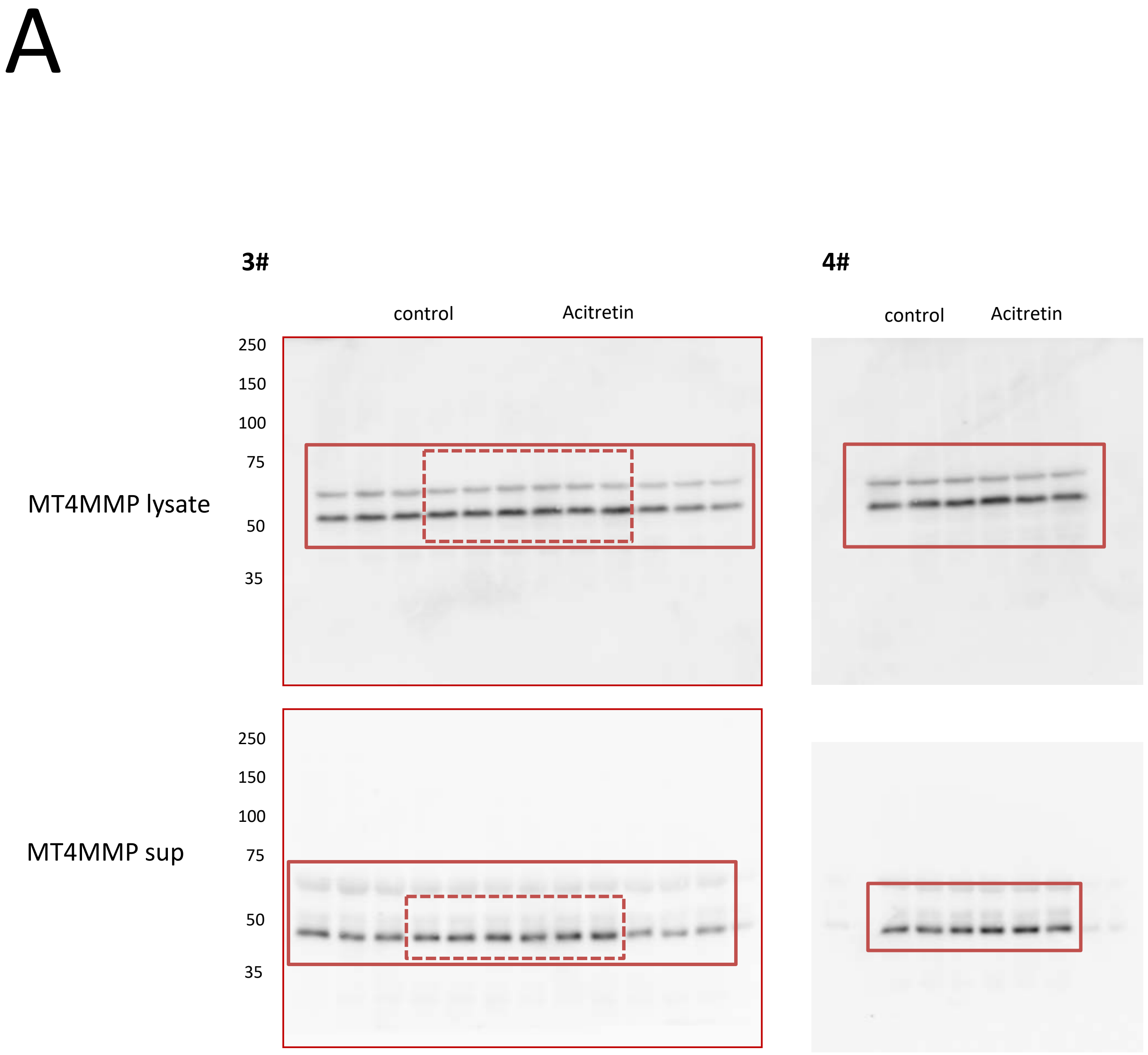
A
3#
4#
control
Acitretin
control
Acitretin
250
150
100
75
50
35
MT4MMP lysate
250
150
100
75
50
35
MT4MMP sup

C

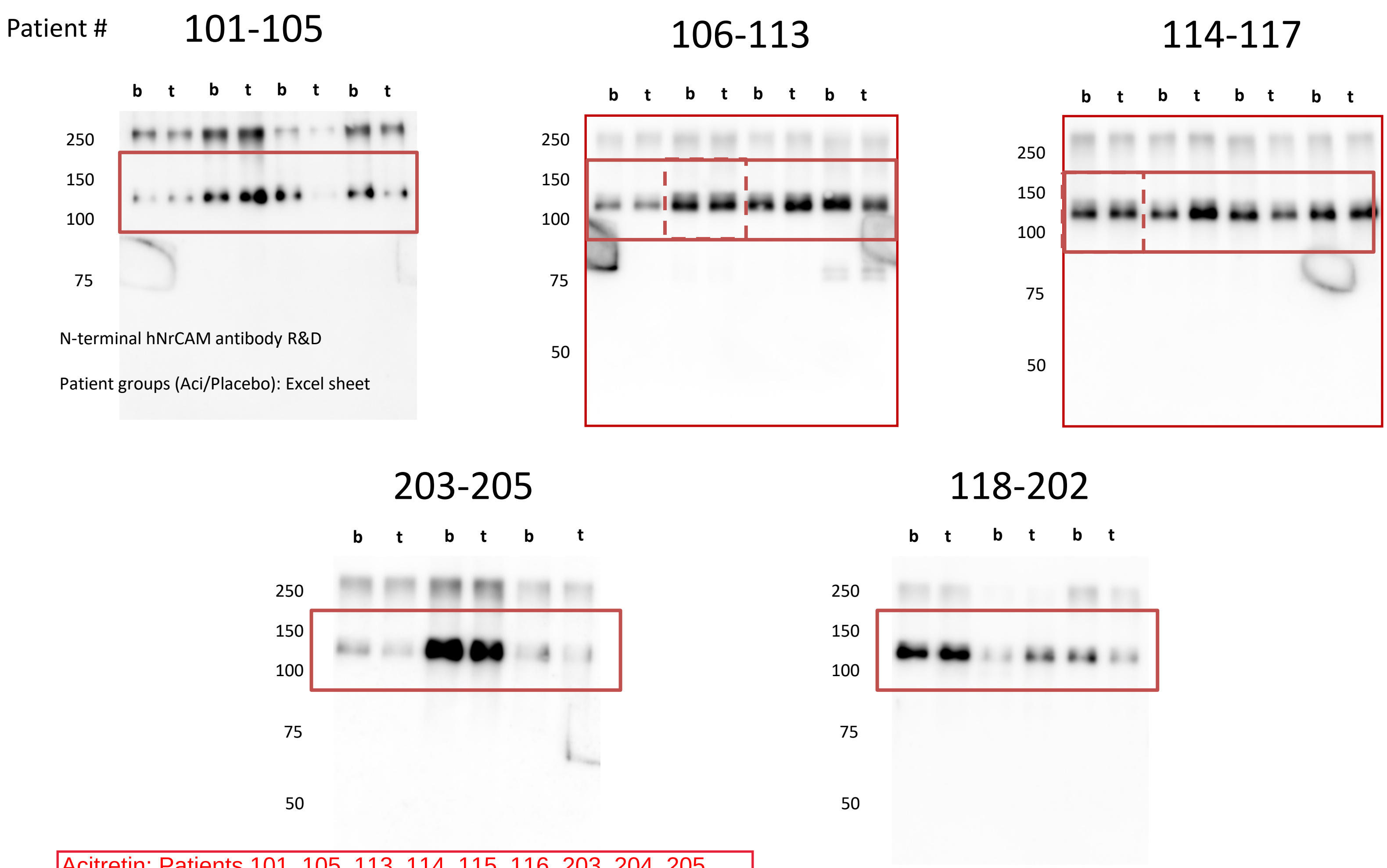

Acitretin: Patients 101, 105, 113, 114, 115, 116, 203, 204, 205

Placebo: Patients 102, 103, 106, 111, 112, 117, 118, 201, 202.

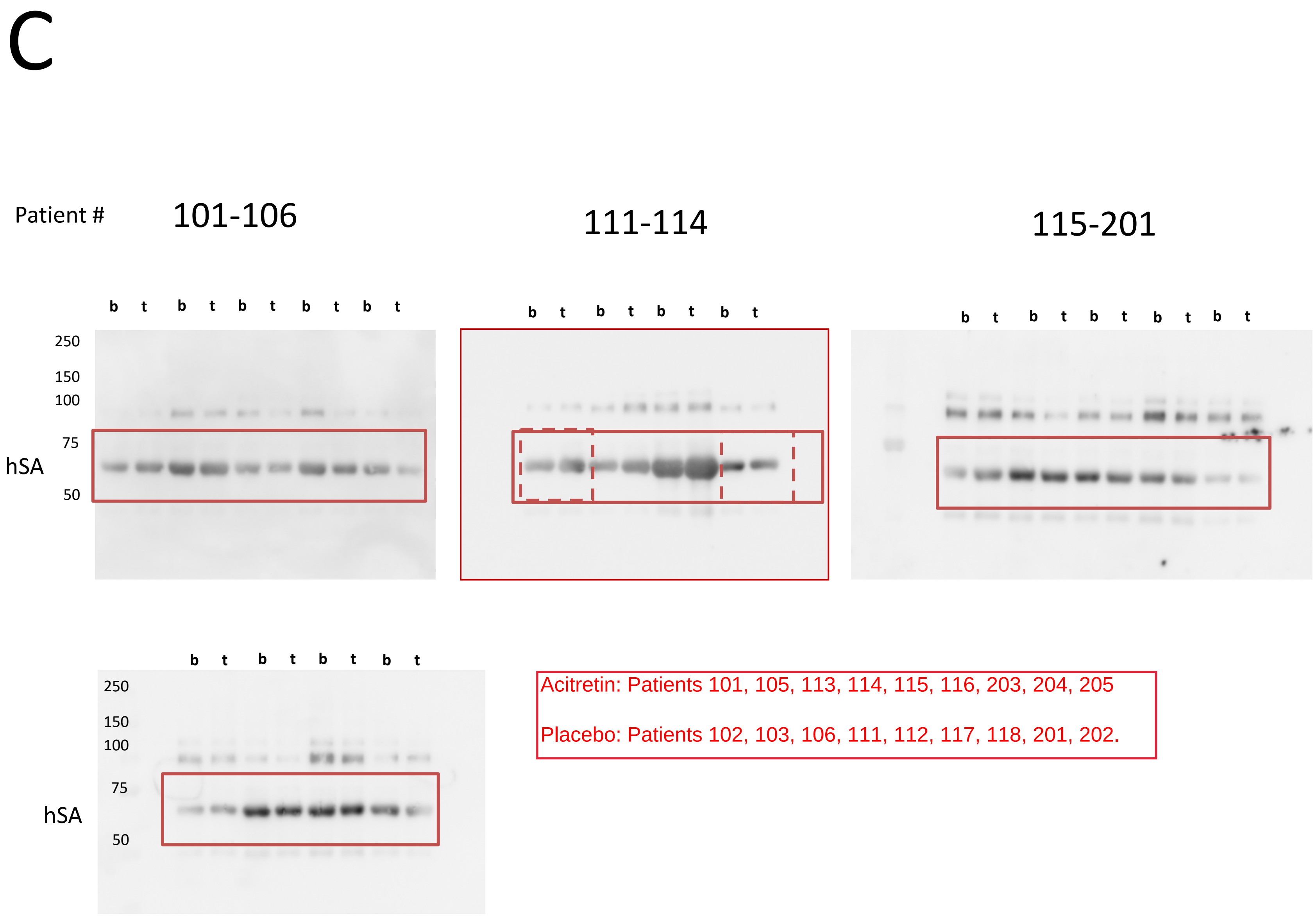
C
Patient #
101-106
111-114
115-201
b t b t b t b t b t
b t b t b t b t
b t b t b t b t b t
250
150
100
75
50
hSA
b t b t b t b t
250
150
100
75
50
hSA
Acitretin: Patients 101, 105, 113, 114, 115, 116, 203, 204, 205
Placebo: Patients 102, 103, 106, 111, 112, 117, 118, 201, 202.
